# Synthetic biology open language (SBOL) version 3.1.0

**DOI:** 10.1515/jib-2022-0058

**Published:** 2023-03-13

**Authors:** Lukas Buecherl, Thomas Mitchell, James Scott-Brown, Prashant Vaidyanathan, Gonzalo Vidal, Hasan Baig, Bryan Bartley, Jacob Beal, Matthew Crowther, Pedro Fontanarrosa, Thomas Gorochowski, Raik Grünberg, Vishwesh Kulkarni, James McLaughlin, Göksel Mısırlı, Ernst Oberortner, Anil Wipat, Chris Myers

**Affiliations:** University of Colorado Boulder, Boulder, USA; Raytheon BBN Technologies, Cambridge, USA; University of Edinburgh, Edinburgh, UK; Oxford Biomedica, Oxford, UK; Newcastle University, Newcastle upon Tyne, UK; University of Connecticut, Storrs, USA; University of Bristol, Bristol, UK; King Abdullah University for Science and Technology, Thuwal, SA; University of Warwick, Coventry, UK; Keele University, Newcastle, UK; DOE Joint Genome Institute, Berkeley, USA

## Abstract

Synthetic biology builds upon genetics, molecular biology, and metabolic engineering by applying engineering principles to the design of biological systems. When designing a synthetic system, synthetic biologists need to exchange information about multiple types of molecules, the intended behavior of the system, and actual experimental measurements. The *Synthetic Biology Open Language* (SBOL) has been developed as a standard to support the specification and exchange of biological design information in synthetic biology, following an open community process involving both bench scientists and scientific modelers and software developers, across academia, industry, and other institutions. This document describes SBOL 3.1.0, which improves on version 3.0.0 by including a number of corrections and clarifications as well as several other updates and enhancements. First, this version includes a complete set of validation rules for checking whether documents are valid SBOL 3. Second, the best practices section has been moved to an online repository that allows for more rapid and interactive of sharing these conventions. Third, it includes updates based upon six community approved enhancement proposals. Two enhancement proposals are related to the representation of an object’s namespace. In particular, the **Namespace** class has been removed and replaced with a **namespace** property on each class. Another enhancement is the generalization of the **CombinatorialDeriviation** class to allow direct use of **Features** and **Measures**. Next, the **Participation** class now allow **Interactions** to be **participants** to describe higher-order interactions. Another change is the use of *Sequence Ontology* terms for **Feature orientation**. Finally, this version of SBOL has generalized from using Unique Reference Identifiers (URIs) to *Internationalized Resource Identifiers* (IRIs) to support international character sets.

This document does not contain technology or technical data controlled under either the U.S. International Traffic in Arms Regulations or the U.S. Export Administration Regulations.

***Corresponding author: Chris Myers**, University of Colorado Boulder, Boulder, USA, E-mail: chris.myers@colorado.edu
**Lukas Buecherl and Pedro Fontanarrosa**, University of Colorado Boulder, Boulder, USA. https://orcid.org/0000-0002-4844-6605 (L. Buecherl), https://orcid.org/0000-0002-0535-2684 (P. Fontanarrosa)
**Thomas Mitchell, Bryan Bartley and Jacob Beal**, Raytheon BBN Technologies, Cambridge, USA. https://orcid.org/0000-0002-1663-5102 (J. Beal)
**James Scott-Brown**, University of Edinburgh, Edinburgh, UK. https://orcid.org/0000-0001-5642-8346
**Prashant Vaidyanathan**, Oxford Biomedica, Oxford, UK
**Gonzalo Vidal, Matthew Crowther, James McLaughlin and Anil Wipat**, Newcastle University, Newcastle upon Tyne, UK. https://orcid.org/0000-0003-3543-520X (G. Vidal)
**Hasan Baig**, University of Connecticut, Storrs, USA
**Thomas Gorochowski**, University of Bristol, Bristol, UK
**Raik Grünberg**, King Abdullah University for Science and Technology, Thuwal, SA
**Vishwesh Kulkarni**, University of Warwick, Coventry, UK
**Göksel Mısırlı**, Keele University, Newcastle, UK
**Ernst Oberortner**, DOE Joint Genome Institute, Berkeley, USA

## Synthetic Biology Open Language (SBOL) Version 3.1.0

**Editors:**

| | |
|---|---|
| Lukas Buecherl | *University of Colorado Boulder, USA* |
| Thomas Mitchell | *Raytheon BBN Technologies, USA* |
| James Scott-Brown | *University of Edinburgh, UK* |
| Prashant Vaidyanathan | *Oxford Biomedica, UK* |
| Gonzalo Vidal Peña | *Newcastle University, UK* |

editors@sbolstandard.org

**Chair:**

| | |
|---|---|
| Chris Myers | *University of Colorado Boulder, USA* |

**Additional authors:**

| | |
|---|---|
| Hasan Baig | *University of Connecticut, USA* |
| Bryan Bartley | *Raytheon BBN Technologies, USA* |
| Jacob Beal | *Raytheon BBN Technologies, USA* |
| Matthew Crowther | *Newcastle University, UK* |
| Pedro Fontanarrosa | *University of Colorado Boulder, USA* |
| Thomas Gorochowski | *University of Bristol, UK* |
| Raik Grünberg | *KAUST, SA* |
| Vishwesh Kulkarni | *University of Warwick, UK* |
| James McLaughlin | *Newcastle University, UK* |
| Goksel Misirli | *Keele University, UK* |
| Ernst Oberortner | *DOE Joint Genome Institute, USA* |
| Anil Wipat | *Newcastle University, UK* |

Version 3.1.0

October 26, 2022

SBOL
Synthetic Biology Open Language

## Contents

**1 Purpose** 4

**2 A Brief History of SBOL** 6

**3 Overview of SBOL** 8

**4 Conventions** 10
- 4.1 Terminology Conventions . . . 10
- 4.2 UML Diagram Conventions . . . 10
- 4.3 Naming and Typographic Conventions . . . 11

**5 Identifiers and Types** 12
- 5.1 Internationalized Resource Identifiers . . . 12
- 5.2 SBOL URLs . . . 12
- 5.3 Primitive Data Types . . . 12
- 5.4 SBOL Types . . . 13
- 5.5 Object Closure and Document Composition . . . 13

**6 SBOL Data Model** 15
- 6.1 Identified . . . 15
- 6.2 TopLevel . . . 16
- 6.3 Sequence . . . 16
- 6.4 Component . . . 18
  - 6.4.1 Feature . . . 22
    - 6.4.1.1 SubComponent . . . 23
    - 6.4.1.2 ComponentReference . . . 24
    - 6.4.1.3 LocalSubComponent . . . 25
    - 6.4.1.4 ExternallyDefined . . . 25
    - 6.4.1.5 SequenceFeature . . . 25
  - 6.4.2 Location . . . 26
    - 6.4.2.1 Range . . . 27
    - 6.4.2.2 Cut . . . 27
    - 6.4.2.3 EntireSequence . . . 27
  - 6.4.3 Constraint . . . 27
  - 6.4.4 Interaction . . . 28
    - 6.4.4.1 Participation . . . 31
  - 6.4.5 Interface . . . 32
- 6.5 CombinatorialDerivation . . . 33
  - 6.5.1 VariableFeature . . . 34
- 6.6 Implementation . . . 36
- 6.7 ExperimentalData . . . 37
- 6.8 Model . . . 37
- 6.9 Collection . . . 38
  - 6.9.1 Experiment . . . 39
- 6.10 Attachment . . . 39
- 6.11 Annotation and Extension of SBOL . . . 40

**7 Recommended Best Practices** 41
- 7.1 SBOL Versions . . . 41
- 7.2 Compliant SBOL Objects . . . 41
- 7.3 Versioning SBOL Objects . . . 42
- 7.4 Annotations: Embedded Objects vs. External References . . . 42
- 7.5 Completeness and Validation . . . 42
- 7.6 Recommended Ontologies for External Terms . . . 42
- 7.7 Annotating Entities with Date & Time . . . 43
- 7.8 Annotating Entities with Authorship information . . . 43
- 7.9 Host Context / Ontologies for Experiments . . . 43
  - 7.9.1 Mixtures via Components . . . 43
  - 7.9.2 Media, Inducers, and Other Reagents . . . 43
  - 7.9.3 Samples . . . 44
  - 7.9.4 Other Experimental Parameters . . . 44
- 7.10 Multicellular System Designs . . . 44
  - 7.10.1 Representing Cell Types . . . 45
  - 7.10.2 Multiple Cell Types in a Single Design . . . 45
  - 7.10.3 Cell Ratios . . . 45

**8 SBOL RDF Serialization** 48

**9 SBOL Compliance** 49

**10 Mapping Between SBOL 1, SBOL 2, and SBOL3** **50**
10.1 Mapping between SBOL 1 and SBOL 2 . . . . . 50
10.2 Mapping between SBOL 2 and SBOL 3 . . . . . 50
**References** **54**
**A Complementary Standards** **55**
A.1 Adding Provenance with PROV-O . . . . . 55
A.1.1 prov:Activity . . . . . 57
A.1.2 prov:Usage . . . . . 58
A.1.3 prov:Association . . . . . 58
A.1.4 prov:Plan . . . . . 59
A.1.5 prov:Agent . . . . . 59
A.2 Adding Measures/Parameters with OM . . . . . 61
A.2.1 om:Measure . . . . . 61
A.2.2 om:Unit . . . . . 62
A.2.3 om:SingularUnit . . . . . 63
A.2.4 om:CompoundUnit . . . . . 63
A.2.5 om:UnitMultiplication . . . . . 63
A.2.6 om:UnitDivision . . . . . 64
A.2.7 om:UnitExponentiation . . . . . 64
A.2.8 om:PrefixedUnit . . . . . 64
A.2.9 om:Prefix . . . . . 64
A.2.10 om:SIPrefix . . . . . 65
A.2.11 om:BinaryPrefix . . . . . 65
**B Validation Rules** **66**

## 1 Purpose

Synthetic biology builds upon genetics, molecular biology, and metabolic engineering by applying engineering principles to the design of biological systems. When designing a synthetic system, synthetic biologists need to exchange information about multiple types of molecules, the intended behavior of the system, and actual experimental measurements. Furthermore, there are often multiple aspects to a design such as a specified nucleic acid sequence (e.g., a sequence that encodes an enzyme or transcription factor), the molecular interactions that a designer intends to result from the introduction of this sequence (e.g., chemical modification of metabolites or regulation of gene expression), and the experiments and data associated with the system. All these perspectives need to be connected together to facilitate the engineering of biological systems.

The *Synthetic Biology Open Language* (SBOL) has been developed as a standard to support the specification and exchange of biological design information in synthetic biology, following an open community process involving both "wet" bench scientists and "dry" scientific modelers and software developers, across academia, industry, and other institutions. Previous nucleic acid sequence description formats lack key capabilities relative to SBOL, as shown in Figure 1. Simple sequence encoding formats such as FASTA encode little besides sequence information. More sophisticated formats such as GenBank and Swiss-Prot provide a flat annotation of sequence features that is well suited to describing natural systems but unable to represent the functional relations and multi-layered design structure common to engineered systems. Modeling languages, such as the Systems Biology Markup Language (SBML) Hucka et al. (2003), can be used to represent biological processes, but are not sufficient to represent the associated nucleotide or amino acid sequences. SBOL covers both of these needs, by providing a modular and hierarchical representation of the structure and function of a genetic design, as well as its relationship to and use within experiment plans, data, models, etc.

SBOL uses existing Semantic Web practices and resources, such as *Uniform Resource Identifiers* (`IRI`s) and ontologies, to unambiguously identify and define biological system elements, and to provide serialization formats for encoding this information in electronic data files. The SBOL standard further describes the rules and best practices on how to use this data model and populate it with relevant design details. The definition of the data model, the rules on the addition of data within the format, and the representation of this in electronic data files are intended to make the SBOL standard a useful means of promoting data exchange between laboratories and between software programs.

### Differences from Prior Versions of SBOL

SBOL 1 focused on representing the structural aspects of genetic designs: it allowed the exchange of information about DNA designs and their sequence features, but could not represent molecules other than DNA or the functional aspects of designs. SBOL 2 enabled the description and exchange of hierarchical, modular representations of both the intended structure and function of designed biological systems, as well as providing support for representing provenance, combinatorial designs, genetic design implementations, external file attachments, experimental data, and numerical measurements. SBOL 3.0, defined by this document, condenses and simplifies these prior representations based on experiences in deployment across a variety of scientific and industrial settings.

Specifically, SBOL 3.0 improves on its predecessor SBOL 2.3 by:

- Separating sequence features from part/sub-part relationships.
- Renaming ComponentDefinition/Component to Component/SubComponent.
- Merging Component and Module classes.
- Ensuring consistency between data model and ontology terms.
- Extending the means to define and reference SubComponents.
- Refining requirements on object IRIs.

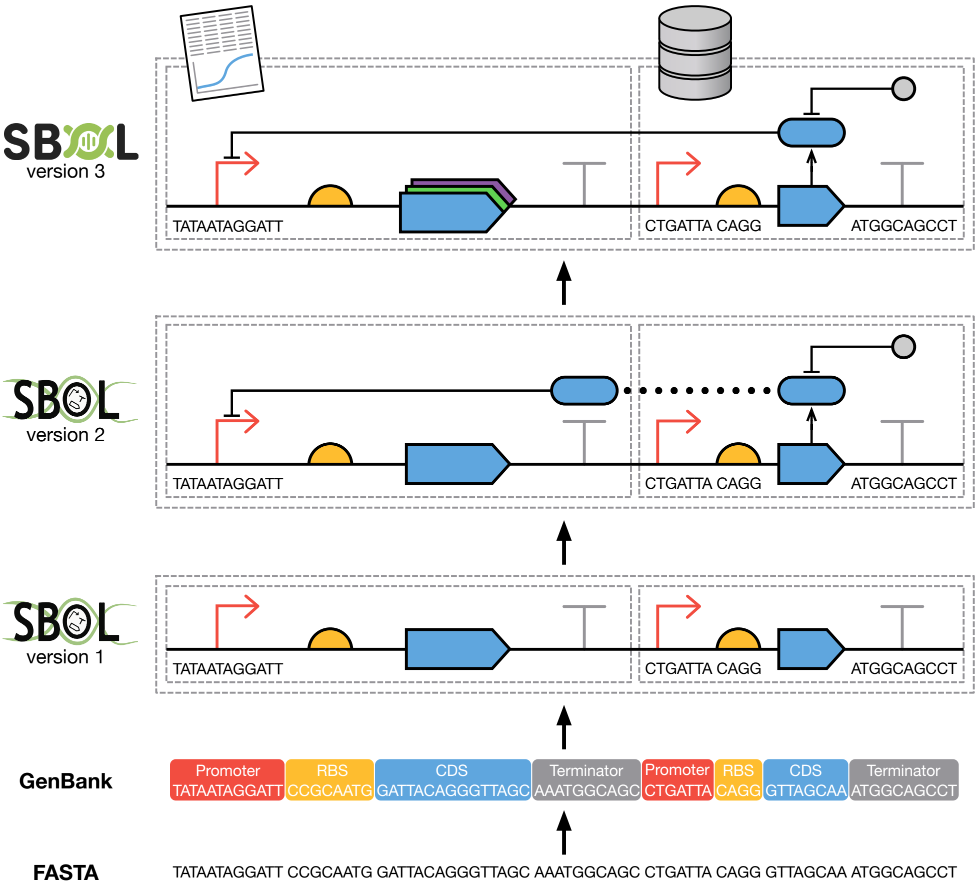

Figure 1: SBOL extends prior sequence description formats to represent both the structure and function of a genetic design in a modular, hierarchical manner, as well as its relationship to, and use within, experiments, plans, data, models, etc.

- Enabling graph-based serialization.
- Moving to Systems Biology Ontology (SBO) for Component types.
- Making all sequence associations explicit.
- Making interfaces explicit.
- Generalizing SequenceConstraints into a general structural Constraint class.
- Expanding the set of allowed sequence constraints.

## 2 A Brief History of SBOL

The SBOL effort was started in 2006 with the goal of developing a data exchange standard for genetic designs. Herbert Sauro (University of Washington) secured a grant from Microsoft in the field of computational synthetic biology, which was used to fund the initial meeting in Seattle on April 26-27, 2008. This workshop was organized by Herbert Sauro, Sean Sleight, and Deepak Chandran, and included talks by Raik Gruenberg, Kim de Mora, John Cumbers, Christopher Anderson, Mac Cowell, Jason Morrison, Jean Peccoud, Ralph Santos, Andrew Milar, Vincent Rouilly, Mike Hucka, Michael Blinov, Lucian Smith, Sarah Richardson, Guillermo Rodrigo, Jonathan Goler, and Michal Galdzicki.

Michal's early efforts were instrumental in making SBOL successful. As part of his doctoral work, he led the development of PoBol (Provisional BioBrick Language), as SBOL was originally known. He organized annual workshops from 2008 to 2011 and kept the idea of developing a genetic design standard alive. The original SBOL 1.0 was developed by a small group of dedicated researchers calling themselves the Synthetic Biology Data Exchange Working Group, meeting at Stanford in 2009 and Anaheim, CA in 2010. During the Anaheim meeting, the community decided to write a letter to Nature Biotechnology highlighting the issue of reproducibility in synthetic biology Peccoud et al. (2011). This letter was initiated by Jean Peccoud and submitted by participants of the Anaheim meeting, including Deepak Chandran, Douglas Densmore, Dmytriv, Michal Galdzicki, Timothy Ham, Cesar Rodriquez, Jean Peccoud, Herbert Sauro, and Guy-Bart Stan. The overall pace of development quickened when several new members joined at the next workshop in Blacksburg, Virginia on January 7-10, 2011. This early work was also supported by an STTR grant from the National Institute of Health (NIH #1R41LM010745 and #9R42HG006737, from 2010-13) in collaboration with Clark & Parsia, LLC (Co-PIs: John Gennari and Evren Sirin). New members included Cesar Rodriguez, Mandy Wilson, Guy-Bart Stan, Chris Myers, and Nicholas Roehner.

The SBOL Developers Group was officially established at a meeting in San Diego in June 2011. Rules of governance were established, and the first SBOL editors were elected: Mike Galdzicki, Cesar Rodriguez, and Mandy Wilson. At our next meeting in Seattle in January 2012, Herbert Sauro was elected the SBOL chair, and two new editors were added: Matthew Pocock and Ernst Oberortner. New developers joining at these workshops included several representatives from industry, Kevin Clancy, Jacob Beal, Aaron Adler, and Fusun Yaman Sirin. New members from Newcastle University included Anil Wipat, Matthew Pocock, and Goksel Misirli.

Development of the first software library (libSBOLj) based on the SBOL standard was initiated by Allan Kuchinsky, a research scientist from Agilent, at the 2011 meeting. By the time of the 2012 meeting, the first data exchange between software tools using SBOL was conducted when a design was passed from Newcastle University's VirtualParts Repository to Boston University's Eugene tool, and finally to University of Utah's iBioSim tool.

SBOL 1.0 was officially released in October 2011. In March 2012, SBOL 1.1 was released, the version that this document replaces. SBOL 1.1 did not make any major changes, but provided a number of small adjustments and clarifications, particularly around the annotation of sequences. Multi-institutional data exchange using SBOL 1.1 was later demonstrated in Nature Biotechnology Galdzicki et al. (2014).

While SBOL 1.1 had a number of significant advantages over the GenBank representation of DNA sequences, such as representing hierarchical organization of DNA components, it was still limited in other respects. The major topic of discussion at the 8th SBOL Workshop at Boston University in November 2012 was how to address these shortcomings through extensions. Several extensions were discussed at this meeting, such as a means to describe genetic regulation, which later became important classes in the 2.x specification.

A general framework for SBOL 2.0 emerged at the 9th SBOL workshop at Newcastle University in April 2013. Subsequently, Nicholas Roehner, Matthew Pocock, and Ernst Oberortner drafted a proposal for SBOL 2.0, and Nicholas presented this proposal at the SEED conference in Los Angeles in July 2014 Roehner et al. (2015). The proposed 2.0 data model was discussed over the course of the 10th, 11th, and 12th workshops. The SBOL 2.0 specification document was drafted at the 13th workshop in Wittenberg, Germany. The SBOL 2.x data model presented was essentially the result of these meetings and ongoing discussions conducted through the SBOL Developers mailing lists, plus minor adjustments and updates approved by the community through subsequence meetings and mailing list discussions.

From 2014 to 2019, development of SBOL 2.x was funded in large part by a grant from the National Science Foundation (DBI-1355909 and DBI-1356041). The SBOL 2.x specification documents and the supporting software libraries are due in no small part to this support. Any opinions, findings, and conclusions or recommendations expressed in SBOL materials are those of the author(s) and do not necessarily reflect the views of the National Science Foundation.

The Computational Modeling in Biology Network (COMBINE) holds regular workshops at which synthetic biologists and systems biologists work toward a common goal of integrating biological knowledge through interoperable and non-overlapping data standards. Several SBOL Developers proposed that SBOL join this larger standards community after attended a COMBINE workshop in April 2014. The proposal passed and SBOL workshops have been co-located with COMBINE meetings since the 11th workshop at the University of Southern California in August 2014.

In 2019 the SBOL Industrial Consortium was established as a pre-competitive non-profit organization supporting innovation, dissemination, and integration of SBOL standards, tools and practices for practical applications in an industrial environment. The SBOL Industrial Consortium meets regularly to coordinate its activities, and organises an Industrial Advisory Board to give an industrial perspective on SBOL, as well as providing financial support for projects, activities, and infrastructure within the SBOL community. Member organizations include Raytheon BBN Technologies, Doulix, Integrated DNA Technologies, Twist Bioscience, Amyris, Inscripta, Teselagen, Shipyard Toolchains, and Zymergen.

Discussions related to SBOL 3 began at the COMBINE meetings and on the mailing list beginning in the summer of 2018. Over the next year and a half, several SBOL Enhancement Proposals (SEPs) were written and discussed. During the early months of 2020, these SEPs were voted on and approved by the SBOL community. The initial version of the SBOL 3 specification was drafted during HARMONY 2020 at the European Bioinformatics Institute (EBI) in Hinxton, United Kingdom in March 2020.

The authors would also like to thank Michael Hucka for developing the LaTeX style file used to develop this document (Hucka, 2017).

## 3 Overview of SBOL

Synthetic biology designs can be described using:

- Structural terms, e.g., a set of annotated sequences or information about the chemical makeup of components.
- Functional terms, e.g., the way that components might interact with each other.

As an example, consider an expression cassette, such as the one found in the plasmid pUC18 Norrander et al. (1983). The system is designed to visually indicate whether a gene has been inserted into the plasmid: in the presence of IPTG, it expresses an enzyme that hydrolyses X-gal to form a blue product, but successful insertion disrupts the expression cassette and prevents the formation of this product. Internally, it has a number of parts, including a promoter, the lac repressor binding site, and the lacZ coding sequence. These parts have specific component-level interactions with IPTG and X-gal, as well as native host gene products, transcriptional machinery, and translational machinery that collectively cause the desired system-level behavior.

In SBOL 3, both the structural and functional aspects are described using a class called `Component`, as depicted in Figure 2. Namely, to represent structural aspects, a `Component` can include `Feature`s, some of which may be at some `Location` within a `Sequence`. A `Component` can also include `Constraint`s between these features. To represent functional aspects, a `Component` can include `Interaction`s that can refer to relationships between participating `Feature`s. Finally, a `Component` can have its behavior described using a `Model`.

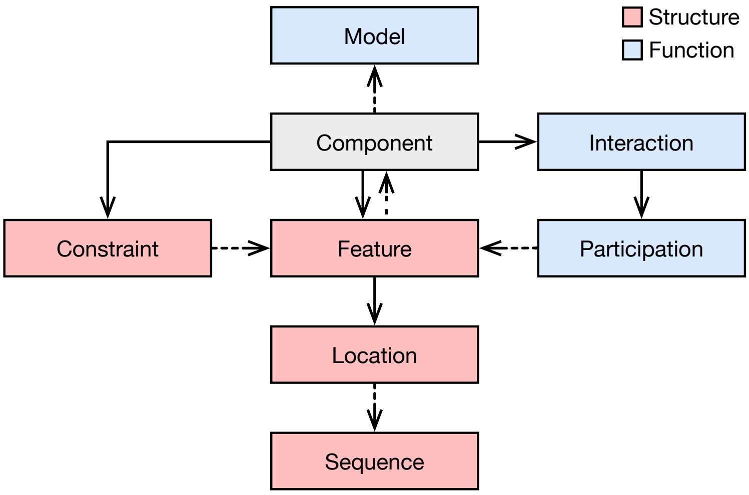

Figure 2: The SBOL `Component` object and related objects. Solid arrows indicates ownership, whereas a dashed arrow represents a reference to an object of another class. Red boxes represent structural objects, while blue boxes represent functional objects. To represent structural aspects, a `Component` can include `Feature`s, which may refer to `Location`s within a `Sequence`. A `Component` can also include `Constraint`s between these features. To represent functional aspects, a `Component` can include `Interaction`s that can refer to relationships between participating `Feature`s. Finally, a `Component` can have its behavior described using a `Model`.

To continue with the pUC18 example, the description would begin with a top-level `Component` that represents the entire system. This `Component` specifies the structural elements that make up the cassette by referencing a number of `SubComponent` objects. These would include the DNA `SubComponent` for the promoter and the simple chemical `SubComponent` for IPTG, for example. The `Component` objects can be organized hierarchically. For example, the plasmid `Component` might reference `SubComponent`s for the promoter, coding sequence, etc. Each `Component` object can also include the actual Sequence information (if available), as well as SubComponent objects that identify the Locations of the promoters, coding sequences, etc., on the Sequence. In order to specify functional information, the Component can also specify Interaction objects that describe any qualitative relationships among SubComponent Participations, such as how IPTG and X-gal interact with the gene products. Finally, a Component object can point to a Model object that provides a reference to a complete computational model expressed in a language such as SBML Hucka et al. (2003), CellML Cuellar et al. (2003), or MATLAB MathWorks (2015).

Whereas Figure 2 provides an overview of the classes used for describing designs within the SBOL 3 data model, Figure 3 shows the rest of the classes used to describe the usage of a design within design-build-test-learn workflows in general. In particular, designs can be expressed using CombinatorialDerivations, Components, and Sequences. These can describe not only genetic designs, but also designs for strains, multicellular systems, media, samples, etc. A CombinatorialDerivation allows one to specify a design pattern where individual SubComponents can be selected from a set of variants. The Implementation class is the build class, and it is used to represent physical artifacts like an actual sample of a plasmid. The Experiment and ExperimentalData classes are the test classes, allowing description of a collection of data generated in an experiment. The Model class, discussed earlier, associates learned information with a design. The prov:Activity class is taken from the provenance ontology (PROV-O), which is described in Section A.1. For example, a build prov:Activity describes how an Implementation is constructed using a Component description. On the other hand, a test prov:Activity describes how an Experiment is conducted using an Implementation artifact. The Collection class has members, which can be of any of these types or Collections themselves. Finally, all of these objects can refer to objects of the Attachment class, which are used to link out to external data (images, spreadsheets, textual documents, etc.). The next sections provide complete definitions and details for all of these classes.

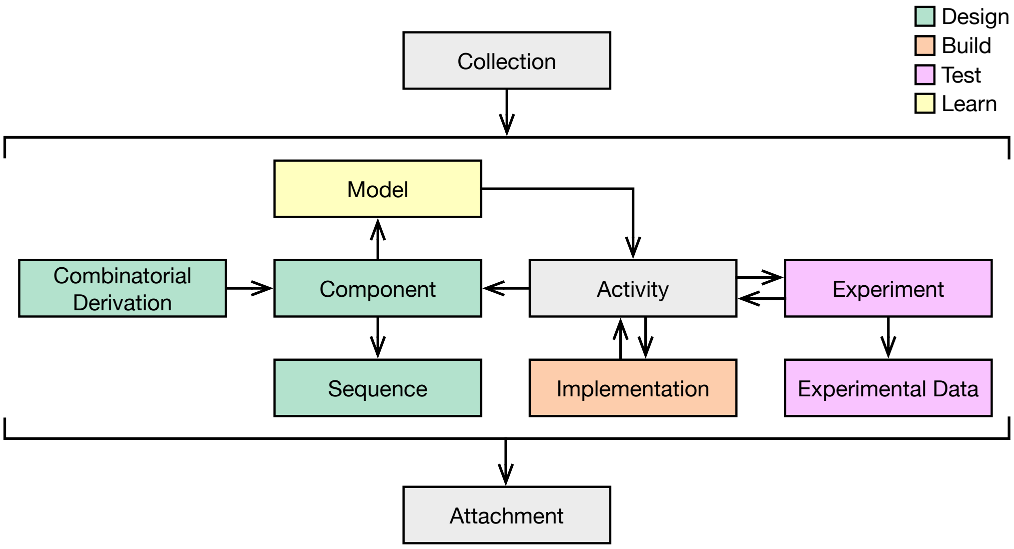

Figure 3: Main classes of information represented by the SBOL 3 standard, and their relationships. Green boxes represent design classes, orange boxes represent build classes, purple boxes represent test classes, yellow boxes represent learn classes, and the gray boxes represent additional utility classes. Each of these classes will be described in more detail below.

## 4 Conventions

This section provides some preliminary information to aid in the understanding of the specification. The SBOL data model is specified using Unified Modeling Language (UML) 2.0 diagrams (OMG 2005). This section reviews terminology conventions, the basics of UML diagrams, and our naming conventions.

### 4.1 Terminology Conventions

This document indicates requirement levels using the controlled vocabulary specified in IETF RFC 2119. In particular, the key words "MUST", "MUST NOT", "REQUIRED", "SHALL", "SHALL NOT", "SHOULD", "SHOULD NOT", "RECOMMENDED", "MAY", and "OPTIONAL" in this document are to be interpreted as described in RFC 2119.

- The words "MUST", "REQUIRED", or "SHALL" mean that the item is an absolute requirement.
- The phrases "MUST NOT" or "SHALL NOT" mean that the item is an absolute prohibition.
- The word "SHOULD" or the adjective "RECOMMENDED" mean that there might exist valid reasons in particular circumstances to ignore a particular item, but the full implications need to be understood and carefully weighed before choosing a different course.
- The phrases "SHOULD NOT" or "NOT RECOMMENDED" mean that there might exist valid reasons in particular circumstances when the particular behavior is acceptable or even useful, but the full implications need to be understood and the case carefully weighed before implementing any behavior described with this label.
- The word "MAY" or the adjective "OPTIONAL" mean that an item is truly optional.

### 4.2 UML Diagram Conventions

The types of biological design data modeled by SBOL are commonly referred to as *classes*, especially when discussing the details of software implementation. Each SBOL class can be instantiated by many SBOL objects. These objects MAY contain data that differ in content, but they MUST agree on the type and form of their data as dictated by their common class. Classes are represented in UML diagrams as rectangles labeled at the top with class names (see Figure 4 for examples).

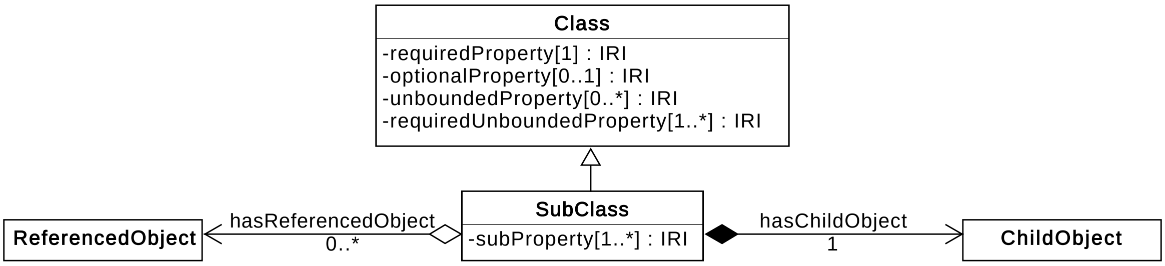

Figure 4: Examples of UML diagram conventions used in this document

Classes can be connected to other classes by association properties, which are represented in UML diagrams as arrows. These arrows are labeled with data cardinalities in order to indicate how many values a given association property can possess (see below). The remaining (non-association) properties of a class are listed below its name. Each of the latter properties is labeled with its data type and cardinality.

In the case of an association property, the class from which the arrow originates is the owner of the association property. A diamond at the origin of the arrow indicates the type of association. Open-faced diamonds indicate shared aggregation, also known as a reference, in which the owner of the association property exists independently of its value.

By contrast, filled diamonds indicate composite aggregation, also known as a part-whole relationship, in which the value of the association property MUST NOT exist independently of its owner. In addition, in the SBOL data model, it is REQUIRED that the value of each composite aggregation property is a unique SBOL object (that is, not the value for more than one such property). Note that in all cases, composite aggregation is used in such a way that there SHOULD NOT be duplication of such objects. Such objects are also commonly referred to as "child" objects, and their owning objects as "parent" objects.

All SBOL properties are labeled with one of several restrictions on data cardinality. These are defined, per RDF, as:

- 1 - EXACTLY ONE: the property is REQUIRED, and there MUST be exactly one value for this property.
- 0...1 - ZERO OR ONE: the property is optional, such that there MAY be a single value for this property, or it MAY be absent.
- 0...∗ - ZERO OR MORE: the property is unbounded, such that there MAY be any number of values for this property, including none.
- 1...∗ - ONE OR MORE: the property is REQUIRED, such that there MAY be any number of values for this property, as long as there is at least one.

Finally, classes can inherit the properties of other classes. Inheritance relationships are represented in UML diagrams as open-faced, triangular arrows that point from the inheriting class to the inherited class. Some classes in the SBOL data model cannot be instantiated as objects and exist only to group common properties for inheritance. These classes are known as abstract classes and are noted as such in their descriptions.

### 4.3 Naming and Typographic Conventions

SBOL classes are named using upper "camel case," meaning that each word is capitalized and all words are run together without spaces, e.g., `Identified`, `SequenceFeature`. Properties, on the other hand, are named using lower camel case, meaning that they begin lowercase (e.g., `role`) but if they consist of multiple words, all words after the first begin with an uppercase letter (e.g., `roleIntegration`).

SBOL properties are always given singular names irrespective of their cardinality, e.g., `role` is used rather than `roles` even though a component can have multiple roles. This is because each relation can potentially stand on its own, irrespective of the existence of others in the set.

Two conventions are used for property names, `name` and `hasName`. When a property is pointing to a class using the same name, it uses the `hasName` convention (e.g., the `Component` class uses `hasFeature` to point to a `Feature` object). When the property uses a different name than the class of the object it points to, it uses the `name` convention instead (e.g., the `Constraint` class uses `subject` to point to a `Feature` object).

## 5 Identifiers and Types

### 5.1 Internationalized Resource Identifiers

As SBOL is built upon the Resource Description Framework (RDF), all class instances are identified by an Internationalized Resource Identifier (IRI), such as a URL or UUID. In the SBOL data model, the value of an association property MUST be a `IRI` or set of `IRI`s that refer to SBOL objects belonging to the class at the tip of the arrow. Every `Identified` object's IRI MUST be globally unique among all other `Identified` object IRIs. It is also highly RECOMMENDED that the `IRI` structure follows the recommended best practices for compliant `IRI`s specified in Section 7.2.

Whenever a `TopLevel` object's URI is a URL (e.g., following the conventions of HTTP(S) rather than a UUID), its structure MUST comply with the following rules:

- A `TopLevel` URL MUST use the following pattern: `[namespace]/[local]/[displayId]`, where `namespace` and `displayId` are required fragments, and the `local` fragment is an optional relative path.

For example, a `Component` might have the URL `https://synbiohub.org/public/igem/BBa_J23070`, where `namespace` is `https://synbiohub.org`, `local` is `public/igem`, and `displayId` is `BBa_J23070`.

- A `TopLevel` object's URL MUST NOT be included as prefix for any other `TopLevel` object.

For example, the `BBa_J23070_seq` `Sequence` object cannot have a URL of `https://synbiohub.org/public/igem/BBa_J23070/BBa_J23070_seq`, since the `https://synbiohub.org/public/igem/BBa_J23070` prefix is already used as a URL for the `BBa_J23070` `Component` object.

- The URL of any child or nested object MUST use the following pattern: `[parent]/[displayId]`, where `parent` is the URL of its parent object. Multiple layers of child objects are allowed using the same `[parent]/[displayId]` pattern recursively.

For example, a `SequenceFeature` object owned by the `BBa_J23070` `Component` and having a `displayId` of `SequenceFeature1` will have a URL of `https://synbiohub.org/public/igem/BBa_J23070/SequenceFeature1`. Similarly, if the `SequenceFeature1` object has a `Location` child object with a `displayId` of `Location1`, then that object will have the URL `https://synbiohub.org/public/igem/BBa_J23070/SequenceFeature1/Location1`.

### 5.2 SBOL URLs

The SBOL namespace, which is `http://sbols.org/v3#`, is used to indicate which entities and properties in an SBOL document are defined by SBOL. For example, the URL of the type `Component` is `http://sbols.org/v3#Component`. This convention is assumed throughout the specification. The SBOL namespace MUST NOT be used for any entities or properties not defined in this specification.

Other namespaces are also used by SBOL, however. Where possible, we have re-used predicates from widely-used terminologies (such as Dublin Core DCMI Usage Board (2012)) to expose as much of the data as practical to such standard RDF tooling. Similarly, existing biological ontologies are used where applicable for specifying types, roles, etc. Likewise, Section Section A details complementary standards that are RECOMMENDED for use in combination with SBOL.

### 5.3 Primitive Data Types

When SBOL uses simple "primitive" data types such as `String`s or `Integer`s, these are defined as the following specific formal types:

- `String`: http://www.w3.org/2001/XMLSchema#string

*Example: "LacI coding sequence"*

- `Integer`: http://www.w3.org/2001/XMLSchema#integer
  *Example: 3*
- `Long`: http://www.w3.org/2001/XMLSchema#long
  *Example: 9223372036854775806*
- `Double`: http://www.w3.org/2001/XMLSchema#double
  *Example: 3.14159*
- `Boolean`: http://www.w3.org/2001/XMLSchema#boolean
  *Example:* `true`

The term `literal` is used to denote an object that can be any of the five types listed above.

In addition to the simple types listed above, SBOL also uses objects with types *Internationalized Resource Identifier* (`IRI`). It is important to realize that in RDF, a `IRI` might or might not be a resolvable URL (web address). A `IRI` is always a globally unique identifier within a structured namespace. In some cases, that name is also a reference to (or within) a document, and in some cases that document can also be retrieved (e.g., using a web browser).

### 5.4 SBOL Types

All SBOL objects are given the most specific `rdfType` in the SBOL 3 namespace ("`http://sbols.org/v3#`") that defines the type of the object. Likewise, properties in the SBOL 3 namespace should only be used by objects with an SBOL 3 `rdfType`.

SBOL does not use multiple inheritance: all SBOL classes are disjoint except with respect to their abstract parent classes. Accordingly, an object MUST NOT be given two `rdfType` properties referring to disjoint classes in the SBOL 3 namespace. An object MAY have redundant `rdfType` properties for its parent types, but this is NOT RECOMMENDED.

For example, an object cannot have both the `rdfType` of `Collection` and `Component`. A `Component` could also have an `rdfType` for `TopLevel` and `Identified`, but this is discouraged.

### 5.5 Object Closure and Document Composition

In RDF, there is no requirement that all of the information about an object be stored in one location. Instead, there is a "open world" assumption that additional triples describing the object may be acquired at any time. Documents are allowed to be fragmented and composed in an arbitrary manner, down to their underlying atomic triples, with no consideration for object structure.

This limits the ability to reason about properties of objects and validate the correctness of a model. For example, it would not be possible to validate that an `Identified` object has no more than one value for its `displayId` property, because it would not be possible to determine whether some other document somewhere in the world holds a second value for the property.

SBOL addresses this by adding an object closure assumption that allows stronger reasoning about individual objects and their children. For any given SBOL document, if the document contains an `rdfType` statement regarding an `Identified` object $X$, then it is assumed that the document also contains all other property statements about object $X$ as well. This enables strong validation rules, since any statement of the form "$X$ *predicate* $Y$" that is not present can be assumed to be false. For example, if a document has one value for an object's `displayId`, then it is valid to conclude that there are no other `displayId` values, and thus its "zero or one" cardinality requirement is satisfied.

We further assume that any document containing an object also contains all of its child objects. In other words, the fundamental unit of SBOL documents is the `TopLevel` object, and any document containing a `TopLevel` also contains the complete set of information necessary to describe that `TopLevel`—but not necessarily any other TopLevel objects that it refers to. For example, a document containing a Component describing a plasmid is guaranteed to contain every Feature of the plasmid as well as every Interaction and Constraint that relates those features, but the document might not contain the Sequence for the plasmid or the definitions for the Component objects linked from its SubComponent parts.

An SBOL document thus cleaves naturally along the boundaries of TopLevel objects, implying the following set of rules of fragmentation and composition of documents:

- Any subset of TopLevel objects in a valid SBOL document is also a valid SBOL document.
- Any disjoint set of TopLevel objects from different SBOL documents MAY be composed to form a new SBOL document. The result is not guaranteed to be valid, however, since the composition may expose problems due to the relationships between TopLevel objects from different documents.
- If two TopLevel objects in different SBOL documents have the same identity and and both they and their child objects contain equivalent sets of property assertions, then they MAY be treated as identical and freely merged.
- If two TopLevel objects in different SBOL documents have the same identity but different property values, then they MUST be considered different (possibly conflicting) versions, and any merger managed through some version control process.

## 6 SBOL Data Model

The section describes the SBOL data model in detail. Best practices when using the standard can be found in Section 7.

### 6.1 Identified

All SBOL-defined classes are directly or indirectly derived from the `Identified` abstract class. This inheritance means that all SBOL objects are uniquely identified using `IRI`s that uniquely refer to these objects within an SBOL document or at locations on the World Wide Web.

As shown in Figure 5, the `Identified` class includes the following properties: `displayId`, `name`, `description`, `prov:wasDerivedFrom`, and `prov:wasGeneratedBy`.

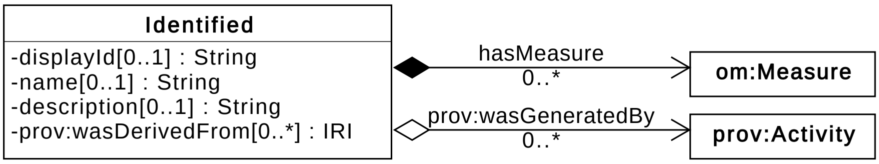

Figure 5: Diagram of the `Identified` abstract class and its associated properties

##### *The `displayId` property*

The `displayId` property is an OPTIONAL identifier with a data type of `String`. This property is intended to be an intermediate between a IRI and the `name` property that is machine-readable, but more human-readable than the full IRI of an object.

If the `displayId` property is used, then its `String` value MUST be composed of only alphanumeric or underscore characters and MUST NOT begin with a digit.

Note that for objects whose IRI is a URL, the requirements on URL structure in Section 5.1 imply that the `displayId` MUST be set.

##### *The `name` property*

The `name` property is OPTIONAL and has a data type of `String`. This property is intended to be displayed to a human when visualizing an `Identified` object.

If an `Identified` object lacks a name, then software tools SHOULD instead display the object's `displayId` or IRI. It is RECOMMENDED that software tools give users the ability to switch perspectives between `name` properties that are human-readable and `displayId` properties that are less human-readable, but are more likely to be unique.

##### *The `description` property*

The `description` property is OPTIONAL and has a data type of `String`. This property is intended to contain a more thorough text description of an `Identified` object.

##### *The `prov:wasDerivedFrom` property*

An `Identified` object MAY have zero or more `prov:wasDerivedFrom` properties, each of type IRI. This property is defined by the PROV-O ontology and is located in the `https://www.w3.org/ns/prov#` namespace (Reference:

Section A.1).

An `Identified` object with this property refers to one or more non-SBOL resources or SBOL `Identified` objects from which this object was derived. An `Identified` object MUST NOT refer to itself via its own `prov:wasDerivedFrom` property or form a cyclical chain of references via its `prov:wasDerivedFrom` property and those of other `Identified` objects. For example, the reference chain "*A* was derived from *B* and *B* was derived from *A*" is cyclical.

##### *The* `prov:wasGeneratedBy` *property*

An `Identified` object MAY have zero or more `prov:wasGeneratedBy` properties, each of type IRI. This property is defined by the PROV-O ontology and is located in the `https://www.w3.org/ns/prov#` namespace (Reference: Section A.1).

An `Identified` object with this property refers to one or more `prov:Activity` objects that describe how this object was generated. Provenance history formed by `prov:wasGeneratedBy` properties of `Identified` objects and entity references in `prov:Usage` objects MUST NOT form circular reference chains.

##### *The* `hasMeasure` *property*

An `Identified` object MAY have zero or more `hasMeasure` properties, each of which refers to a `om:Measure` object that describe measured parameters for this object. `om:Measure` objects are defined by the OM ontology and is located in the `http://www.ontology-of-units-of-measure.org/resource/om-2/` namespace (Reference: Section A.2).

### 6.2 TopLevel

`TopLevel` is an abstract class that is extended by any `Identified` class that can be found at the top level of an SBOL document or file. In other words, `TopLevel` objects are not nested inside any other object via *composite aggregation* (represented by a filled diamond arrowhead on the UML diagrams). Instead of nesting, composite `TopLevel` objects refer to subordinate `TopLevel` objects by their `IRI`s using *shared aggregation* (represented by an open-faced/non-filled diamond arrowhead on the UML diagrams). The `TopLevel` classes defined in this specification are `Sequence`, `Component`, `Model`, `Collection`, `CombinatorialDerivation`, `Implementation`, `Attachment`, `ExperimentalData`, `prov:Activity`, `prov:Agent`, `prov:Plan` (see Figure 6). Each of these classes is described in more detail below, except for the classes from the provenance ontology (PROV-O), which are described in Section A.1.

##### *The hasNamespace property*

A `TopLevel` object MUST have precisely one `hasNamespace` property, which contains a `URL` that defines the namespace portion of URLs for this object and any child objects. If the IRI for the `TopLevel` object is a URL, then the URL of the `hasNamespace` property MUST prefix match that URL.

Note that the requirement for a `hasNamespace` property holds even for objects with IRIs that are not URLs, in order to allow them to be copied into datastores that use URLs. In this case, however, there is no prefix requirement.

##### *The hasAttachment property*

A `TopLevel` object can have zero or more `hasAttachment` properties, each of type IRI specifying an `Attachment` object. The `Attachment` class is described in more detail in Section 6.10.

### 6.3 Sequence

The purpose of the `Sequence` class is to represent the primary structure of a `Component` object and the manner in which it is encoded. This representation is accomplished by means of the `elements` property and `encoding` property (Figure 7).

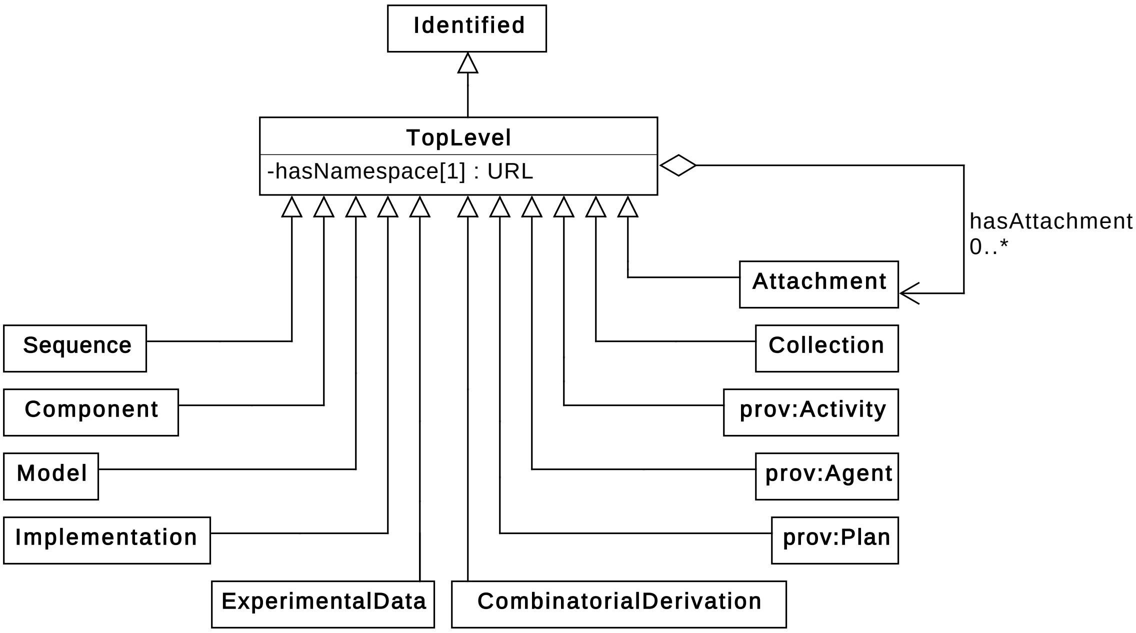

Figure 6: Classes that inherit from the `TopLevel` abstract class.

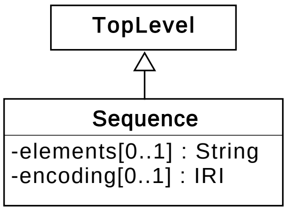

Figure 7: Diagram of the `Sequence` class and its associated properties.

#### *The `elements` property*

The `elements` property is an OPTIONAL `String` of characters that represents the constituents of a biological or chemical molecule. For example, these characters could represent the nucleotide bases of a molecule of DNA, the amino acid residues of a protein, or the atoms and chemical bonds of a small molecule.

If the `elements` property is not set, then it means the particulars of this `Sequence` have not yet been determined.

#### *The `encoding` property*

The `encoding` property has a data type of `IRI`, and is OPTIONAL unless `elements` is set, in which case it is REQUIRED. This property MUST indicate how the `elements` property of a `Sequence` are formed and interpreted. The `encoding` property SHOULD respectively contain a `IRI` identifying from the textual format (`https://identifiers.org/edam:format_2330`) branch of the EDAM ontology.

For example, the `elements` property of a `Sequence` with an IUPAC DNA encoding property MUST contain characters that represent nucleotide bases, such as `a`, `t`, `c`, and `g`. The `elements` property of a `Sequence` with a Simplified Molecular-Input Line-Entry System (SMILES) encoding, on the other hand, MUST contain characters that represent atoms and chemical bonds, such as `C`, `N`, `O`, and `=`.

Table 1 contains a partial list of possible IRI values for the encoding property. These terms are organized by the type of Component (see Table 2) that typically refer to a Sequence with such an encoding. It is RECOMMENDED that the encoding property of a Sequence contains a IRI from Table 1. When the encoding of a Sequence is well described by one of the IRIs in Table 1, it MUST contain that IRI.

| Encoding | URL | Component Type |
|---|---|---|
| IUPAC DNA, RNA | https://identifiers.org/edam:format_1207 | DNA, RNA |
| IUPAC Protein | https://identifiers.org/edam:format_1208 | Protein |
| InChI | https://identifiers.org/edam:format_1197 | Simple Chemical |
| SMILES | https://identifiers.org/edam:format_1196 | Simple Chemical |

Table 1: URLs for specifying the encoding property of a Sequence, organized by the type of Component (see Table 2) that typically refer to a Sequence with such an encoding.

### 6.4 Component

The Component class represents the structural and/or functional entities of a biological design. The primary usage of this class is to represent entities with designed sequences, such as DNA, RNA, and proteins, but it can also be used to represent any other entity that is part of a design, such as simple chemicals, molecular complexes, strains, media, light, and abstract functional groupings of other entities.

As shown in Figure 8, the Component class describes a design entity using the following properties: type, role, hasSequence, hasFeature, hasConstraint, hasInteraction, hasInterface, and hasModel. The hasSequence, hasFeature, and hasConstraint properties are used to represent structural information, while the hasInteraction, hasInterface, and hasModel are used to represent functional information.

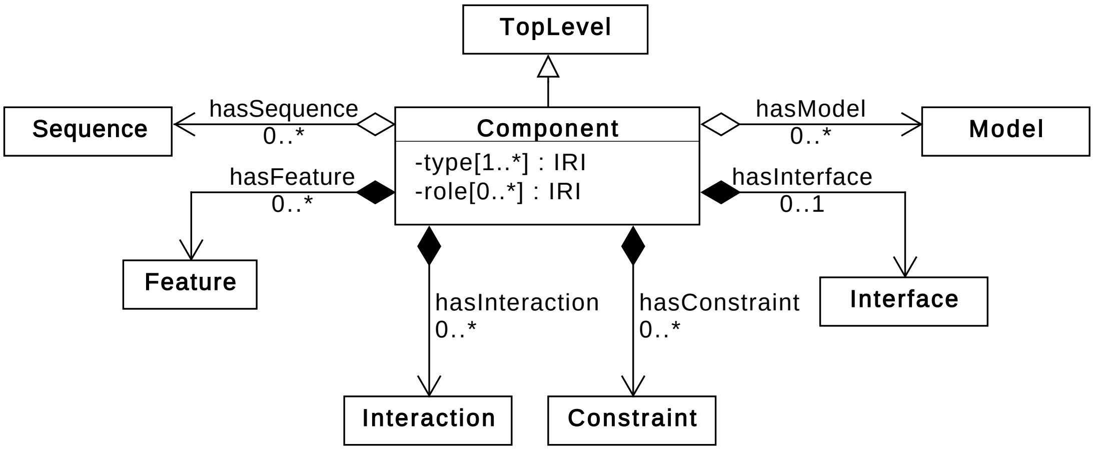

Figure 8: Diagram of the Component class and its associated properties.

***The type property***

A Component MUST have one or more type properties, each of type IRI specifying the category of biochemical or physical entity (for example DNA, protein, or simple chemical) that a Component object abstracts for the purpose of engineering design. For DNA or RNA entities, additional type properties MAY be used to describe nucleic acid topology (circular / linear) and strandedness (double- or single-stranded).

The `type` properties of every `Component` MUST include one or more `IRI`s that MUST identify terms from appropriate ontologies, such as the physical entity representation branch of the Systems Biology Ontology Courtot et al. (2011) or the ontology of Chemical Entities of Biological Interest (ChEBI) Degtyarenko et al. (2008). In order to maximize the compatibility of designs, the `type` property of a `Component` SHOULD contain a `URL` from the physical entity representation branch of the Systems Biology Ontology Courtot et al. (2011). Table 2 provides a partial list of ontology terms and their `URL`s, and any `Component` that can be well-described by one of the terms in Table 2 MUST use the `URL` for that term as a `type`. Finally, if the `type` property contains multiple `IRI`s, then they MUST identify non-conflicting terms (otherwise, it might not be clear how to interpret them). For example, the SBO terms provided by Table 2 would conflict because they specify classes of biochemical entities with different molecular structures.

| Component Type | URL for SBO Term |
|---|---|
| DNA (Deoxyribonucleic acid) | `https://identifiers.org/SBO:0000251` |
| RNA (Ribonucleic acid) | `https://identifiers.org/SBO:0000250` |
| Protein (Polypeptide chain) | `https://identifiers.org/SBO:0000252` |
| Simple Chemical | `https://identifiers.org/SBO:0000247` |
| Non-covalent complex | `https://identifiers.org/SBO:0000253` |
| Functional Entity | `https://identifiers.org/SBO:0000241` |

Table 2: Partial list of the most common SBO terms to specify the molecule type using the `type` property of a `Component`. Systems of multiple interacting molecules (e.g., a plasmid expressing a protein) should use the functional entity type.

*Nucleic Acid Topology types*
Any `Component` classified as DNA (see Table 2) is RECOMMENDED to encode circular/linear topology information in an additional `type` field. This (topology) `type` field SHOULD specify a URL from the Topology Attribute branch of the Sequence Ontology (SO): this is currently just 'linear' or 'circular' as given in Table 3. Topology information SHOULD be specified for DNA `Component` records with a fully specified sequence, except in three scenarios: if the DNA record does not have sequence information, or if the DNA record has incomplete sequence information, or if topology is genuinely unknown. For any `Component` classified as RNA (see Table 2), a topology type field is OPTIONAL. The default assumption in this case is linear topology. In any case, conflicting topologies MUST NOT be specified.

Any `Component` classified as DNA or RNA MAY also have strand information encoded in an additional (third) type field using a URL from the Strand Attribute branch of the SO (currently there are only two possible terms for single or double-stranded nucleic acids, given in Table 3). In absence of this field, the default strand information assumed for DNA is 'double-stranded' and for RNA is 'single-stranded'.

Any other type of `Component` record (protein, simple chemical, etc.) SHOULD NOT have any type field pointing to SO terms from the topology or strand attribute branches of SO.

Note that a *circular* topology instructs software to interpret the beginning / end position of a given sequence (be it DNA or RNA) as arbitrary, meaning that sequence features MAY be mapped or identified across this junction. *Double stranded* instructs software to apply sequence searches to both strands (i.e., sequence and reverse complement of sequence).

##### *The `role` property*

A `Component` MAY have any number of `role` properties, each of type `IRI`, that MUST identify terms from ontologies that are consistent with the `type` property of the `Component`. For example, the `role` property of a DNA or RNA `Component` could contain URLs identifying terms from the Sequence Ontology (SO). As a best practice, a DNA or RNA `Component` SHOULD contain exactly one `URL` that refers to a term from the sequence feature branch of the

| Nucleic Acid Topology | URL for Nucleic Acid Topology Term in SO |
|---|---|
| linear | http://identifiers.org/SO:0000987 |
| circular | http://identifiers.org/SO:0000988 |
| single-stranded | http://identifiers.org/SO:0000984 |
| double-stranded | http://identifiers.org/SO:0000985 |

Table 3: Sequence Ontology (SO) terms to encode DNA or RNA topology information in the type properties of a Component.

SO. Similarly, the role properties of a protein and simple chemical Component SHOULD respectively contain URLs identifying terms from the MolecularFunction (GO:0003674) branch of the Gene Ontology (GO) and the role (CHEBI:50906) branch of the CHEBI ontology. Table 4 contains a partial list of possible ontology terms for the role properties and their URLs. These terms are organized by the type of Component to which they SHOULD apply (see Table 2). Any Component that can be well-described by one of the terms in Table 4 MUST use the URL for that term as a role.

These IRIs might identify descriptive biological roles, such as "metabolic pathway" and "signaling cascade," but they can also identify identify "logical" roles, such as "inverter" or "AND gate", or other abstract roles for describing the function of design. Interpretation of the meaning of such roles currently depends on the software tools that read and write them.

| Component Role | URL for Ontology Term | Component Type |
|---|---|---|
| Promoter | http://identifiers.org/SO:0000167 | DNA |
| RBS | http://identifiers.org/SO:0000139 | DNA |
| CDS | http://identifiers.org/SO:0000316 | DNA |
| Terminator | http://identifiers.org/SO:0000141 | DNA |
| Gene | http://identifiers.org/SO:0000704 | DNA |
| Operator | http://identifiers.org/SO:0000057 | DNA |
| Engineered Region | http://identifiers.org/SO:0000804 | DNA |
| mRNA | http://identifiers.org/SO:0000234 | RNA |
| Effector | http://identifiers.org/CHEBI:35224 | Small Molecule |
| Transcription Factor | http://identifiers.org/GO:0003700 | Protein |

Table 4: Partial list of ontology terms to specify the role property of a Component, organized by the type of Component to which they are intended to apply (see Table 2).

##### *The hasSequence property*

A Component MAY have any number of hasSequence properties, each of type IRI, that MUST reference a Sequence object (see Section 6.3). These objects define the primary structure or structures of the Component.

If a Feature of a Component refers to a Location, and this Location refers to a Sequence, then the Component MUST also include a hasSequence property that refers to this Sequence.

Many Component objects will have exactly one hasSequence property that refers to a Sequence object. In this case, if its has a type from Table 2 and there is an encoding that is cross-listed with this term in Table 1, then the Sequence objects MUST have this encoding (e.g., a Component of type DNA must have a Sequence with an IUPAC DNA encoding). This Sequence is implicitly the entire sequence for this Component (In other words, it is equivalent to a SequenceFeature with an EntireSequence Location that refers to this Sequence).

##### *The `hasFeature` property*

A `Component` MAY have any number of `hasFeature` properties, each of type `IRI` that MUST reference a `Feature` object (see Section 6.4.1). The set of relations between `Feature` and `Component` objects MUST be strictly acyclic.

Taking the `Component` class as analogous to a blueprint or specification sheet for a biological part or a system of interacting biological elements, the `Feature` class represents the specific occurrence of a part, subsystem, or other notable aspect within that design. This mechanism also allows a biological design to include multiple instances of a particular part (defined by reference to the same `Component`). For example, the `Component` of a polycistronic gene could contain two `SubComponent` objects that refer to the same `Component` of a CDS. As another example, consider the `Component` for a network of two-input repressor devices in which the particular repressors have not yet been chosen. This `Component` could contain multiple `SubComponent` objects that refer to the same `Component` of an abstract two-input repressor device.

The `hasFeature` properties of `Component` objects can be used to construct a hierarchy of `SubComponent` and `Component` objects. If a `Component` in such a hierarchy refers to a `Location` object, and there exists a `Component` object lower in the hierarchy that refers to a `Location` object that refers to the same `Sequence` with the same `encoding`, then the `elements` properties of these `Sequence` objects SHOULD be consistent with each other, such that well-defined mappings exist from the "lower level" `elements` to the "higher level" `elements` in accordance with their shared `encoding` properties. This mapping is also subject to any restrictions on the positions of the `Feature` objects in the hierarchy that are imposed by the `SubComponent`, `SequenceFeature`, or `Constraint` objects contained by the `Component` objects in the hierarchy.

For example, in a plasmid `Component` with a promoter `SubComponent`, the sequence at the promoter's `Location` within the plasmid should be the sequence for the promoter. More concretely, consider DNA `Component` that refers to a `Sequence` with an `IUPAC DNA` `encoding` and an `elements` `String` of "`gattaca`." In turn, this `Component` could contain a `SubComponent` that refers to a "lower level" `Component` that also refers to a `Sequence` with an `IUPAC DNA` `encoding`. Consequently, a consistent `elements` `String` of this "lower level" `Sequence` could be "`gatta`," or perhaps "`tgta`" if the `SubComponent` is positioned by a `Location` with an `orientation` of "reverse complement" (see Section 6.4.2).

##### *The `hasConstraint` property*

A `Component` MAY have any number of `hasConstraint` properties, each of type `IRI`, that MUST reference a `Constraint` object (see Section 6.4.3). These objects describe, among other things, any restrictions on the relative, sequence-based positions and/or orientations of the `Feature` objects contained by the `Component`, as well as spatial relations such as containment and identity relations. For example, the `Component` of a gene might specify that the position of its promoter `SubComponent` precedes that of its CDS `SubComponent`. This is particularly useful when a `Component` lacks a `Sequence` and therefore cannot specify the precise, sequence-based positions of its `SubComponent` objects using `Location` objects.

##### *The `hasInteraction` property*

A `Component` MAY have any number of `hasInteraction` properties, each of type `IRI`, that MUST reference an `Interaction` object (see Section 6.4.4).

The `Interaction` class provides an abstract, machine-readable representation of behavior within a `Component` (whereas a more detailed model of the system might not be suited to machine reasoning, depending on its implementation). Each `Interaction` contains `Participation` objects that indicate the roles of the `Feature` objects involved in the `Interaction`.

##### *The `hasInterface` property*

A `Component` MAY have zero or one `hasInterface` property of type `IRI` that MUST reference an `Interface` object (see Section 6.4.5).

An `Interface` object indicates the inputs, outputs, and non-directional points of connection to a `Component`.

##### *The* **hasModel** *property*

A `Component` MAY have any number of `hasModel` properties, each of type `IRI`, that MUST reference a `Model` object (see Section 6.8).

`Model` objects are placeholders that link `Component` objects to computational models of any format. A `Component` object can link to more than one `Model` since each might encode system behavior in a different way or at a different level of detail.

#### 6.4.1 Feature

The `Feature` class, as shown in Figure 9 is used to compose `Component` objects into a structural or functional hierarchy. `Feature` is an abstract class; only its child classes are actually instantiated.

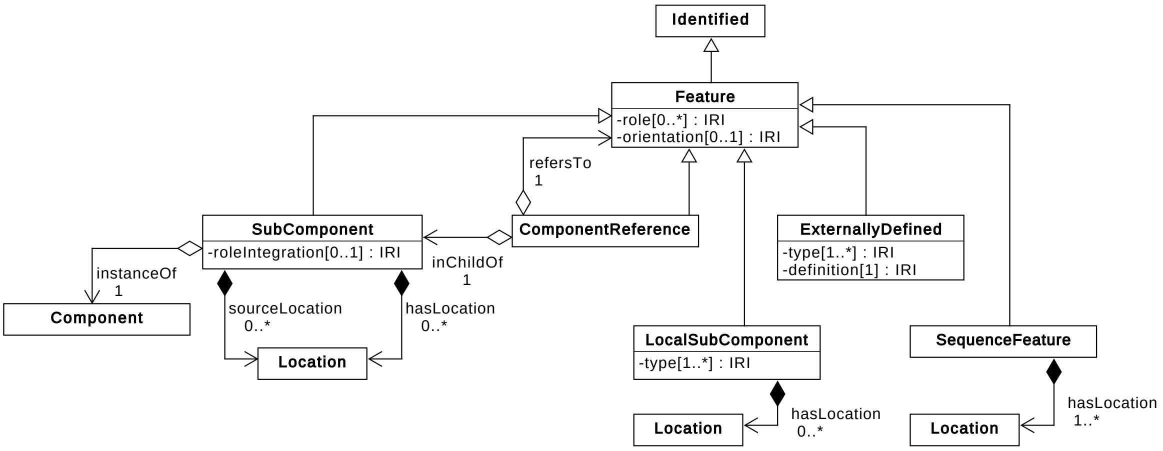

Figure 9: Diagram of the `Feature` class, its children, and associated properties.

##### *The* **role** *property*

Each `Feature` can have zero or more `role` property `IRI`s describing the purpose or potential function of this `Feature` in the *context* of its parent `Component`. If the `role` for a `SubComponent` is left unspecified, then the `role` is determined by the `role` property of the `Component` that it is an `instanceOf`. If provided, these `role` property `IRI`s MUST identify terms from appropriate ontologies. Roles are not restricted to describing biological function; they may annotate a `Feature`'s function in any domain for which an ontology exists. A table of recommended ontology terms for `role` is given in Table 4.

It is RECOMMENDED that these `role` property `IRI`s identify terms that are compatible with the `type` properties of the `Feature`'s parent `Component`. For example, a `role` of a `Feature` which belongs to a `Component` of type DNA might refer to terms from the Sequence Ontology. Likewise, for any feature that is a `SubComponent`, the `role` SHOULD be compatible with the `type` of the `Component` that it links to through its `instanceOf` property.

##### *The* **orientation** *property*

The `orientation` property is OPTIONAL and has a data type of `IRI`. This can be used to indicate how any associated double-stranded `Feature` is oriented on the `elements` of a `Sequence` from their parent `Component`. If a `Feature` object has an `orientation`, then it is RECOMMENDED that it come from Table 5; for reasons of backwards compatability it MAY instead come from Table 6.

| Orientation URL | Description |
|---|---|
| https://identifiers.org/SO:0001030 | The region specified by this Feature or Location is on the elements of a Sequence. |
| https://identifiers.org/SO:0001031 | The region specified by this Feature or Location is on the reverse-complement mapping of the elements of a Sequence. The exact nature of this mapping depends on the encoding of the Sequence. |

Table 5: RECOMMENDED URLs for the orientation property

| Orientation URL | Description |
|---|---|
| http://sbols.org/v3#inline | The region specified by this Feature or Location is on the elements of a Sequence. |
| http://sbols.org/v3#reverseComplement | The region specified by this Feature or Location is on the reverse-complement mapping of the elements of a Sequence. The exact nature of this mapping depends on the encoding of the Sequence. |

Table 6: Permitted alternative URLs for the orientation property. The URLs listed in Table 5 are preferred and SHOULD be used instead where possible.

##### *6.4.1.1 SubComponent*

The SubComponent class is a subclass of the Feature class that can be used to specify structural hierarchy. For example, the Component of a gene might contain four SubComponent objects: a promoter, RBS, CDS, and terminator, each linked to a Component that provides the complete definition. In turn, the Component of the promoter SubComponent might itself contain SubComponent objects defining various operator sites, etc.

***The roleIntegration property***

A roleIntegration specifies the relationship between a SubComponent instance's own set of role properties and the set of role properties on the included Component.

The roleIntegration property has a data type of IRI. A SubComponent instance with zero role properties MAY OPTIONALLY specify a roleIntegration. A SubComponent instance with one or more role properties MUST specify a roleIntegration from Table 7. If zero SubComponent role properties are given and no SubComponent roleIntegration is given, then http://sbols.org/v3#mergeRoles is assumed. It is RECOMMENDED to specify SubComponent role values only if the result would differ from the role values belonging to this SubComponent's included Component.

| roleIntegration URL | Description |
|---|---|
| http://sbols.org/v3#overrideRoles | In the context of this SubComponent, ignore any role given for the included Component. Instead use only the set of zero or more role properties given for this SubComponent. |
| http://sbols.org/v3#mergeRoles | Use the union of the two sets: both the set of zero or more role properties given for this SubComponent as well as the set of zero or more role properties given for the included Component. |

Table 7: Each roleIntegration mode is associated with a rule governing how a SubComponent's role values are to be combined with the included Component's role values.

##### *The `instanceOf` property*

The `instanceOf` property is a REQUIRED `IRI` that refers to the `Component` providing the definition for this `SubComponent`. Among other things, as described in the previous section, this `Component` effectively provides information about the `type` and `role` of the `SubComponent`.

The `instanceOf` property MUST NOT refer to the same `Component` as the one that contains the `SubComponent`. Furthermore, `SubComponent` objects MUST NOT form a cyclical chain of references via their `instanceOf` properties and the `Component` objects that contain them. For example, consider the `SubComponent` objects $A$ and $B$ and the `Component` objects $X$ and $Y$. The reference chain "$X$ has feature $A$, $A$ is an instance of $Y$, $Y$ has feature $B$, and $B$ is an instance of $X$" is cyclical.

##### *The `hasLocation` property*

A `SubComponent` MAY have any number of `hasLocation` properties, each of type `IRI`, that MUST refer to `Location` objects that indicates the location of the `Sequence` from the `instanceOf` `Component` in a `Sequence` of the parent `Component`.

If any `hasLocation` is defined, then there MUST BE precisely one `Sequence` in the `instanceOf` `Component`, as otherwise this relationship is ill-defined.

If no `hasLocation` is defined, this indicates a part / sub-part relationship for which sequence details have not (yet) been determined or involving types for which sequence relationships are not relevant (e.g., inclusion of a reaction chain within a larger metabolic network).

Allowing multiple `Location` objects on a single `SubComponent` is intended to enable representation of discontinuous regions (for example, a coding sequence encoded across a set of exons with interspersed introns). As such, the `Location` objects of a single `SubComponent` MUST NOT specify overlapping regions, since it is not clear what this would mean. There is no such concern with different objects, however, which can freely overlap in `Location` (for example, specifying overlapping linkers for sequence assembly).

##### *The `sourceLocation` property*

The `sourceLocation` property allows for only a portion of a `Component`'s `Sequence` to be included, rather than its entirety. For example, when composing parts with certain assembly methods, some bases on the boundary may be removed or replaced. Another example is describing a deletion or replacement of a portion of a sequence.

A `SubComponent` MAY have any number of `sourceLocation` properties, each of type `IRI`, that MUST refer to `Location` objects that indicate which `elements` of the `instanceOf` `Component`'s `Sequence` are used in defining the parent of the `SubComponent`.

If there are no `sourceLocation` properties, then the whole `Sequence` is assumed to be included.

#### 6.4.1.2 ComponentReference

The `ComponentReference` class is a subclass of `Feature` that can be used to reference `Feature`s within `SubComponent`s.

##### *The `inChildOf` property*

The `inChildOf` property is a REQUIRED `IRI` that refers to a `SubComponent`. The `inChildOf` property MUST refer to a `SubComponent` pointed directly to by the parent of the `ComponentReference`. Specifically:

- If the parent of the `ComponentReference` is a `Component`, then `inChildOf` MUST be one of its `SubComponent`s.
- If the parent of the `ComponentReference` is another `ComponentReference`, then `inChildOf` MUST be a `SubComponent` of the `Component` linked as `instanceOf` the parent's `inChildOf` `SubComponent`.

##### *The `refersTo` property*

The `refersTo` property is a REQUIRED `IRI` that refers to a `Feature`.

This can be used to either link to the `Feature` being referenced or to chain hierarchically through additional layers of `SubComponent`.

- If the `Feature` is a `ComponentReference`, then that `ComponentReference` acts as a hierarchical link in a chain of references, and MUST be either a child of the `ComponentReference` linking to it via `refersTo` or a child of the `Component` linked as `instanceOf` the `ComponentReference`'s `inChildOf` `SubComponent`.
- Otherwise, if the `refersTo` refers to any other type of `Feature`, that `Feature` MUST be a child of the `Component` linked as `instanceOf` the `ComponentReference`'s `inChildOf` `SubComponent`.

For example, `ComponentReference` R1 looking into a `SubComponent` for a plasmid might link with `refersTo` to its own child `ComponentReference` R2, which in turn looks within the `Component` defining the plasmid to the plasmid's CDS `SubComponent`, in turn using `refersTo` to reference a `SequenceFeature` within the `Component` that defines that CDS.

#### 6.4.1.3 LocalSubComponent

The `LocalSubComponent` class is a subclass of `Feature`. This class serves as a way to create a placeholder in more complex `Component`s, such as a variable to be filled in later or a composite that exists only within the context of the parent `Component`.

##### *The `type` property*

The `type` property is REQUIRED and contains one or more `IRI`s. The `type` property is identical to its use in `Component`.

##### *The `hasLocation` property*

A `LocalSubComponent` MAY have any number of `hasLocation` properties, each of type `IRI`, that MUST refer to `Location` objects. These follow the same restrictions as for the `hasLocation` of a `SubComponent`, notably that the `Location`s of `hasLocation` properties attached to the same `LocalSubComponent` MUST NOT overlap.

#### 6.4.1.4 ExternallyDefined

The `ExternallyDefined` class has been introduced so that external definitions in databases like ChEBI or UniProt can be referenced.

##### *The `type` property*

The `type` property is REQUIRED and contains one or more `IRI`s. The `type` property is identical to its use in `Component`.

##### *The `definition` property*

The `definition` property is REQUIRED and is of type `IRI` that links to a canonical definition external to SBOL. When possible, such definitions SHOULD use the recommended external resources in Section 7.6. For example, an `ExternallyDefined` simple chemical might link to ChEBI and a protein might link to UniProt.

#### 6.4.1.5 SequenceFeature

The `SequenceFeature` class describes one or more regions of interest on the `Sequence` objects referred to by its parent `Component`.

##### *The **hasLocation** property*

The hasLocation is REQUIRED and contains one or more IRIs, which MUST refer to Location objects. These follow the same restrictions as for the hasLocation of a SubComponent, notably that the Locations of hasLocation properties attached to the same SequenceFeature MUST NOT overlap.

#### 6.4.2 Location

The Location class (as shown in Figure 10) is used to represent the location of Features within Sequences. This class is extended by the Range, Cut, and EntireSequence classes Location is an abstract class; only its child classes are actually instantiated.

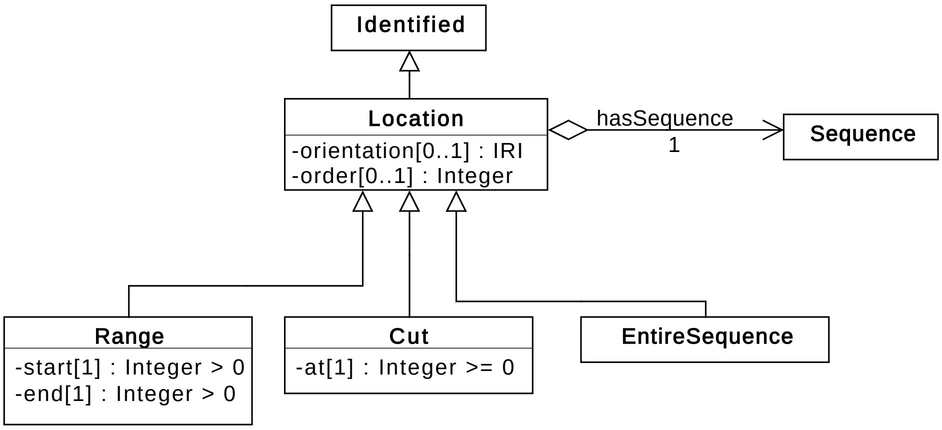

Figure 10: Diagram of the Location class and its associated properties.

##### *The **orientation** property*

The orientation property is OPTIONAL and has a data type of IRI. All subclasses of Location share this property, which can be used to indicate how any associated double-stranded Feature is oriented on the elements of a Sequence from their parent Component. If a Location object has an orientation, then it is RECOMMENDED that it come from Table 5; for reasons of backwards compatability it MAY instead come from Table 6.

As is typical practice in biology, any change in orientation is applied after indices are interpreted. Thus, for example, in a DNA Sequence with elements AAAAACCCCCTTTTTGGGGGTTTTTGGGGG, indices 1-6 with a reverse orientation will select AAAAAC, which would then be reverse complemented to obtain GTTTTT.

##### *The **order** property*

The order property is OPTIONAL and has a data type of Integer. If there are multiple Location objects associated with a Feature, the order property is used to specify the order (in increasing value) in which the specified Locations are to be joined to form the sequence of the Feature. Note that order values MAY be non-sequential and non-positive, if desired.

##### *The **hasSequence** property*

The hasSequence property is REQUIRED and MUST contain the IRI of a Sequence object. All subclasses of Location share this property, which indicates which Sequence object referenced by the containing Component is referenced by the Location.

##### 6.4.2.1 Range

A `Range` object specifies a region via discrete, inclusive `start` and `end` positions that correspond to indices for characters in the `elements` `String` of a `Sequence`.

Note that the index of the first location is 1, as is typical practice in biology, rather than 0, as is typical practice in computer science.

###### *The `start` property*

The `start` property specifies the inclusive starting position of the `Range`. This property is REQUIRED and MUST contain an `Integer` value greater than zero.

###### *The `end` property*

The `end` property specifies the inclusive ending position of the `Range`. This property is REQUIRED and MUST contain an `Integer` value greater than zero. In addition, this `Integer` value MUST be greater than or equal to that of the `start` property.

##### 6.4.2.2 Cut

The `Cut` class has been introduced to enable the specification of a region between two discrete positions. This specification is accomplished using the `at` property, which specifies a discrete position that corresponds to the index of a character in the `elements` `String` of a `Sequence` (except in the case when `at` is equal to zero—see below).

###### *The `at` property*

The `at` property is REQUIRED and MUST contain an `Integer` value greater than or equal to zero. The region specified by the `Cut` is between the position specified by this property and the position that immediately follows it. When the `at` property is equal to zero, the specified region is immediately before the first discrete position or character in the `elements` `String` of a `Sequence`.

##### 6.4.2.3 EntireSequence

The `EntireSequence` class does not have any additional properties. Use of this class indicates that the linked Sequence describes the entirety of the `Component` or `Feature` parent of this Location object.

#### 6.4.3 Constraint

The `Constraint` class can be used to assert restrictions on the relationships of pairs of `Feature` objects contained by the same parent `Component`. Uses of this class include expressing containment (e.g., a plasmid transformed into a chassis strain), identity mappings (e.g., replacing a placeholder value with a complete definition), and expressing relative, sequence-based positions (e.g., the ordering of features within a template). Each `Constraint` includes the `subject`, `object`, and `restriction` properties.

###### *The `subject` property*

The `subject` property is REQUIRED and MUST contain a `IRI` that refers to a `Feature` contained by the same parent `Component` that contains the `Constraint`.

###### *The `object` property*

The `object` property is REQUIRED and MUST contain a `IRI` that refers to a `Feature` contained by the same parent `Component` that contains the `Constraint`. This `Feature` MUST NOT be the same `Feature` that the `Constraint` refers to via its `subject` property.

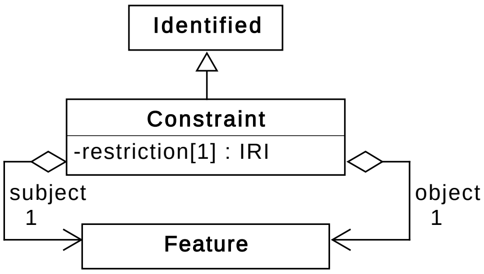

Figure 11: Diagram of the Constraint class and its associated properties.

***The restriction property***

The restriction property is REQUIRED and has a data type of IRI. This property MUST indicate the type of restriction on the locations, orientations, or identities of the subject and object Feature objects in relation to each other. The IRI value of this property SHOULD come from the RECOMMENDED URLs in Table 8, Table 9, and Table 10.

| Restriction URL | Description |
|---|---|
| http://sbols.org/v3#verifyIdentical | The subject and object, after tracing through any layers of ComponentReference, MUST both refer to SubComponent objects with the same instanceOf value or both refer to ExternallyDefined objects with the same definition. *Example: a promoter included via two different subsystems must be the identical.* |
| http://sbols.org/v3#differentFrom | The subject and object, after tracing through any layers of ComponentReference, MUST NOT both refer to SubComponent objects with the same instanceOf value or both refer to ExternallyDefined objects with the same definition. *Example: two fluorescent reporters must be different.* |
| http://sbols.org/v3#replaces | In the context of the parent object of the Constraint, information about the subject should be used in place of all instances of the object. *Example: the J23101 promoter replaces a generic promoter.* |
| http://sbols.org/v3#sameOrientationAs | The subject and object Component objects MUST have the same orientation. *Example: a promoter has the same orientation as the coding sequence it controls.* |
| http://sbols.org/v3#oppositeOrientationAs | The subject and object Component objects MUST have opposite orientations. *Example: a promoter has the opposite orientation as an invertase-activated coding sequence it controls.* |

Table 8: RECOMMENDED URLs for expressing identity and orientation with the restriction property.

#### *6.4.4 Interaction*

The Interaction class (as shown in Figure 12) provides more detailed description of how the Feature objects of a Component are intended to work together. For example, this class can be used to represent different forms of genetic regulation (e.g., transcriptional activation or repression), processes from the central dogma of biology (e.g. transcription and translation), and other basic molecular interactions (e.g., non-covalent binding or enzymatic phosphorylation). Each Interaction includes type properties that refer to descriptive ontology terms and

| Restriction URL | Description |
|---|---|
| http://sbols.org/v3#isDisjointFrom | The subject and object do not overlap in space. *Example: a plasmid is disjoint from a chromosome.* |
| http://sbols.org/v3#strictlyContains | The subject entirely contains the object: they do not share a boundary. *Example: a cell contains a plasmid* |
| http://sbols.org/v3#contains | The subject contains the object and they might or might not share a boundary (i.e., union of strictlyContains, equals, and covers. *Example: a cell contains a protein that may or may not bind to its membrane.* |
| http://sbols.org/v3#equals | The subject and object occupy the same location in space. *Example: a small molecule is distributed throughout an entire sample.* |
| http://sbols.org/v3#meets | The subject and object are connected at a shared boundary. *Example: two strains of adherent cells meet at their membranes.* |
| http://sbols.org/v3#covers | The subject contains the object but also shares a boundary. *Example: a cell covers its transmembrane proteins.* |
| http://sbols.org/v3#overlaps | The subject and object overlap in space, but portions of each are outside of the other. *Example: a transmembrane protein overlaps the cell membrane.* |

Table 9: RECOMMENDED URLs for expressing topological relations with the restriction property.

hasParticipation properties that describe which Feature objects participate in which ways in the Interaction.

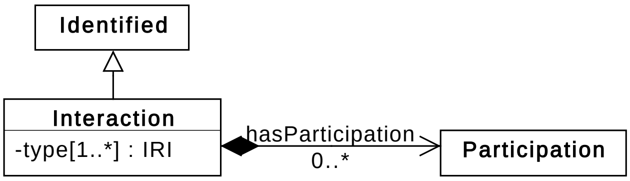

Figure 12: Diagram of the Interaction class and its associated properties.

#### *The type property*

An Interaction is REQUIRED to have one or more type properties, each of type IRI, that describes the behavior represented by an Interaction.

Each type property MUST identify terms from appropriate ontologies. It is RECOMMENDED that exactly one IRI specified by a type property refer to a term from the occurring entity branch of the Systems Biology Ontology (SBO). Table 11 provides a partial list of possible SBO terms for the type property and their corresponding URLs.

If an Interaction is well described by one of the terms from Table 11, then a type property MUST refer to the URL that identifies this term. Lastly, if there are multiple type properties for an Interaction, then they MUST identify non-conflicting terms. For example, the SBO terms “stimulation” and “inhibition” would conflict.

#### *The hasParticipation property*

An Interaction MAY have any number of hasParticipation properties, each of type IRI, that MUST reference a Participation object, each of which identifies the role that its referenced Feature plays in the Interaction.

Even though an Interaction generally contains at least one Participation, the case of zero Participation

| Restriction URL | Description |
|---|---|
| http://sbols.org/v3#precedes | The start of the location for subject is less than the start of the location for object (i.e., union of strictlyPrecedes, meets, and overlaps). *Example: a promoter precedes a ribosome entry site, but the exact boundary between the two will be determined by sequence optimization and assembly planning.* |
| http://sbols.org/v3#strictlyPrecedes | The end of the location for subject is less than the start of the location for object. *Example: a promoter strictly precedes a terminator (with a CDS between them).* |
| http://sbols.org/v3#meets | The end of the location for subject is equal to the start of the location for object. Note: this is a stronger interpretation of meets from Table 9 in the context of a linear sequence. *Example: the 3' region adjacent to a blunt restriction site meets the 5' region adjacent to the site.* |
| http://sbols.org/v3#overlaps | The start of the location for subject is before the start of the location for object and the end of the location for subject is before the end of the location for object. Note: this is a stronger interpretation of overlaps from Table 9 in the context of a linear sequence. *Example: two adjacent oligos overlap in a Gibson assembly plan.* |
| http://sbols.org/v3#contains | The start of the location for subject is less than or equal to the start of the location for object and the end of the location for subject is greater than or equal to the end of the location for object (i.e., union of strictlyContains, equals, finishes, and starts). Note: this is a stronger interpretation of contains from Table 9 in the context of a linear sequence. *Example: a composite part contains a promoter.* |
| http://sbols.org/v3#strictlyContains | The start of the location for subject is before the start of the location for object and the end of the location for subject is after the end of the location for object. Note: this is a stronger interpretation of strictlyContains from Table 9 in the context of a linear sequence. *Example: an RNA transcript strictly contains an intron.* |
| http://sbols.org/v3#equals | The start and end of the location for subject are equal to the start and end of the location for object. Note: this is a stronger interpretation of equals from Table 9 in the context of a linear sequence. *Example: the transcribed region of a CDS part equals the entire part.* |
| http://sbols.org/v3#finishes | The start of the location for subject is after the start of the location for object and the end of the location for subject is equal to the end of the location for object. *Example: a terminator finishes an expression cassette.* |
| http://sbols.org/v3#starts | The start of the location for subject is equal to the start of the location for object and the end of the location for subject is before the end of the location for object. *Example: a promoter starts an expression cassette.* |

Table 10: RECOMMENDED URLs for expressing sequential relations with the restriction property. Note that these relations are only well-defined when the subject and object can be located on the same Sequence (though this may be something that is inferred rather than known *a priori*). In interpreting these relations, it is important to remember that for Range objects, the start and end indices refer to whole bases/residues such that a Range with end equal to 9 meets a Range with start equal to 10, while it strictlyPrecedes a Cut with at equal to 10.

objects is allowed because it is plausible that a designer might want to specify that an Interaction will exist, even if its participants have not yet been determined.

| Interaction Type | URL for SBO Term |
|---|---|
| Inhibition | http://identifiers.org/SBO:0000169 |
| Stimulation | http://identifiers.org/SBO:0000170 |
| Biochemical Reaction | http://identifiers.org/SBO:0000176 |
| Non-Covalent Binding | http://identifiers.org/SBO:0000177 |
| Degradation | http://identifiers.org/SBO:0000179 |
| Genetic Production | http://identifiers.org/SBO:0000589 |
| Control | http://identifiers.org/SBO:0000168 |

Table 11: Partial list of SBO terms to specify the `type` property of an `Interaction`.

##### 6.4.4.1 Participation

Each `Participation` (see Figure 13) represents how a particular `Feature` behaves in its parent `Interaction`.

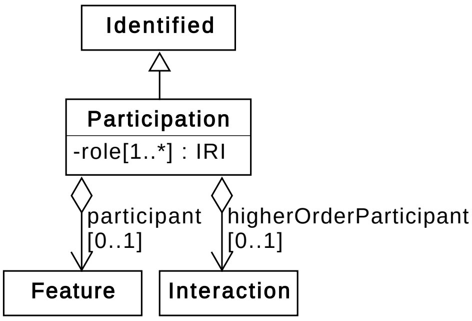

Figure 13: Diagram of the `Participation` class and its associated properties.

***The `role` property***

A `Participation` is REQUIRED to have one or more `role` properties, each of type `IRI`, that describes the behavior of a `Participation` (and by extension its referenced `Feature`) in the context of its parent `Interaction`.

Each `role` property MUST identify terms from appropriate ontologies. It is RECOMMENDED that exactly one `IRI` specified by a `role` property refer to a term from the participant role branch of the SBO. Table 12 provides a partial list of possible SBO terms for the `role` properties and their corresponding `IRI`s.

If a `Participation` is well described by one of the terms from Table 12, then a `role` property MUST refer to the `IRI` that identifies this term. Also, if a `Participation` belongs to an `Interaction` that has a type listed in Table 11, then the `Participation` SHOULD have a role that is cross-listed with this type in Table 12. Lastly, if there are multiple `role` properties for a `Participation`, then they MUST identify non-conflicting terms. For example, the SBO terms “stimulator” and “inhibitor” would conflict.

***The `participant` property***

The `participant` property indicates a `Feature` object that plays the designated role in its parent `Interaction` object. Precisely one value MUST be specified for precisely one of `participant` or `higherOrderParticipant`.

***The `higherOrderParticipant` property***

The `higherOrderParticipant` property indicates an `Interaction` object that plays the designated role in its parent `Interaction` object. Precisely one value MUST be specified for precisely one of `participant` or `higherOrderParticipant`.

| Participation Role | URL for SBO Term | Interaction Types |
|---|---|---|
| Inhibitor | http://identifiers.org/SBO:0000020 | Inhibition |
| Inhibited | http://identifiers.org/SBO:0000642 | Inhibition |
| Stimulator | http://identifiers.org/SBO:0000459 | Stimulation |
| Stimulated | http://identifiers.org/SBO:0000643 | Stimulation |
| Reactant | http://identifiers.org/SBO:0000010 | Non-Covalent Binding, Degradation Biochemical Reaction |
| Product | http://identifiers.org/SBO:0000011 | Non-Covalent Binding, Genetic Production, Biochemical Reaction |
| Promoter | http://identifiers.org/SBO:0000598 | Inhibition, Stimulation, Genetic Production |
| Modifier | http://identifiers.org/SBO:0000019 | Biochemical Reaction, Control |
| Modified | http://identifiers.org/SBO:0000644 | Biochemical Reaction, Control |
| Template | http://identifiers.org/SBO:0000645 | Genetic Production |

Table 12: Partial list of SBO terms to specify the `role` properties of a `Participation`.

#### 6.4.5 *Interface*

The `Interface` class (shown in Figure 14) is a way of explicitly specifying the interface of a `Component`.

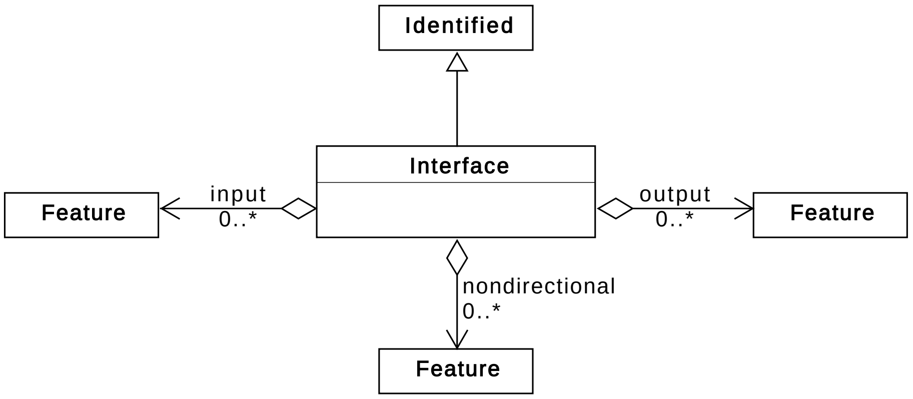

Figure 14: Diagram of the `Interface` class and its associated properties.

##### *The* `input` *property*

An `Interface` MAY have any number of `input` properties, each of type `IRI`, that MUST reference a `Feature` object in the same `Component`.

##### *The* `output` *property*

An `Interface` MAY have any number of `output` properties, each of type `IRI`, that MUST reference a `Feature` object in the same `Component`.

##### *The* `nondirectional` *property*

An `Interface` MAY have any number of `nondirectional` properties, each of type `IRI`, that MUST reference a `Feature` object in the same `Component`. Note that nondirectional can imply both bidirectional as well as situations where there are no flows (for instance – a physical interface).

### 6.5 CombinatorialDerivation

The purpose of the CombinatorialDerivation class is to specify combinatorial biological designs without having to specify every possible design variant. For example, a CombinatorialDerivation can be used to specify a library of reporter gene variants that include different promoters and RBSs without having to specify a Component for every possible combination of promoter, RBS, and CDS in the library. Component objects that realize a CombinatorialDerivation can be derived in accordance with the class properties template, hasVariableFeature, and strategy (see Figure 15).

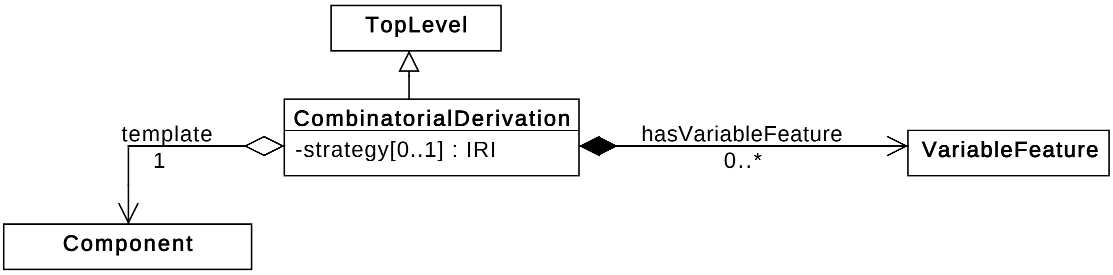

Figure 15: Diagram of the CombinatorialDerivation class and its associated properties.

##### *The template property*

The template property is REQUIRED and MUST contain a IRI that refers to a Component. This Component is expected to serve as a template for the derivation of new Component objects. Consequently, its hasFeature properties SHOULD contain one or more Feature objects that will serve as the variables whose values are set during derivation (referred to hereafter as template Feature objects). Its other property values describe aspects of the template that will not change based on the values that may be varied.

##### *The hasVariableFeature property*

Each VariableFeature child of a CombinatorialDerivation defines the set of possible values for one of the variables in the template. A CombinatorialDerivation object can have zero or more hasVariableFeature properties, each of type IRI, specifying a VariableFeature. The set of hasVariableFeature properties MUST NOT contain two or more VariableFeature objects that refer to the same template sbolFeature via their variable properties (i.e., do not define the same variable twice).

The variable properties of VariableFeature objects determined which Feature objects in the template are modified in a derived Component, and which ones will not be changed. In particular, we will refer to a Feature in the template Component that is referred to by some variable property as a variable Feature, and one that is not referred to by any as a static Feature.

##### *The strategy property*

The strategy property is OPTIONAL and has a data type of IRI. Table 13 provides a list of REQUIRED strategy URLs. If the strategy property is not empty, then it MUST contain a URL from Table 13. This property recommends how many Component objects SHOULD be derived from the template Component.

##### *Executing a derivation*

When a CombinatorialDerivation is evaluated to produce a set of derived Component objects, the relationship between the two SHOULD be recorded by means of prov:wasDerivedFrom properties. In particular:

| Strategy URL | Description |
|---|---|
| http://sbols.org/v3#enumerate | Derivation SHOULD produce all possible Component objects specified by the CombinatorialDerivation. |
| http://sbols.org/v3#sample | Derivation SHOULD produce a subset of possible Component objects specified by CombinatorialDerivation. The manner in which this subset is chosen is left unspecified. |

Table 13: REQUIRED URLs for the strategy property.

- Any derived Component SHOULD have a prov:wasDerivedFrom property that refers to the CombinatorialDerivation.
- Any Feature in a derived Component SHOULD have a prov:wasDerivedFrom property that refers to its corresponding Feature in the template Component.
- Any Collection produced by the derivation process and containing only derived Component objects SHOULD also have a prov:wasDerivedFrom property that refers to the CombinatorialDerivation.

All derived objects MUST be consistent with the specification provided in the CombinatorialDerivation. In particular:

- Every value of the type and role properties of the template Component SHOULD be contained in the values of the corresponding properties in each derived Component.
- Any static Feature in the template Component SHOULD correspond to a Feature with identical properties in each derived Component.
- Any variable Feature in the template Component SHOULD be replaced in each derived Component by a number of Feature objects constrained by the number specified by the cardinality property of the VariableFeature (see Table 14).
- Each property of a Feature object in the derived Component that replaces a variable Feature in the template Component MUST be derived from the values of the associated VariableFeature.
- All derived Feature object MUST follow the restriction properties of any Constraint objects that refer to their corresponding template Feature. This will typically be used to rule out illegal combinations of variable values.
- The role property of a derived Feature SHOULD contain the same values as the role property did in the template Feature.
- The type property of a derived Feature or its type-determining referent (instanceOf for SubComponent, or that determined for the Feature referred to by a ComponentReference) SHOULD contain the same values as the type property did in the template Feature or its type-determining referent.

#### 6.5.1 VariableFeature

As described above, the VariableFeature class specifies a variable and set of values that will replace one of the Feature objects in the template of a CombinatorialDerivation. The variable is specified by the variable property, and the set of values is defined by the union of Component objects referred to by the variant, variantCollection, and variantDerivation properties.

Note that this union is intended to be a set and not a multi-set. For example, if the variant property contains a Component $A$ and the variantCollection property has a Collection containing both Component $A$ and Component $B$, then $A$ SHOULD NOT be selected twice during enumeration, and it SHOULD NOT be selected twice as much as $B$ during sampling.

Given a set of values linked from a VariableFeature, it SHOULD be the case that all value are of type om:Measure or else all values are of type Feature. At present, it is explicitly left undefined how derivation of new components ought to handle mixtures of om:Measure and Feature values.

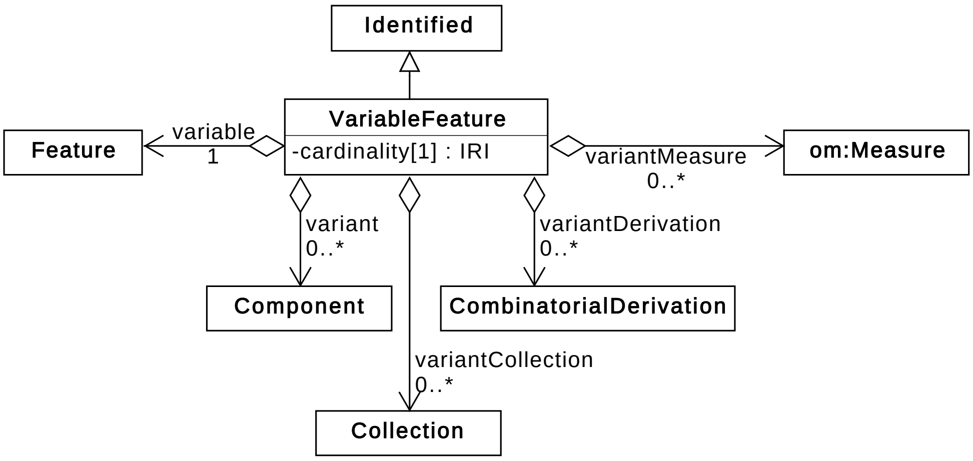

Figure 16: Diagram of the VariableFeature class and its associated properties.

##### *The `variable` property*

The variable property is REQUIRED and MUST contain a IRI that refers to a template Feature in the template Component referred to by this VariableFeature's parent CombinatorialDerivation

##### *The `variantMeasure` property*

A VariableFeature object can have zero or more variantMeasure properties, each of type IRI, specifying a om:Measure object. This property specifies numerical values that are options to be applied to the variable Feature from the template when deriving a new Component.

Note that because a om:Measure is not a TopLevel, the vlaues of variantMeasure must be child objects of the VariableFeature.

##### *The `variant` property*

A VariableFeature object can have zero or more variant properties, each of type IRI, specifying a Component object. This property specifies individual Component objects to serve as options when deriving a new Feature for the variable Feature from the template.

##### *The `variantCollection` property*

A VariableFeature object can have zero or more variantCollection properties, each of type IRI, specifying a Collection object. Such a Collection MUST NOT contain any objects besides Component objects or Collection objects that themselves contain only Component or Collection objects. This property enables the specification of existing groups of Component objects to serve as options.

##### *The `variantDerivation` property*

A VariableFeature object can have zero or more variantDerivation properties, each of type IRI, specifying a CombinatorialDerivation object. This property enables the specification of Component objects derived in accordance with another CombinatorialDerivation to serve as options when deriving a new Feature for the variable

Feature from the template. The variantDerivation properties of a VariableFeature MUST NOT refer to the CombinatorialDerivation that contains this VariableFeature. Furthermore, such VariableFeature objects MUST NOT form a cyclical chain of references via their variantDerivation properties and the CombinatorialDerivation objects that contain them.

***The cardinality property***

The cardinality property is REQUIRED and has type of IRI. This property specifies how many Feature objects SHOULD be derived from the template Feature during the derivation of a new Component. The value of this property MUST come from the URLs provided in Table 14.

| Cardinality URL | Description |
|---|---|
| http://sbols.org/v3#zeroOrOne | No more than one Feature in the derived Component SHOULD have a prov:wasDerivedFrom property that refers to the template Feature. |
| http://sbols.org/v3#one | Exactly one Feature in the derived Component SHOULD have a prov:wasDerivedFrom property that refers to the template Feature. |
| http://sbols.org/v3#zeroOrMore | Any number of Feature objects in the derived Component MAY have prov:wasDerivedFrom properties that refer to the template Feature. |
| http://sbols.org/v3#oneOrMore | At least one Feature in the derived Component SHOULD have a prov:wasDerivedFrom property that refers to the template Feature. |

Table 14: REQUIRED URLs for the cardinality property.

### 6.6 Implementation

An Implementation represents a realized instance of a Component, such a sample of DNA resulting from fabricating a genetic design or an aliquot of a specified reagent. Importantly, an Implementation can be associated with a laboratory sample that was already built, or that is planned to be built in the future. An Implementation can also represent virtual and simulated instances. An Implementation may be linked back to its original design using the prov:wasDerivedFrom property inherited from the Identified superclass. An Implementation may also link to a Component that specifies its realized structure and/or function.

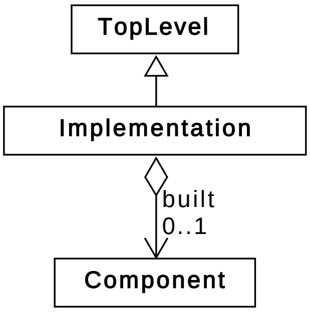

Figure 17: Diagram of the Implementation class and its associated properties.

##### *The* **built** *property*

The built property is OPTIONAL and MAY contain a IRI that MUST refer to a Component. This Component is intended to describe the actual physical structure and/or functional behavior of the Implementation. When the built property refers to a Component that is also linked to the Implementation via PROV-O properties such as prov:wasDerivedFrom (see Section A.1), it can be inferred that the actual structure and/or function of the Implementation matches its original design. When the built property refers to a different Component, it can be inferred that the Implementation has deviated from the original design. For example, the latter could be used to document when the DNA sequencing results for an assembled construct do not match the original target sequence.

### 6.7 ExperimentalData

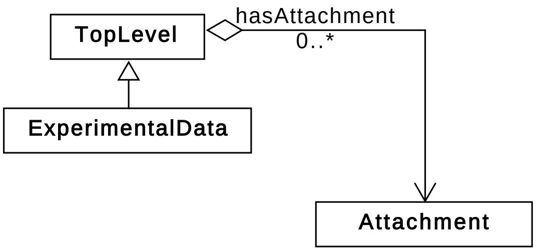

Figure 18: Diagram of the ExperimentalData class and its associated properties.

The purpose of the ExperimentalData class is to aggregate links to experimental data files. An ExperimentalData is typically associated with a single sample, lab instrument, or experimental condition and can be used to describe the output of the test phase of a design-build-test-learn workflow. For an example of the latter, see Figure 28.

As shown in Figure 18, the ExperimentalData class aggregates links to experimental data files using the OPTIONAL hasAttachment property that it inherits from the TopLevel class.

### 6.8 Model

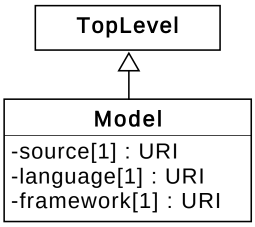

Figure 19: Diagram of the Model class and its associated properties.

The purpose of the Model class is to serve as a placeholder for an external computational model and provide additional meta-data to enable better reasoning about the contents of this model. In this way, there is minimal duplication of standardization efforts and users of SBOL can elaborate descriptions of Component function in the language of their choice.

The meta-data provided by the `Model` class include the following properties: the `source` or location of the actual content of the model, the `language` in which the model is implemented, and the model's `framework`.

##### *The `source` property*

The `source` property is REQUIRED and MUST contain a `IRI` reference to the source file for a model.

##### *The `language` property*

The `language` property is REQUIRED and MUST contain a `IRI` that specifies the language in which the model is implemented. It is RECOMMENDED that this `IRI` refer to a term from the EMBRACE Data and Methods (EDAM) ontology. Table 15 provides a list of a few suggested languages from this ontology and their `IRI`s. If the `language` property of a `Model` is well-described by one these terms, then it MUST contain the `IRI` for this term as its value.

| Model Language | URL for EDAM Term |
|---|---|
| SBML | `http://identifiers.org/EDAM:format_2585` |
| CellML | `http://identifiers.org/EDAM:format_3240` |
| BioPAX | `http://identifiers.org/EDAM:format_3156` |

Table 15: Terms from the EDAM ontology to specify the `language` property of a `Model`.

##### *The `framework` property*

The `framework` property is REQUIRED and MUST contain a `IRI` that specifies the framework in which the model is implemented. It is RECOMMENDED this `IRI` refer to a term from the modeling framework branch of the SBO when possible. A few suggested modeling frameworks and their corresponding `IRI`s are shown in Table 16. If the `framework` property of a `Model` is well-described by one these terms, then it MUST contain the `IRI` for this term as its value.

| Framework | URL for SBO Term |
|---|---|
| Continuous | `http://identifiers.org/SBO:0000062` |
| Discrete | `http://identifiers.org/SBO:0000063` |

Table 16: SBO terms to specify the `framework` property of a `Model`.

### 6.9 Collection

The `Collection` class is a class that groups together a set of `TopLevel` objects that have something in common. Some examples of `Collection` objects:

- Results of a query to find all `Component` objects in a repository that function as promoters.
- A set of `Component` objects representing a library of genetic logic gates.
- A "parts list" for `Component` with a complex design, containing both that component and all of the `Component`, `Sequence`, and `Model` objects used to provide its full specification.

##### *The `member` property*

A `Collection` object can have zero or more `member` properties, each of type `IRI` specifying a `TopLevel` object.

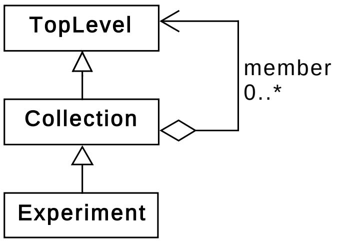

Figure 20: Diagram of the Collection class and its associated properties.

#### 6.9.1 Experiment

The purpose of the Experiment class is to aggregate ExperimentalData objects for subsequent analysis, usually in accordance with an experimental design. Namely, the member properties of an Experiment MUST refer to ExperimentalData objects.

### 6.10 Attachment

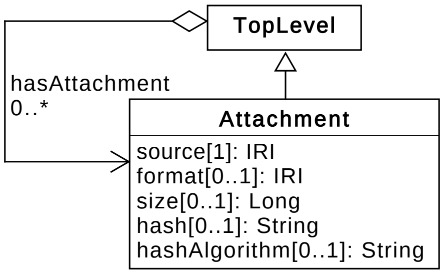

Figure 21: Diagram of the Attachment class and its associated properties.

The purpose of the Attachment class is to serve as a general container for data files, especially experimental data files. It provides a means for linking files and metadata to SBOL designs.

The meta-data provided by the Attachment class include the following properties: the source or location of the actual file of the attachment, the format of the file, the size of the file, and the hash for the file.

##### *The source property*

The source property is REQUIRED and MUST contain a IRI reference to the source file.

##### *The format property*

The format property is OPTIONAL and MAY contain a IRI that specifies the format of the attached file. It is RECOMMENDED that this IRI refer to a term from the EMBRACE Data and Methods (EDAM) ontology.

##### *The size property*

The size property is OPTIONAL and MAY contain a long indicating the file size in bytes.

##### *The `hash` property*

The `hash` property is OPTIONAL and MAY contain a hash value for the file contents represented as a hexadecimal digest.

##### *The `hashAlgorithm` property*

The `hashAlgorithm` property is OPTIONAL and MAY contain the name of the hash algorithm used to generate the value of the `hash` property. The value of this property SHOULD be a hash name string from the IANA Named Information Hash Algorithm Registry, of which `sha3-256` is currently RECOMMENDED. If the `hash` property is set, then `hashAlgorithm` MUST be set as well.

### 6.11 Annotation and Extension of SBOL

SBOL intentionally does not attempt to describe how all types of biological design data should be captured, since many of these data types (e.g., biological context and design performance metrics) are already covered by other standards, or lack a clear consensus on their proper representation, or are outside of the scope of SBOL.

SBOL is built upon the Resource Description Framework (RDF), and therefore can be used in conjunction with complementary standards as described in Section A. For example, use of the PROV-O ontology is recommended to capture provenance (see Section A.1). Additionally, user-defined RDF can be used in conjunction with SBOL objects to capture custom application-specific information that does not yet have a standardized representation. This annotation and extension mechanism is designed to enable new types of data to be easily incorporated into the SBOL standard once there is community consensus on their proper representation.

Several methods are supported for connecting the SBOL data model with other types of application-specific data:

- Custom data can be added to an SBOL object by annotating that object with non-conflicting properties. These properties could contain `literal` data types such as `String`s or `IRI`s that require a resolution mechanism to obtain external data. An example is annotating a `Component` with a property that contains a `String` description and `IRI` for the parts registry from which its source data was originally imported.
- SBOL object classes can be extended to custom classes that add additional information. This works just like adding custom data via non-conflicting properties, except that the object receives both an `rdf:type` for the SBOL class that has been extended and also an `rdf:type` specifying the extension class.
- Custom data in the form of independent objects can participate in the SBOL data model if they are assigned one of the SBOL types `Identified` or `TopLevel`. An example is an RDF object that is annotated such that it represents a data sheet that describes the performance of a `Component` in a particular context.
- Finally, just as custom objects can be embedded in an SBOL document, external documents can embed or refer to SBOL objects. Support for this last case is not explicitly provided in this specification. Rather, this case depends on the external non-SBOL system managing its relationship to SBOL and data serialized in RDF, and is included here for completeness.

Each `Identified` object MAY be annotated with application-specific properties, which MUST be labelled using RDF predicates outside of the SBOL namespace. Additionally, application-specific types may be used in conjunction with the SBOL data model. These application-specific types MUST have at least two `rdf:type` properties: one type outside of the SBOL namespace AND an additional SBOL type of either:

- `TopLevel`, if the object is to be considered an SBOL top level (i.e., not owned by another object)
- `Identified`, if the object is not to be considered an SBOL top level (i.e., is owned by another object)
- The most specific applicable SBOL type, if the object is an instance of a custom class extending an SBOL class.

As with SBOL `Identified` objects, custom `Identified` objects (and thus also all other custom objects) MAY also include the properties `displayId`, `name`, `description`, etc.

## 7 Recommended Best Practices

### 7.1 SBOL Versions

To differentiate between major versions of SBOL, different namespaces are used. For example, SBOL3 has the namespace `http://sbols.org/v3#`, while SBOL2 has the namespace `http://sbols.org/v2#`. These different versions of SBOL SHOULD NOT be semantically mixed. For example, an SBOL 3.x `SubComponent` SHOULD NOT refer to an SBOL 2.x `ComponentInstance`, and, likewise, an SBOL 2.x `ComponentInstance` SHOULD NOT refer to an SBOL 1.x `DnaComponent`.

### 7.2 Compliant SBOL Objects

Maintaining unique IRIs for all SBOL objects can be challenging. To reduce this burden, users of SBOL 3.x are encouraged to follow a few simple rules when constructing URLs and related properties for SBOL objects. When these rules are followed in constructing an SBOL object, we say that this object is *compliant*. These rules are as follows:

Compliant URLs for `TopLevel` objects MUST conform to the following pattern:

⟨namespace⟩/⟨collection_structure⟩/⟨displayId⟩

The ⟨namespace⟩ token MAY further decompose into ⟨domain⟩/⟨root⟩ tokens. The ⟨root⟩ and ⟨collection_structure⟩ tokens may optionally be omitted; alternatively, they may consist of an arbitrary number of delimiter-separated layers. Note that this pattern means that SBOL-compliant `URL`s can be automatically decomposed with the aid of a `TopLevel` object's `hasNamespace` property. SBOL-compliant objects can be easily remapped into new namespaces by changing only the ⟨namespace⟩.

Consider, for example, the SBOL-compliant `URL`:

"https://synbiohub.org/igem/2017_distribution/promoters/constitutive/BBa_J23101"

for a `Component` with a `hasNamespace` value "https://synbiohub.org/igem/2017_distribution". This `URL` can be decomposed as follows:

| | |
|---|---|
| namespace: | "https://synbiohub.org/igem/2017_distribution" |
| domain: | "https://synbiohub.org" |
| root: | "igem/2017_distribution" |
| collection: | "promoters/constitutive" |
| displayId: | "BBa_J23101" |

SBOL-compliant URLs also facilitate auto-construction of child objects with unique `URL`s. Child objects of `TopLevel` objects with compliant `URL`s MUST conform to the following pattern: "⟨parent_url⟩/⟨child_type⟩⟨child_type_counter⟩" where the ⟨parent_url⟩ refers to the URL of the parent object, the ⟨child_type⟩ refers to the SBOL class of the child object, and ⟨child_type_counter⟩ is a unique index for the child object. The ⟨child_type_counter⟩ of a new object SHOULD be calculated at time of object creation as 1 + the maximum ⟨child_type_counter⟩ for each ⟨child_type⟩ object in the parent (e.g., "⟨parent_url⟩/SequenceAnnotation37"). Note that numbering is independent for each type, so a `Component` can have children "SubComponent37" and "Constraint37".

All examples in this specification use compliant `URL`s.

### 7.3 Versioning SBOL Objects

SBOL 3.x does not specify an explicit versioning scheme. Rather it is left for experimentation across different tools. This allows version information to be included in the root (e.g., GitHub style: “igem/HEAD/”), collection structure (e.g., “promoters/constitutive/2/”), in tool-specific conventions on `displayId` (e.g., “BBa_J23101_v2”) or in information outside of the `IRI` (e.g., by attaching `prov:wasRevisionOf` properties).

### 7.4 Annotations: Embedded Objects vs. External References

When annotating an SBOL document with additional information, there are two general methods that can be used:

- Embed the information in the SBOL document using properties outside of the SBOL namespace.
- Store the information separately and annotate the SBOL document with `IRI`s that point to it.

In theory, either method can be used in any case. (Note that a third case not discussed here is to annotate external objects with links to SBOL documents, rather than annotating SBOL documents with links to external objects.)

In practice, embedding large amounts of non-SBOL data into SBOL documents is likely to cause problems for people and software tools trying to manage and exchange such documents. Therefore, it is RECOMMENDED that small amounts of information (e.g., design notes or preferred graphical layout) be embedded in the SBOL model, while large amounts of information (e.g., the contents of the scientific publication from which a model was derived or flow cytometry data that characterizes performance) be linked with IRIs pointing to external resources. The boundary between “small” and “large” is left deliberately vague, recognizing that it will likely depend on the particulars of a given SBOL application.

### 7.5 Completeness and Validation

RDF documents containing serialized SBOL objects might or might not be entirely self-contained. A SBOL document is self-contained or “complete” if every SBOL object referred to in the document is contained in the document. It is RECOMMENDED that serializations be complete whenever practical. In order words, when serializing an SBOL object, serialize all of the other objects that it points to, then serialize all of the other objects that these objects point to, etc., until the document is complete.

It is important to note that there is no guarantee that an RDF document contains valid SBOL. When SBOL objects are read from an RDF document, the program doing so SHOULD verify that all of the property values encoded therein have the correct data type (e.g., that the object pointed to by the `Sequence` property of a `Component` is really a `Sequence`). For complete files, this validation can be carried out entirely locally. For files that are not complete, an implementation either needs to have a means of validating those external references (e.g., by retrieving them from a repository), or it needs to mark them as unverified and not depend on their correctness.

### 7.6 Recommended Ontologies for External Terms

External ontologies and controlled vocabularies are an integral part of SBOL. SBOL uses `IRI`s (typically `URL`s to access existing biological information through these resources. New SBOL-specific terms are defined only when necessary. For example, `Component` `type`s, such as DNA or protein, are described using Systems Biology Ontology (SBO) terms. Similarly, the `role`s of a DNA or RNA `Component` are described via Sequence Ontology (SO) terms. Although RECOMMENDED ontologies have been indicated in relevant sections where possible, other resources providing similar terms can also be used. A summary of these external sources can be found in Table 17.

The IRIs for ontological terms SHOULD be URLs from identifiers.org. However, it is acceptable to use terms from purl.org as an alternative, for example when RDF tooling requires URLs to be represented as compliant QNames. SBOL software may convert between these forms as required.

| SBOL Entity | Property | Preferred External Resource | More Information |
|---|---|---|---|
| **Component** | type | SBO (physical entity branch) | http://www.ebi.ac.uk/sbo/main/ |
| | type | SO (nucleic acid topology) | http://www.sequenceontology.org |
| | role | SO (*DNA* or *RNA*) | http://www.sequenceontology.org |
| | role | CHEBI (*small molecule*) | https://www.ebi.ac.uk/chebi/ |
| | role | PubChem (*small molecule*) | https://pubchem.ncbi.nlm.nih.gov/ |
| | role | UniProt (*protein*) | https://www.uniprot.org/ |
| | role | NCIT (*samples*) | https://ncithesaurus.nci.nih.gov/ |
| **Interaction** | type | SBO (occurring entity branch) | http://www.ebi.ac.uk/sbo/main/ |
| **Participation** | role | SBO (participant roles branch) | http://www.ebi.ac.uk/sbo/main/ |
| **Model** | language | EDAM | http://bioportal.bioontology.org/ontologies/EDAM |
| | framework | SBO (modeling framework branch) | http://www.ebi.ac.uk/sbo/main/ |
| **om:Measure** | type | SBO (systems description parameters) | http://www.ebi.ac.uk/sbo/main/ |

Table 17: Preferred external resources from which to draw values for various SBOL properties.

### 7.7 Annotating Entities with Date & Time

Entities in an SBOL document can be annotated with creation and modification dates. It is RECOMMENDED that predicates, or properties, from DCMI Metadata Terms SHOULD be used to include date and time information. The `created` and `modified` terms SHOULD respectively be used to annotate SBOL entities with creation and modification dates. Date and time values SHOULD be expressed using the XML Schema `DateTime` datatype (Biron et al., 2004). For example, "`2016-03-16T20:12:00Z`" specifies that the day is 16 March 2016 and the time is 20:12pm in UTC (Coordinated Universal Time).

### 7.8 Annotating Entities with Authorship information

Authorship information should ideally be added to `TopLevel` entities where possible. It is RECOMMENDED that the `creator` DCMI Metadata term SHOULD be used to annotate SBOL entities with authorship information using free text. This property can be repeated for each author.

### 7.9 Host Context / Ontologies for Experiments

#### 7.9.1 Mixtures via Components

Any `Component` can be interpreted as specifying a mixture of the material entity (SBO:0000240) `Feature`s that it includes. The amount of each such instance included in the mixture SHOULD be specified by attaching a `om:Measure` with a `type` set to the appropriate SBO term. The SBO terms that are RECOMMENDED as appropriate are members of the Systems Description Parameter (SBO:0000545) branch of SBO. Examples include:

- SBO:0000540: fraction of an entity pool (e.g., 1/3 CHO cells, 2/3 HEK cells)
- SBO:0000472: molar concentration of an entity (e.g., 1 mM arabinose)
- SBO:0000361: amount of an entity pool (e.g., 200 uL M9 media)

Mixtures MAY be defined recursively, as mixtures of mixtures of mixtures, etc.

#### 7.9.2 Media, Inducers, and Other Reagents

Each reagent, whether "atomic" (e.g., rainbow bead control) or mixture (e.g., M9 media), SHOULD be represented as a `Component` and/or as a `Feature` of a `Component` in which the reagent is used. For example, a custom media mixture might be defined as a Component and used as a SubComponent, while a commercially supplied reagent might be used as an ExternallyDefined feature linking to its PubChem identifiers.

The roles of reagents may vary in context: for example, arabinose may serve as an inducer or as a media carbon source. As such, contextual role SHOULD be indicated by an NCI Thesaurus (NCIT) term in a role property of the Feature. Examples include:

- NCIT:C64356: Positive Control
- NCIT:C12508: Cell
- NCIT:C85504: Growth Medium
- NCIT:C14419: Organism Strain
- NCIT:C120268: Inducer

For more information on representing cells, strains, plasmids, and genomes, see Section 7.10.1

#### 7.9.3 Samples

A complete specification of a sample SHOULD be a Component that includes at least:

- A Feature instantiating each strain in the sample
- A Feature for the media or buffer
- A Feature for each additional reagent added to the media (e.g., inducers, antibiotics)
- om:Measures on each of these specifying the amount in the sample
- om:Measures on the Component for each environmental parameter (e.g., temperature, pH, culturing time)

#### 7.9.4 Other Experimental Parameters

In order to deal with parameters associated with the context in general but not specific instances, e.g., temperature, pH, total sample volume, the hasMeasure property of Identified can be used. The hasMeasure of a Component provides context-free information (e.g., the pH of M9 media, the GC-content of a GFP coding sequence), while the hasMeasure of a material entity (SBO:0000240) Feature provides a measurement in context (e.g., the dosage of arabinose in a sample).

Values of these parameters SHOULD be specified by attaching a om:Measure with a type set to the appropriate SBO term. The SBO terms that are RECOMMENDED as appropriate are members of the Systems Description Parameter (SBO:0000545) branch of SBO. Examples include:

- SBO:0000147: thermodynamic temperature (e.g., culturing at 27 C)
- SBO:0000332: half-life of an exponential decay (e.g., decay rate of a gRNA)
- SBO:0000304: pH (e.g., pH of M9 media)

### 7.10 Multicellular System Designs

SBOL has been used extensively to represent designs in homogeneous systems, where the same design is implemented in every cell. However, in recent years there has been increasing interest in multicellular systems, where biological designs are split across multiple cells to optimize the system behavior and function. Therefore, there is a need to define a set of best practices so that multicellular systems can be captured using SBOL in a standard way.

#### 7.10.1 Representing Cell Types

To represent multicellular systems using SBOL, it is first necessary to represent cells. When doing so, it is important to be able to capture the following information: (i) taxonomy of the strain used, (ii) interactions occurring within cells of this type, and (iii) components inside the type of cell (e.g. genomes, plasmids). The approach RECOMMENDED in this section is capable of capturing this information, as shown in the example in Figure 22. It uses a `Component` to represent a system that contains cells of the given type. The cells themselves are represented by a `Feature` inside the `Component`, in this case a `SubComponent` that is an `instanceOf` a `Component` capturing information about the species and strain of the cell in the design. This `Component` has a `type` of "cell" from the Cell Ontology (CL:0000000), and a `role` of "physical compartment" (SBO:0000290). Taxonomic information is captured by annotating the class instance with a IRI for an entry in the NCBI Taxonomy Database.

As usual, other entities besides the cell that are relevant to the design are also captured as `Feature`s. When these are contained within the cell, they are captured using a `Constraint` with restriction `contains` with the cell as `subject` and contained object as `object`. Interactions which occur in this system are captured using the `Interaction` and `Participation` classes. Interactions which occur within the cell are specified by `Interaction` classes which contain the `Feature` instance representing the cell as a `participant` with a `role` of "physical compartment" (SBO:0000290).

#### 7.10.2 Multiple Cell Types in a Single Design

The same approach can be extended to represent systems with multiple types of cells. The multicellular system can be represented as a `Component` that includes each strain of cell as a `Feature`, in this example a `SubComponent` that is an `instanceOf` a `Component` defining its strain. Interactions and constraints, such as a molecule that both strains interact with, are implemented using `ComponentReference`s to link to the definitions within each cell system description. An example is shown in Figure 23.

#### 7.10.3 Cell Ratios

The proportion of cell types present in a multicellular system can be captured using `om:Measure` on the representations of cells in the design. As a best practice, the value of these measure classes is a percentage less than or equal to 100%, representing the amount of a cell type present in the system compared to all other cell types present. Therefore, the sum of all these values specified in the system will typically be equal to 100%, though this may not be the case if the system is not completely defined. An example is shown in Figure 24.

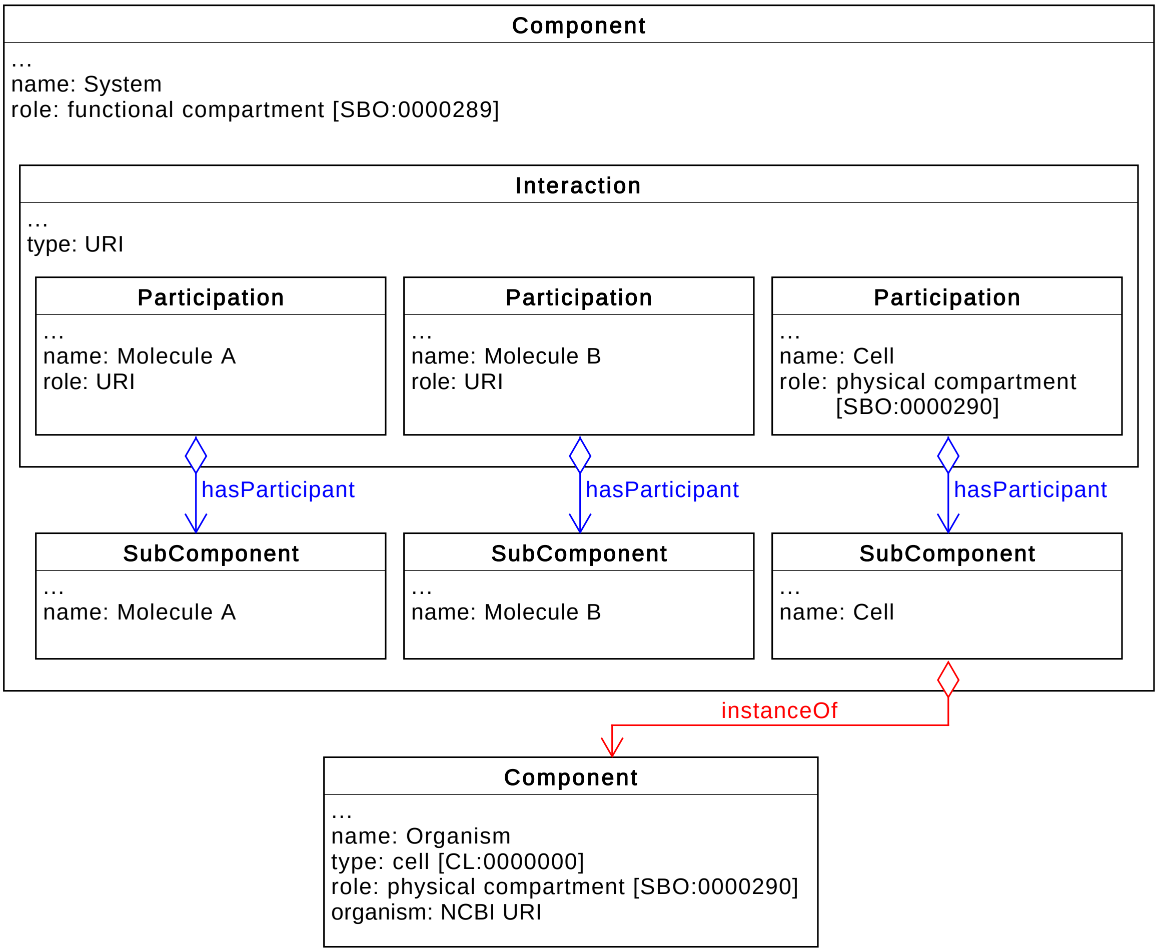

Figure 22: This is a proposed approach for capturing cell designs in SBOL. A `Component` annotated with a IRI pointing to an entry in the NCBI Taxonomy Database is used to capture information about the cell's strain/species. The `Component` has a type of "Cell" from the Gene Ontology (GO), and a role of "physical compartment". Another `Component` is used to represent a system in which the cell is implemented. Entities, including the cell, are instantiated as `Features`, and processes are captured using the `Interaction` class. Processes that are contained within the cell are represented by including the cell as a participant with a role of "physical compartment".

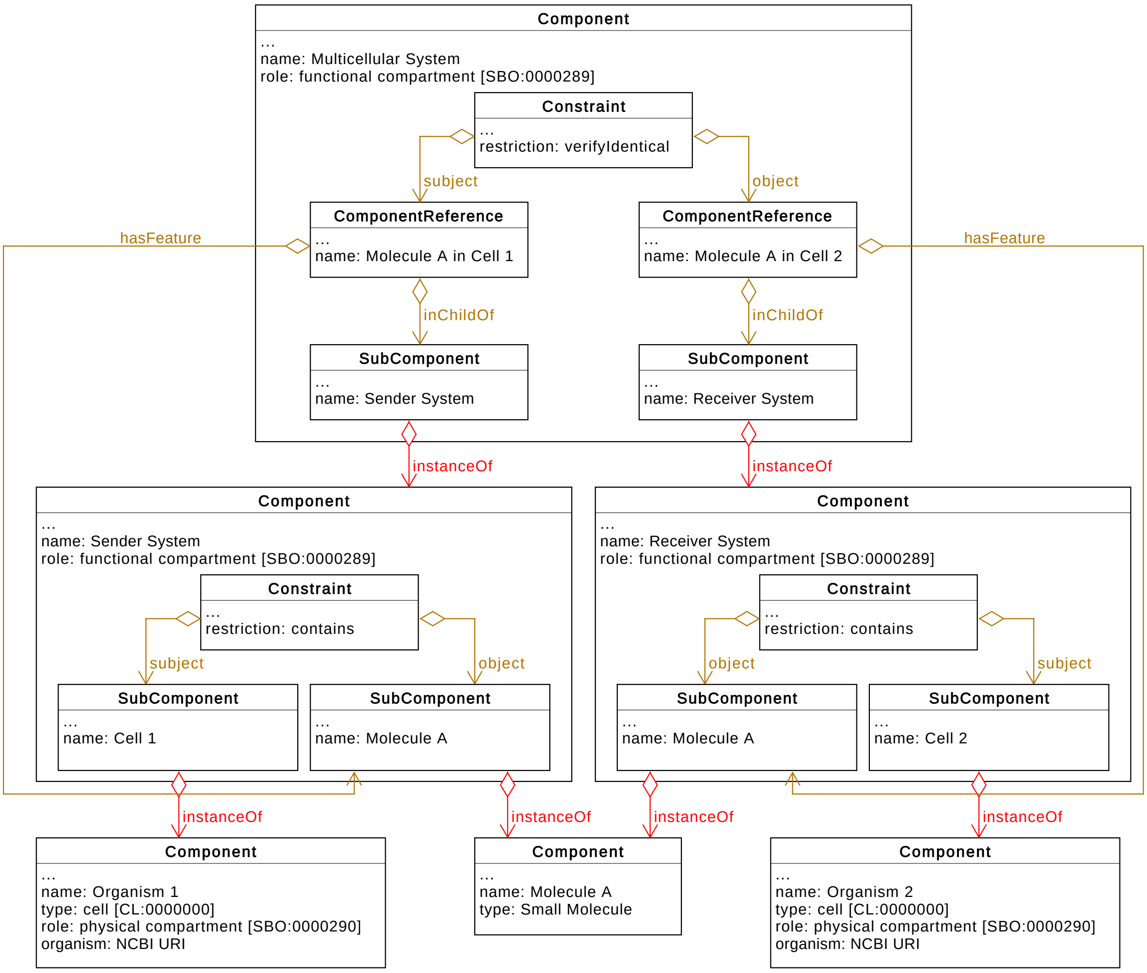

Figure 23: Captured here is a design involving two cells which both interact with the small molecule "Molecule A". Designs for the sender and receiver systems are captured using constraint to show that each of these cells interacts with the Molecule A contained within it. The overall multicellular system is represented by a `Component` with a `role` of "functional compartment", which is an SBO term. The two systems are included in this multicellular design as `Feature`s, and the fact that Molecule A is shared between systems is indicated with a constraint.

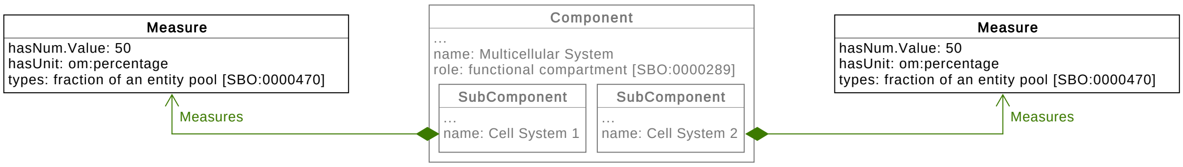

Figure 24: Annotating class instances with cellular proportions. Instances of the Measure class are used to capture the percentage of each cell type present in the multicellular system design.

## 8 SBOL RDF Serialization

In order for SBOL objects to be readily stored and exchanged, it is important that they are able to be *serialized*, i.e., converted to a sequence of bytes that can be stored in a file or exchanged over a network. The serialization format for SBOL is designed to meet several competing requirements. First, SBOL needs to support ad-hoc annotations and extensions. Second, SBOL needs to support processing by general database and semantic web software tools that have little or no knowledge of the SBOL data model. Finally, it ought to be relatively simple to write a new software implementation, so that SBOL can be readily used even in software environments where community-maintained implementations are not available.

To meet these goals, SBOL builds upon the Resource Description Framework (RDF). RDF is an abstract language for describing conceptual graph-oriented data models, and therefore does not mandate any specific serialization format. Instead, a number of different serialization formats are provided as separate specifications, such as RDF/XML, N-Triples, JSON-LD, and Turtle. These serialization formats are widely supported by RDF libraries such as rdflib for Python and Apache Jena for Java. For example, a simple SBOL definition of pLac can be serialized in RDF/XML as follows:

```
<?xml version="1.0" encoding="utf-8"?>
<rdf:RDF xmlns:rdf="http://www.w3.org/1999/02/22-rdf-syntax-ns#" xmlns:sbol="http://sbols.org/v3#">
  <sbol:Component rdf:about="http://example.com#pLac">
    <sbol:name>pLac</sbol:name>
    <sbol:description>lactose inducible promoter</sbol:description>
    <sbol:sequence rdf:resource="http://example.com#sequence"/>
  </sbol:Component>
  <sbol:Sequence rdf:about="http://example.com#sequence">
    <sbol:encoding rdf:resource="http://sbols.org/v3#iupacNucleicAcid"/>
    <sbol:elements>caatacgcaaaccgcctctccccgcgc</sbol:elements>
  </sbol:Sequence>
</rdf:RDF>
```

Alternatively, the same example can be serialized in Turtle as follows:

```
@prefix sbol: <http://sbols.org/v3#> .
@base <http://example.com#> .
@prefix : <http://example.com#> .

:pLac a sbol:Component ;
    sbol:name "pLac" ;
    sbol:description "lactose inducible promoter" ;
    sbol:sequence :sequence .

:sequence a sbol:Sequence ;
    sbol:encoding <http://sbols.org/v3#iupacNucleicAcid> ;
    sbol:elements "caatacgcaaaccgcctctccccgcgc" .
```

All SBOL libraries SHOULD support at least RDF/XML, N-Triples, JSON-LD, and Turtle. Other SBOL tools SHOULD support at least one of these four formats.

## 9 SBOL Compliance

There are different types of software compliance with respect to the SBOL specification. First, a software tool can either support all classes of the SBOL 3 data model or only its structural subset. The structural subset includes the following classes:

- Sequence
- Component
    - SubComponent
    - ComponentReference
    - LocalSubComponent
    - SequenceFeature
    - Location
    - Constraint
- Collection

Second, an SBOL-compliant software tool can support import of SBOL, export of SBOL, or both. If it supports both import and export, it can do so in either a lossy or lossless fashion.

In order to test import compliance, developers are encouraged to use the SBOL test files found here:
https://github.com/SynBioDex/SBOLTestSuite
Examples of every meaningful subset of objects are provided, including both structural-only SBOL (that is, annotated DNA sequence data) and complete tests.

In order to test export compliance, developers are encouraged to validate SBOL files generated by their software with the SBOL Validator found here:
https://validator.sbolstandard.org
This validator can also be used to check lossless import/export support, since it can compare the data content of files imported and exported by a software tool.

Finally, developers of SBOL-compliant tools are encouraged to notify the SBOL editors (sbol-editors@googlegroups.com) when they have determined that their tool is SBOL compliant, so their tool can be publicly categorized as such on the SBOL website.

## 10 Mapping Between SBOL 1, SBOL 2, and SBOL3

In broad strokes, the SBOL 1 standard focused on conveying physical, structural information, whereas SBOL 2 expanded the scope to include functional aspects as well. The physical information about a designed genetic construct includes the order of its constituents and their descriptions. Specifying the exact locations of these constituents and their sequences allows genetic constructs to be defined unambiguously and reused in other designs. SBOL 2 extended SBOL 1 in several ways: it extends physical descriptions to include entities beyond DNA sequences, and it added support for functional descriptions of designs. SBOL 3 refines the SBOL 2 data model to simplify the representation of common use cases.

### 10.1 Mapping between SBOL 1 and SBOL 2

Figure 25 depicts the mapping of SBOL 1.1 classes to SBOL 2.x classes, indicating corresponding classes/properties by color. The SBOL 2.x `Model` and `ModuleDefinition` classes have no SBOL 1.1 equivalent, and thus are not shown. The mapping from SBOL 1.1 to SBOL 2.x proceeds as follows:

- SBOL 1.1 `Collection` objects containing `DnaComponent` objects map to SBOL 2.x `Collection` objects that contain `ComponentDefinition` objects with DNA `type` properties.
- SBOL 1.1 `DnaComponent` objects map to SBOL 2.x `ComponentDefinition` objects with DNA `type` properties.
- SBOL 1.1 `DnaSequence` objects map to an SBOL 2.x `Sequence` objects with `IUPAC DNA encoding` properties.
- SBOL 1.1 `SequenceAnnotation` objects with `bioStart` and `bioEnd` properties map to SBOL 2.x `SequenceAnnotation` objects that contain `Range` objects.
- SBOL 1.1 `SequenceAnnotation` objects that lack `bioStart` and `bioEnd` properties map to an SBOL 2.x `SequenceFeature` objects that contain `GenericLocation` objects.
- Each SBOL 1.1 `SequenceAnnotation` also maps to an SBOL 2.x `Component`, which represents the instantiation or usage of the appropriate `ComponentDefinition`.
- Each SBOL 1.1 `precedes` property maps to an SBOL 2.x `SequenceConstraint` that specifies a `precedes restriction` property.

### 10.2 Mapping between SBOL 2 and SBOL 3

The base classes of `Identified` and `TopLevel` vary in the following ways between SBOL 2.x and SBOL 3.x:

- SBOL 3.x uses IRIs while SBOL 2.x uses URIs, which are a strict subset of IRIs. In practice, however, many existing SBOL 2 tools actually provide support for IRIs and not just URIs. Accordingly, conversion from SBOL 3.x to SBOL 2.x SHOULD map all IRIs to URIs and conversion from SBOL 2.x to SBOL 3.x MAY convert escaped unicode characters into non-escaped characters in an IRI.
- The SBOL 2.x `Identified` property `persistentIdentity` maps to the SBOL 3.x `identity` property. The `version` property does not exist in SBOL 3.x, but SHOULD be retained through conversion to support conversion back to SBOL 2.x.
- When SBOL 3.x `Identified` object is converted to SBOL 2.x, if its `identity` is a URL, then the `identity` of the SBOL 2.x object SHOULD be constructed as `[SBOL3 identity]/[SBOL2 version]`. If the object does not have an SBOL2 `version` property, then its `version` SHOULD default to 1.
- The SBOL 3.x `TopLevel` property `hasNamespace` does not exist in SBOL 2, and cannot be inferred from an SBOL 2 URI. When converting from SBOL 3.x to SBOL 2.x, the `hasNamespace` property SHOULD be retained to support conversion back to SBOL 3.x.

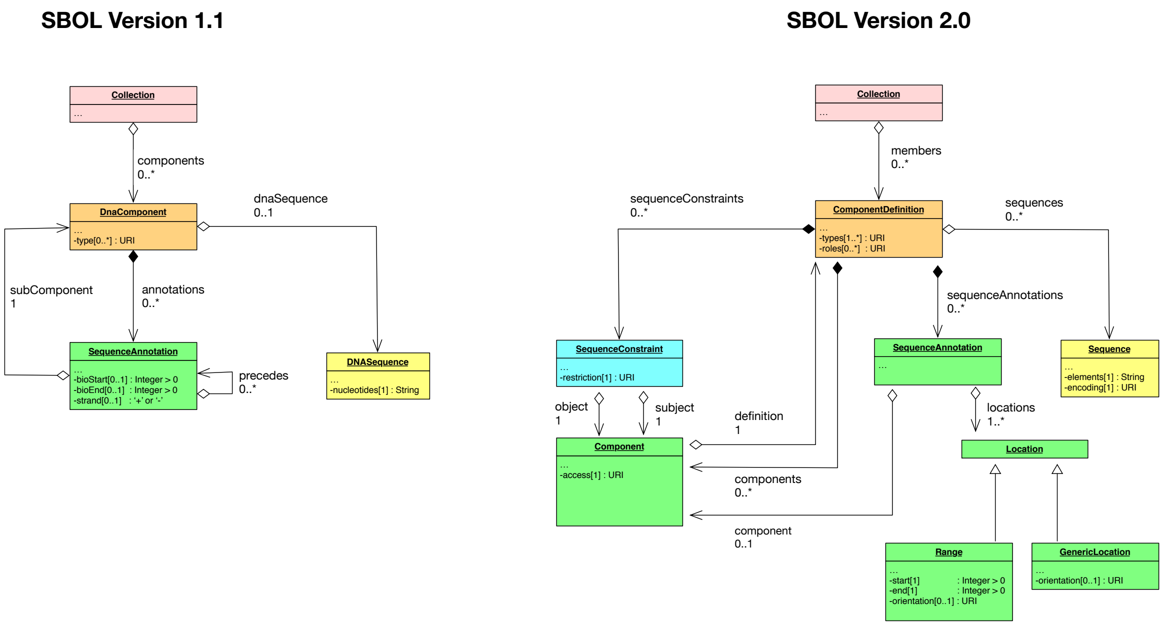

Figure 25: The mapping from the SBOL 1.1 data model to the SBOL 2.x data model, indicating corresponding classes/properties by color.

Figure 26 depicts the mapping of SBOL 2.3 classes to SBOL 3.x classes, indicating corresponding classes/properties by color. The SBOL 2.x `Attachment`, `CombinatorialDerivation`, `ExperimentalData`, `Experiment`, `Implementation`, `Model`, `Participation`,
`Sequence`, and `VariableFeature` classes are omitted or abstracted, since they are essentially unchanged in SBOL 3.x except for the following minor changes:

- In `Sequence`, the `encoding` property values map according to Table 18.
- The SBOL 2.x `VariableComponent` class has been renamed `VariableFeature`.
- In `VariableComponent`, the SBOL 2.x `operator` property maps to the SBOL 3.x `cardinality` property.
- In `VariableComponent`, the `variantMeasure` property has been added, which does not exist in SBOL 2.x.
- In `Experiment`, the SBOL 2.x `experimentalData` property maps to the SBOL 3.x `member` property.
- In `Location`, the SBOL 2.x `sequence` property maps to an SBOL 3.x `hasSequence` property. If there `sequence` property was not set, then the `hasSequence` property is set to one of the values of the `sequences` property of the `ComponentDefinition` that contained the SBOL 2.x `Location`. If there is more than one value for `sequences`, behavior is left deliberately unspecified, and is allowed to be considered an error condition.

The mapping from SBOL 2.x to SBOL 3.x proceeds as follows:

- SBOL 2.x `ComponentDefinition` objects map to SBOL 3.x `Component` objects. The `type` property is mapped according to Table 19.
- SBOL 2.x `ModuleDefinition` objects map to SBOL 3.x `Component` objects with a `type` of `SBO:0000241` (functional entity)
- Every `FunctionalComponent` in an SBOL 2.x `ModuleDefinition` with a "direction" property that is not "none" is listed in the `Interface` of its SBOL 3.x `Component`. The mapping from direction to interface properties is: "in"–>"inputs", "out"–>"outputs", "inout" –> "nondirectional". Finally, every Component with "access"="public" and "direction"="none" is listed as "nondirectional" in the Interface.

- Every `Component` in an SBOL 2.x `ComponentDefinition` with "access"="public" is listed as "nondirectional" in the `Interface` of its SBOL 3.x `Component`.
- SBOL 2.x `Component`, `Module`, and `FunctionalComponent` objects map to SBOL 3.x `SubComponent` objects
- SBOL 2.x `SequenceAnnotation` objects map to SBOL 3.x `SequenceFeature` objects if they do not have a `component`. If they do have a `component`, their locations are added to the corresponding SBOL3 `SubComponent`.
- SBOL 2.x `SequenceConstraint` objects map to SBOL 3.x `Constraint` objects
- SBOL 2.x `MapsTo` objects are converted by transforming each `MapsTo` into two SBOL 3.x objects: a `ComponentReference` and a `Constraint`.
  - For the `ComponentReference`, the `inChildOf` attribute of this `ComponentReference` attribute references the object that has the `MapsTo` as a child, and the `refersTo` attribute references the object referred by the `remote` attribute from the `MapsTo` object.
  - The `Constraint` links this `ComponentReference` and the `SubComponent` referred to be the `local` attribute from the `MapsTo` object. The property values of the `Constraint` depend on the value of the `refinement` value for the `MapsTo` object:
    - If the refinement is `useRemote`, then the `restriction` is `replaces`, the `subject` is the `ComponentReference` and the `object` is the `SubComponent`.
    - If the refinement is `useLocal`, then the `restriction` is `replaces`, the `subject` is the `SubComponent` and the `object` is the `ComponentReference`.
    - If the refinement is `verifyIdentical`, then the `restriction` is `verifyIdentical`, the `subject` is the `ComponentReference` and the `object` is the `SubComponent`.
    - The `merge` `refinement` was never well defined and rarely if ever used, so it has been removed from SBOL 3.x. If a `merge` is encountered, it SHOULD be handled as a `useRemote`.
  - As an OPTIONAL optimization, if the `SubComponent` referred to by the `local` property of the `MapsTo` is a “placeholder” with no significant content apart from its `MapsTo` relationships, then it may be eliminated, all objects that pointed to it can point directly to the new `ComponentReference` instead, and all transitive constraints using it as a bridge reduced to link the endpoints directly.

| SBOL 2.x Type | SBOL 3.x Type |
|---|---|
| http://www.chem.qmul.ac.uk/iubmb/misc/naseq.html | https://identifiers.org/edam:format_1207 |
| http://www.chem.qmul.ac.uk/iupac/AminoAcid/ | https://identifiers.org/edam:format_1208 |
| http://www.opensmiles.org/opensmiles.html | https://identifiers.org/edam:format_1196 |

Table 18: Mapping of `Sequence encoding` values from SBOL2 to SBOL3

| SBOL 2.x Type | SBOL 3.x Type |
|---|---|
| http://www.biopax.org/release/biopax-level3.owl#Dna | https://identifiers.org/SBO:0000251 (DNA) |
| http://www.biopax.org/release/biopax-level3.owl#DnaRegion | https://identifiers.org/SBO:0000251 (DNA) |
| http://www.biopax.org/release/biopax-level3.owl#Rna | https://identifiers.org/SBO:0000250 (RNA) |
| http://www.biopax.org/release/biopax-level3.owl#RnaRegion | https://identifiers.org/SBO:0000250 (RNA) |
| http://www.biopax.org/release/biopax-level3.owl#Protein | https://identifiers.org/SBO:0000252 (Protein) |
| http://www.biopax.org/release/biopax-level3.owl#SmallMolecule | https://identifiers.org/SBO:0000247 (Simple Chemical) |
| http://www.biopax.org/release/biopax-level3.owl#Complex | https://identifiers.org/SBO:0000253 (Non-covalent Complex) |

Table 19: Mapping of SBOL2 `ComponentDefinition` types to SBOL3 `Component` types

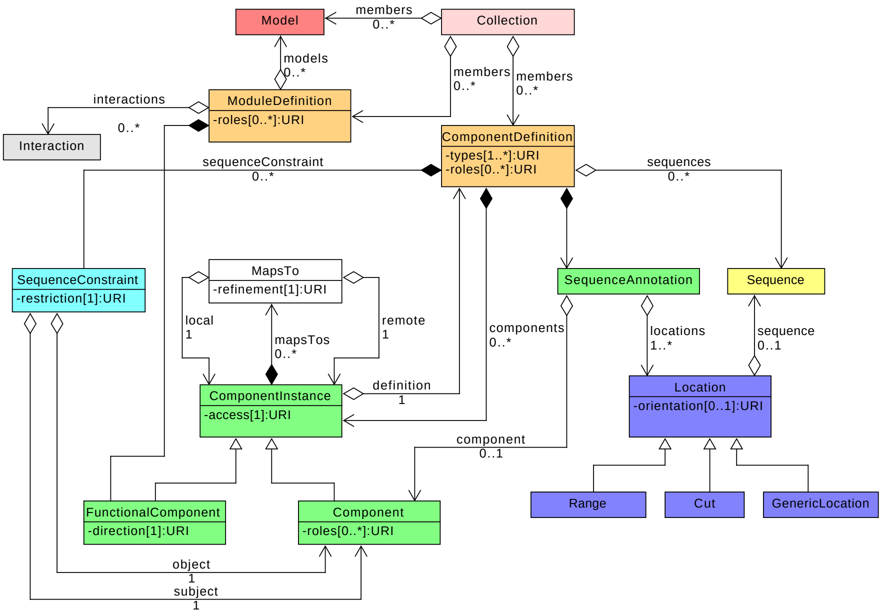

(a) SBOL 2.3

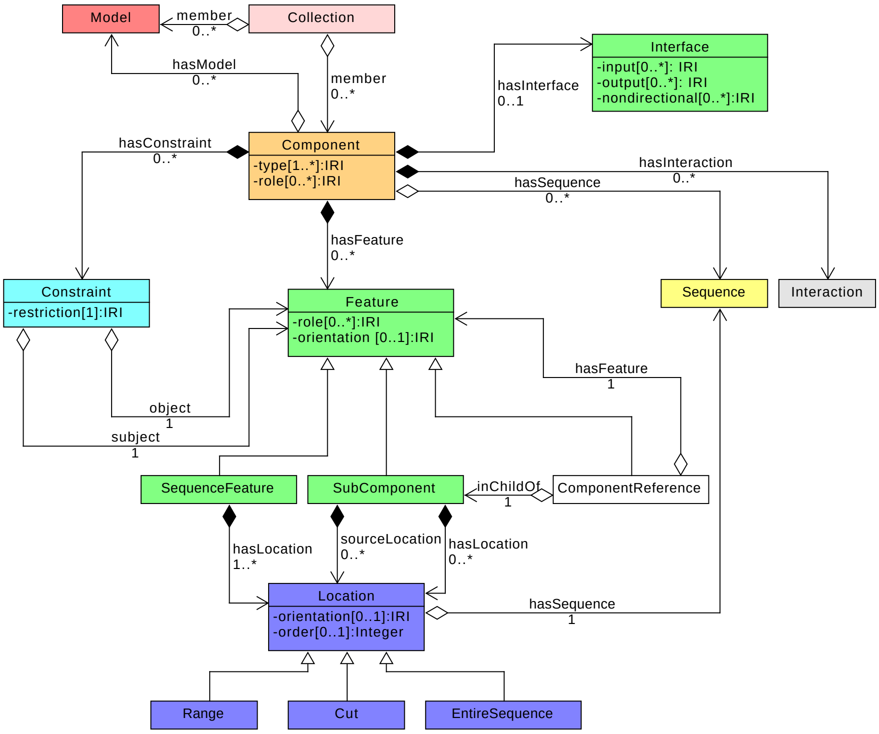

(b) SBOL 3.x

Figure 26: The mapping from the SBOL 2.3 data model to the SBOL 3.x data model, indicating corresponding classes/properties by color.

## A Complementary Standards

Here we discuss two complementary standards that have been adapted for use as part of SBOL representation, following the pattern for extension of SBOL described in Section Section 6.11. In both cases, the extension uses the pattern in which object from another ontology are also assigned to either the SBOL `Identified` or `TopLevel` types. Note that this means that the object receives both an `rdf:type` for the SBOL class and also an `rdf:type` in their own namespace.

### A.1 Adding Provenance with PROV-O

The PROV-O ontology (`https://www.w3.org/ns/prov#`) defines a complementary data model that is leveraged by SBOL to describe provenance. Provenance is central to a range of workflow management, quality control, and attribution tasks within the Synthetic Biology design process. Tracking attribution and derivation of one resource from another is paramount for managing intellectual property purposes. Source designs are often modified in systematic ways to generate derived designs, for example, by applying codon optimization or systematically removing all of a class of restriction enzyme sites. Documenting the transformation used, and any associated parameters, makes this explicit and potentially allows the process to be reproduced systematically. If a design has been used within other designs, and is later found to be defective, it is paramount that all uses of it, including uses of edited versions of the design, can be identified, and ideally replaced with a non-defective alternative. When importing data from external sources, it is important not only to attribute the original source (for example, GenBank), but also the tool used to perform the import, as this may have made arbitrary choices as to how to represent the source knowledge as SBOL. All these activities have in common that it is necessary to track what resource, and what transformation process was applied by whom to derive an SBOL design.

This section describes a minimal subset of PROV-O terms and classes that may be used by SBOL tools to support representation of provenance[1], and how it has been adapted for use with SBOL by assigning PROV-O classes to SBOL `Identified` or `TopLevel` types per Section Section 6.11. Although the full-set of PROV-O terms can be used in SBOL documents, a subset of PROV-O is adopted as a best practice. It is advised that SBOL tools should at least understand this subset, defined in Figure 27. Providers of provenance information are free to make use of more of PROV-O than is described here. It is acceptable for tools that understand more than this subset to use as much as they are able. Tools that only understand this subset must treat any additional data as annotations. Tools that are not aware of SBOL provenance at all MUST maintain and provide access to this information as annotations. This specification does not state what the newly added properties must point to. As long as they are resources that are consistent with the PROV-O property domains, they are legal. For example, a `Component` may be derived from another `Component`, but it would probably not make sense for it to be derived from a `Collection`.

The most basic and general type of provenance relationship can be represented using the `prov:wasDerivedFrom` property. This relationship describes derivation of an SBOL entity from another. Any `Identified` object may be annotated with this property. More specific provenance relationships can also be defined using PROV-O, such as `prov:wasGeneratedBy`. Generation of a new object is defined by the W3C PROV-O specification as follows:

> ...the completion of production of a new entity by an activity. This entity did not exist before generation and becomes available for usage after this generation.

These relationships are leveraged in SBOL tooling for describing multi-stage synthetic biology workflows.

Synthetic biology workflows may involve multiple stages, multiple users, multiple organizations, and interdisciplinary collaborations. These workflows can be described using four core PROV-O classes: `prov:Entity`, `prov:Activity`, `prov:Agent`, and `prov:Plan`. Any SBOL `Identified` object can implicitly act as an instance of PROV-O's `prov:Entity` class. Workflow histories (retrospective provenance) and workflow specifications (prospective provenance) can be described in SBOL using `prov:Activity` objects to link `Identified` objects into workflows.

[1] We thank Dr Paolo Missier from the School of Computing Science, Newcastle University for discussions regarding the use of PROV-O.

An prov:Agent (for example a software or a person) runs an prov:Activity according to a prov:Plan to generate new entities. Resources representing prov:Agent, prov:Activity and prov:Plan classes should be handled as TopLevel, whilst prov:Usage and prov:Association resources should be treated as child Identified objects within their parent prov:Activity objects.

A design-build-test-learn SBOL ontology has been adopted for use with PROV-O classes (see Table 20). The terms *design*, *build*, *test*, and *learn* provide a high level workflow abstraction that allows tool-builders to quickly search for and isolate provenance histories relevant to their domain, while keeping track of the flow of data between different users working in different domains of synthetic biology. These terms SHOULD BE used on the type property of the prov:Activity class. (Note that this property is a special property added by the SBOL specification, and is not part of the original PROV-O specification.) Additionally, these terms SHOULD BE used in the prov:hadRole properties on prov:Usage to qualify how the referenced prov:entity is used by the parent prov:Activity.

| Activity Type | URL | Description |
|---|---|---|
| Design | http://sbols.org/v3#design | Design describes the process by which a conceptual representation of an engineer's imagined and intended design for a biological system is created or derived. |
| Build | http://sbols.org/v3#build | Build describes the process by which a biological construct, sample, or clone is implemented in the laboratory. |
| Test | http://sbols.org/v3#test | Test describes the process of performing experimental measurements to characterize a synthetic biological construct. |
| Learn | http://sbols.org/v3#learn | Learn describes the process of analyzing experimental measurements to produce a new entity that represents biological knowledge. |

Table 20: Synthetic biology workflow ontology

Logical constraints are placed on the order in which different types of prov:Activitys are chained into design-build-test-learn workflows. These rules additionally place constraints on the types of objects that may be used as inputs for a particular type of prov:Activity. For example, a *design* prov:Usage may be used as an input for either a *design* or *build* prov:Activity but SHOULD NOT be used as an input for a *test* prov:Activity. An example of how these terms are used is provided in Figure 28. The ordering of stages and constraints on referred object type are given in Table 21.

| Stage | Preceding Stage | Referred Object Type |
|---|---|---|
| http://sbols.org/v3#design | http://sbols.org/v3#learn | TopLevel other than Implementation |
| http://sbols.org/v3#build | http://sbols.org/v3#design | Implementation |
| http://sbols.org/v3#test | http://sbols.org/v3#build | ExperimentalData |
| http://sbols.org/v3#learn | http://sbols.org/v3#test | Identified other than Implementation |

Table 21: Ordering of design-build-test-learn stages, and the types of objects RECOMMENDED to be associated with them.

In addition to the design-build-test-learn terms, users may also wish to include more specific terms to specify how SBOL objects are used in-house in their own recipes, protocols, or computational analyses. In fact, it is expected that the SBOL workflow ontology will be expanded over time, as users experiment with and develop their own custom ontologies. For now, however, it is RECOMMENDED that SBOL tools also include the high-level terms in Table 20 to support data exchange across interdisciplinary boundaries.

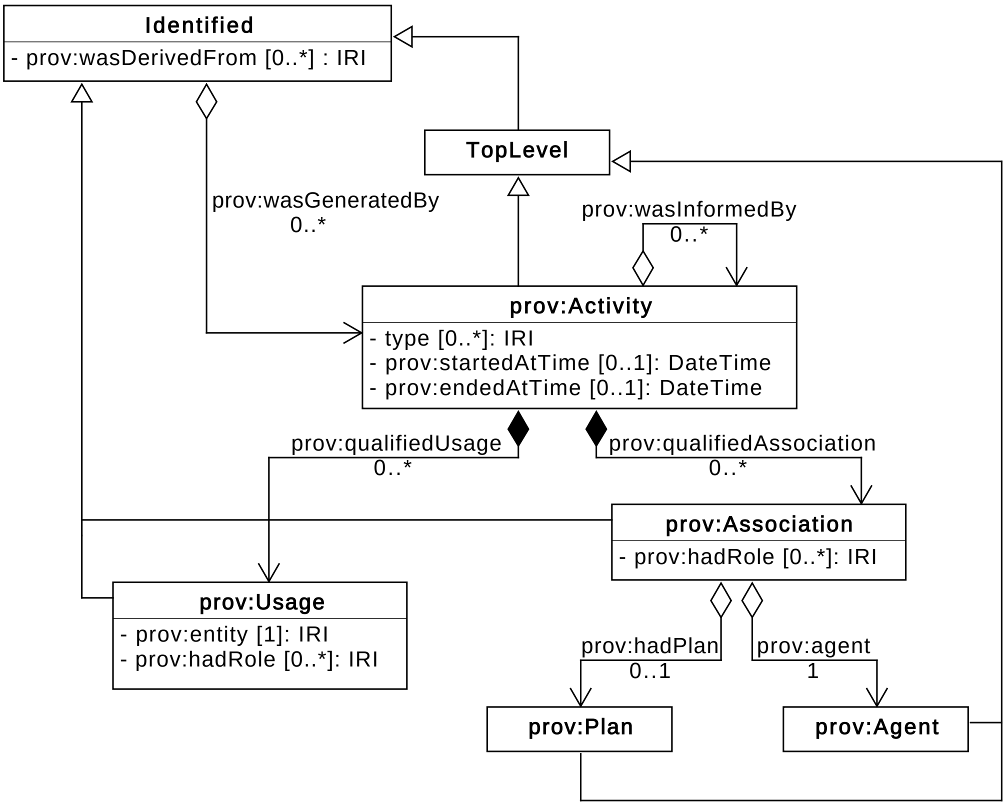

Figure 27: Relationships between SBOL and PROV-O classes. The PROV-O classes `prov:Activity`, `prov:Plan`, and `prov:Agent` all derive from `TopLevel` in the context of the SBOL data model.

### A.1.1 prov:Activity

A generated `prov:Entity` is linked through a `prov:wasGeneratedBy` relationship to an `prov:Activity`, which is used to describe how different `prov:Agent`s and other entities were used. An `prov:Activity` is linked through a `prov:qualifiedAssociation` to `prov:Association`s, to describe the role of agents, and is linked through `prov:qualifiedUsage` to `prov:Usage`s to describe the role of other entities used as part of the activity. Moreover, each `prov:Activity` includes optional `prov:startedAtTime` and `prov:endedAtTime` properties. When using `prov:Activity` to capture how an entity was derived, it is expected that any additional information needed will be attached as annotations. This may include software settings or textual notes. Activities can also be linked together using the `prov:wasInformedBy` relationship to provide dependency without explicitly specifying start and end times.

##### *The* `type` *property*

An `prov:Activity` MAY have one or more `type` properties, each of type `IRI` that explicitly specifies the type of the provenance `prov:Activity` in more detail. If specified, it is RECOMMENDED that at least one `type` property refers to a URL from Table 20.

##### *The* `prov:startedAtTime` *property*

The `prov:startedAtTime` property is OPTIONAL and contains a DateTime (see Section 7.7) value, indicating when the activity started. If this property is present, then the `prov:endedAtTime` property is REQUIRED.

##### *The `prov:endedAtTime` property*

The `prov:endedAtTime` property is OPTIONAL and contains a DateTime (see Section 7.7) value, indicating when the activity ended.

##### *The `prov:qualifiedAssociation` property*

An `prov:Activity` MAY have one or more `prov:qualifiedAssociation` properties, each of type `IRI` that refers to an `prov:Association` object.

##### *The `prov:qualifiedUsage` property*

An `prov:Activity` MAY have one or more `prov:qualifiedUsage` properties, each of type `IRI` that refers to an `prov:Usage` object.

##### *The `prov:wasInformedBy` property*

An `prov:Activity` MAY have one or more `prov:wasInformedBy` properties, each of type `IRI` that refers to another `prov:Activity` object.

#### A.1.2 prov:Usage

How different entities are used in an `prov:Activity` is specified with the `prov:Usage` class, which is linked from an `prov:Activity` through the `prov:Usage` relationship. A `prov:Usage` is then linked to an `prov:Entity` through the `prov:entity` property `IRI` and the `prov:hadRole` property species how the `prov:Entity` is used. When the `prov:wasDerivedFrom` property is used together with the full provenance described here, the entity pointed at by the `prov:wasDerivedFrom` property MUST be included in a `prov:Usage`.

##### *The `prov:entity` property*

The `prov:entity` property is REQUIRED and MUST contain a `IRI` which MAY refer to an `Identified` object.

##### *The `prov:hadRole` property*

An `prov:Usage` MAY have one or more `prov:hadRole` properties, each of type `IRI` that refers to particular term(s) describing the usage of an `prov:Entity` referenced by the `prov:entity` property. Recommended terms that are defined in Table 20 can be used to indicate how the referenced `prov:Entity` is being used in this `prov:Activity`.

#### A.1.3 prov:Association

An `prov:Association` is linked to an `prov:Agent` through the `prov:agent` relationship. The `prov:Association` includes the `prov:hadRole` property to qualify the role of the `prov:Agent` in the `prov:Activity`.

##### *The `prov:agent` property*

The `prov:agent` property is REQUIRED and MUST contain a `IRI` that refers to an `prov:Agent` object.

##### *The `prov:hadRole` property*

An `prov:Association` MAY have one or more `prov:hadRole` properties, each of type `IRI` that refers to particular term(s) that describes the role of the `prov:Agent` in the parent `prov:Activity`.

##### *The `prov:hadPlan` property*

The `prov:hadPlan` property is OPTIONAL and contains a `IRI` that refers to a `prov:Plan`.

#### A.1.4 prov:Plan

The `prov:Plan` entity can be used as a place holder to describe the steps (for example scripts or lab protocols) taken when an `prov:Agent` is used in a particular `prov:Activity`.

#### A.1.5 prov:Agent

Examples of agents are a person, organization, or software tool. These agents should be annotated with additional information, such as software version, needed to be able to run the same `prov:Activity` again.

##### Example - Codon optimization

Codon optimization is an example of where provenance properties can be applied. The relationship between an original CDS and the codon-optimized version could simply be represented using the `prov:wasDerivedFrom` predicate, in a light-weight form. With more comprehensive use of the PROV ontology, the codon optimization can be represented as an `prov:Activity`. This `prov:Activity` can then include additional information, such as the `prov:Agent` responsible (in this case, codon-optimizing software), and additional parameters.

##### Example - Deriving strains

Bacterial strains are often derived from other strains through modifications such as gene knockouts or mutations. For example, the *Bacillus subtilis* 168 strain was derived from the NCIMB3610 strain in the 1940s through x-radiation. *B. subtilis* 168 is a laboratory strain and has several advantages as a model organism in synthetic biology. The relationship between the original strain and the 168 strain can be represented using the `prov:wasDerivedFrom` predicate or, more comprehensively, with an `prov:Activity` describing the protocols used.

##### Example - Design-build-test-learn Workflow

Figure 28 illustrates one complete iteration through a design-build-test-learn cycle. The workflow begins with a `Model` which describes the hypothesized behavior of a biological device. Using a computational tool, a new Design (`Component`) is composed from biological parts, which links back to its `Model`. A genetic construct is then produced in the laboratory via an assembly protocol, and this biological sample is represented by a Build (`Implementation`). Once constructed, the Build is then characterized in the laboratory using an automated measurement protocol on a Tecan plate reader, thus generating Test data (represented by an `ExperimentalData`). Finally, a new `Model` is derived from these data using a fitting algorithm implemented in the Python programming language. The final `Model` may not match the beginning `Model`, as the observed behavior may not match the prediction.

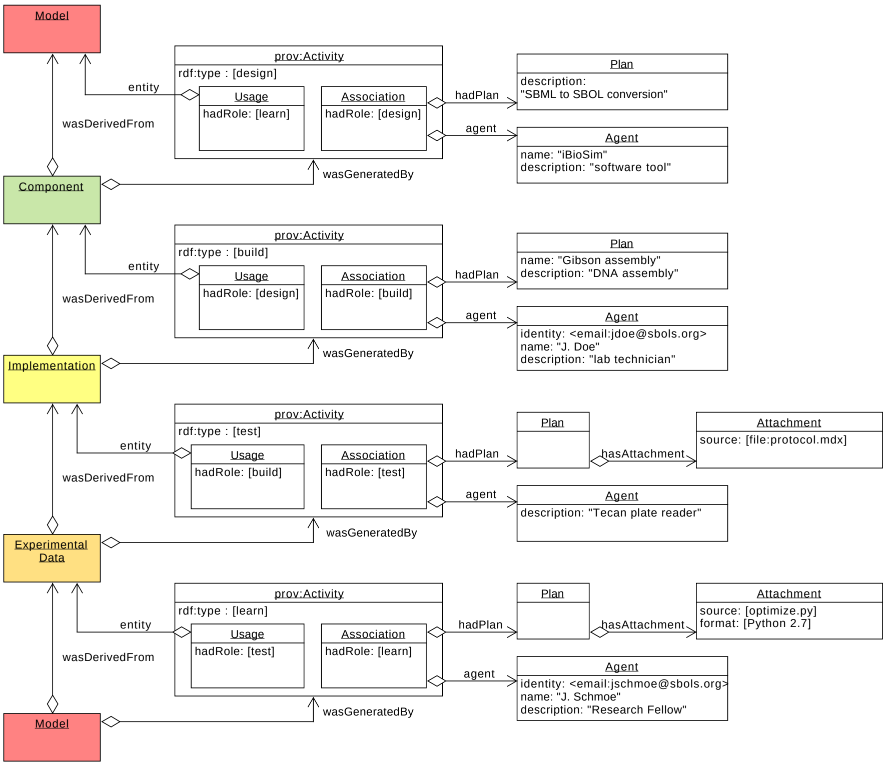

Figure 28: An example data structure representing an idealized workflow for model-based design.

***Example - Combinatorial Derivation***

As specified in the description of `CombinatorialDerivation`, provenance can be used to link each generated `Component` (or `Collection` thereof) back to the source form which it was derived. In particular, each derived design links with `prov:wasDerivedFrom` to the `CombinatorialDerivation` that it was derived from. Also, each `SubComponent` has a `prov:wasDerivedFrom` linking it to the `SubComponent` within the `template` that it is derived from. The advantage of these provenance links is that they provide sufficient information to validate that this derived design has been properly derived from the specified `CombinatorialDerivation`s.

### A.2 Adding Measures/Parameters with OM

There are at least two well-established cases for including measures/parameters and their associated units in SBOL design specifications. These use cases are the specification of genetic circuit designs and their associated parameters (such as rates of transcription) and the specification of environmental conditions for biological system designs (such as growth media concentrations and temperatures). In the first use case, parameters are necessary to enable the generation of quantitative models of circuit behavior from circuit design specifications. In the second use case, measures are necessary to define experimental conditions and enable the analysis of system behavior or characterization with respect to environmental context.

The Ontology of Units of Measure (OM) (`http://www.ontology-of-units-of-measure.org/resource/om-2`) already defines a data model for representing measures and their associated units. Here, a subset of OM is adopted by SBOL to describe these concepts for biological design specifications, by assigning PROV-O classes to SBOL `Identified` or `TopLevel` types per Section Section 6.11. As shown in Figure 29, SBOL leverages three of the base classes defined by the OM: `om:Measure`, `om:Unit` and `om:Prefix`. A `om:Measure` links a numerical value to a `om:Unit`, which may or may not have a `om:Prefix` (e.g. centi, milli, micro, etc.). As these classes are adopted by SBOL, `om:Measure` is treated as a subclass of `Identified`, while `om:Unit` and `om:Prefix` are treated as subclasses of `TopLevel`. In addition, SBOL adopts the following OM `om:Unit` subclasses: `om:SingularUnit`, `om:CompoundUnit`, `om:UnitMultiplication`, `om:UnitDivision`, `om:UnitExponentiation`, and `om:PrefixedUnit`. Lastly, SBOL adopts the following `om:Prefix` subclasses from OM: `om:SIPrefix` and `om:BinaryPrefix`.

OM also provides a large number of predefined `om:Unit` instances, so in most cases there is no need to create anything other than `om:Measure` objects that refer to pre-existing instances. This can simplify the comparison and interpretation of units, so for this reason, a pre-existing `om:Unit` instance SHOULD be used whenever one is applicable. If a unit does not already exist in the ontology, however, then the `om:Unit` subclasses MAY be used to create new units.

SBOL-compliant tools are allowed to read, write, and modify data belonging to OM classes other than those described here, but this specification does not provide any guidance for the interpretation or use of these data in the context of SBOL.

#### A.2.1 om:Measure

The purpose of the `om:Measure` class is to link a numerical value to a `om:Unit`.

***The `om:hasNumericalValue` property***

The `om:hasNumericalValue` property is REQUIRED and MUST contain a single xsd:float.

***The `om:hasUnit` property***

The `om:hasUnit` property is REQUIRED and MUST contain a `IRI` that refers to a `om:Unit`. The OM provides `IRI`s for many existing instances of the `om:Unit` class for reference (for example, `http://www.ontology-of-units-of-measure.org/resource/om-2/gramPerLitre`).

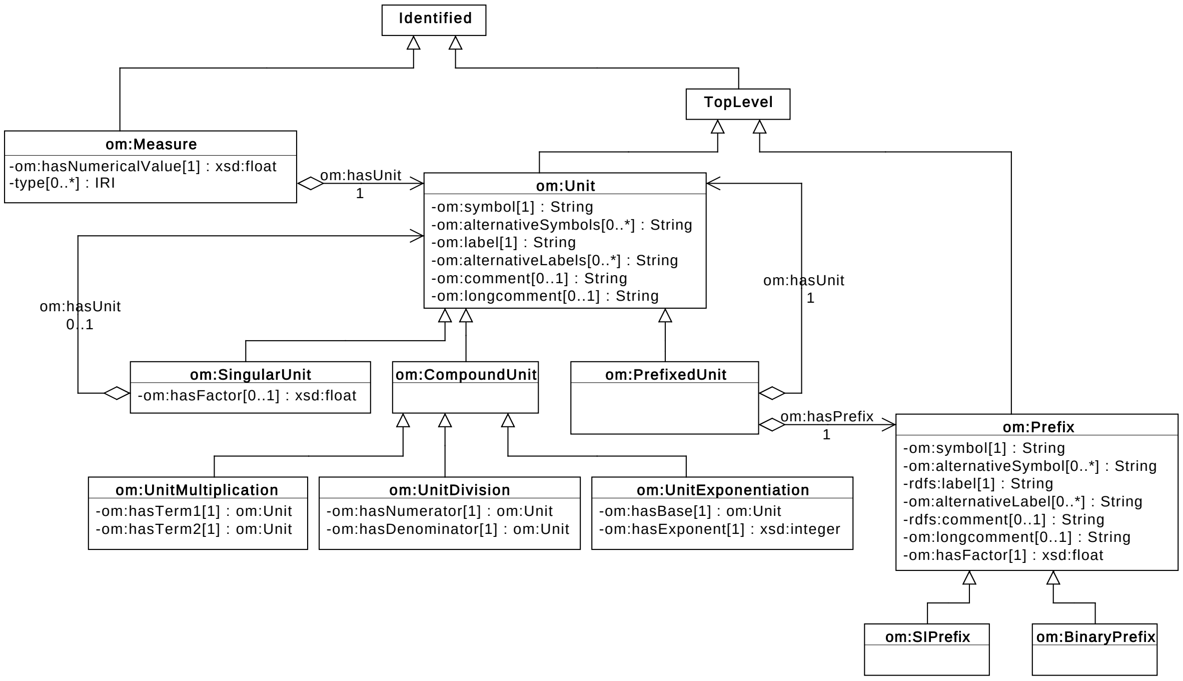

Figure 29: OM classes adopted by SBOL and their subclass relationships to `Identified` and `TopLevel`

##### *The `type` property*

A `om:Measure` MAY have one or more `type` properties, each is of type `IRI`. It is RECOMMENDED that one of these `IRI`s identify a term from the Systems Description Parameter branch of the Systems Biology Ontology (SBO) (`http://www.ebi.ac.uk/sbo/main/`). This `type` property of the `om:Measure` class is not specified in the OM and is added by SBOL to describe different types of parameters (for example, rate of reaction is identified by the SBO term `http://identifiers.org/SBO:0000612`).

#### A.2.2 om:Unit

As adopted by SBOL, `om:Unit` is an abstract class that is extended by other classes to describe units of measure using a shared set of properties.

##### *The `om:symbol` property*

The `om:symbol` property is REQUIRED and MUST contain a `String`. This `String` is commonly used to abbreviate the unit of measure's name. For example, the unit of measure named "gram per liter" is commonly abbreviated using the `String` "g/l".

##### *The `om:alternativeSymbols` property*

The `om:alternativeSymbols` property is OPTIONAL and MAY contain a set of `String`s. This property can be used to specify alternative abbreviations other than that specified using the `om:symbol` property.

##### *The `om:label` property*

The `om:label` property is REQUIRED and MUST contain a `String`. This `String` is a common name for the unit of measure and SHOULD be identical to any `String` contained by the `name` property inherited from `Identified`.

##### The om:alternativeLabels property

The om:alternativeLabels property is OPTIONAL and MAY contain a set of Strings. This property can be used to specify alternative common names other than that specified using the om:label property.

##### The om:comment property

The om:comment property is OPTIONAL and MAY contain a String. This String is a description of the unit of measure and SHOULD be identical to any String contained by the description property inherited from Identified.

##### The om:longcomment property

The om:longcomment property is OPTIONAL and MAY contain a String. This String is a long description of the unit of measure and SHOULD be longer than any String contained by the om:comment property.

#### A.2.3 om:SingularUnit

The purpose of the om:SingularUnit class is to describe a unit of measure that is not explicitly represented as a combination of multiple units, but could be equivalent to such a representation. For example, a joule is considered to be a om:SingularUnit, but it is equivalent to the multiplication of a newton and a meter.

##### The om:hasUnit property

The om:hasUnit is OPTIONAL and MAY contain a IRI. This IRI MUST refer to another om:Unit. The om:hasUnit propery can be used in conjunction with the om:hasFactor property to specify whether a om:SingularUnit is equivalent to another om:Unit multiplied by a factor. For example, an angstrom is equivalent to $10^{-10}$ meters.

##### The om:hasFactor property

The om:hasFactor property is OPTIONAL and MAY contain a xsd:float. If the om:hasFactor property of a om:SingularUnit is non-empty, then its om:hasUnit property SHOULD also be non-empty.

#### A.2.4 om:CompoundUnit

As adopted by SBOL, om:CompoundUnit is an abstract class that is extended by other classes to describe units of measure that can be represented as combinations of multiple other units of measure.

#### A.2.5 om:UnitMultiplication

The purpose of the om:UnitMultiplication class is to describe a unit of measure that is the multiplication of two other units of measure.

##### The om:hasTerm1 property

The om:hasTerm1 property is REQUIRED and MUST contain a IRI that refers to another om:Unit. This om:Unit is the first multiplication term.

##### The om:hasTerm2 property

The om:hasTerm2 property is REQUIRED and MUST contain a IRI that refers to another om:Unit. This om:Unit is the second multiplication term. It is okay if the om:Unit referred to by om:hasTerm1 is the same as that referred to by om:hasTerm2.

### A.2.6 om:UnitDivision

The purpose of the om:UnitDivision class is to describe a unit of measure that is the division of one unit of measure by another.

##### *The om:hasNumerator property*

The om:hasNumerator property is REQUIRED and MUST contain a IRI that refers to another om:Unit.

##### *The om:hasDenominator property*

The om:hasDenominator property is REQUIRED and MUST contain a IRI that refers to another om:Unit.

### A.2.7 om:UnitExponentiation

The purpose of the om:UnitExponentiation class is to describe a unit of measure that is raised to an integer power.

##### *The om:hasBase property*

The om:hasBase property is REQUIRED and MUST contain a IRI that refers to another om:Unit.

##### *The om:hasExponent property*

The om:hasExponent property is REQUIRED and MUST contain an xsd:integer.

### A.2.8 om:PrefixedUnit

The purpose of the om:PrefixedUnit class is to describe a unit of measure that is the multiplication of another unit of measure and a factor represented by a standard prefix such as "milli," "centi," "kilo," etc.

##### *The om:hasUnit property*

The om:hasUnit property is REQUIRED and MUST contain a IRI that refers to another om:Unit.

##### *The om:hasPrefix property*

The om:hasPrefix property is REQUIRED and MUST contain a IRI that refers to a om:Prefix.

### A.2.9 om:Prefix

As adopted by SBOL, om:Prefix is an abstract class that is extended by other classes to describe factors that are commonly represented by standard unit prefixes. For example, the factor $10^{-3}$ is represented by the standard unit prefix "milli."

##### *The om:symbol property*

The om:symbol property is REQUIRED and MUST contain a String. This String is commonly used to abbreviate the name of the unit prefix. For example, the String "m" is commonly used to abbreviate the name "milli."

##### *The om:alternativeSymbols property*

The om:alternativeSymbols property is OPTIONAL and MAY contain a set of Strings. This property can be used to specify alternative abbreviations other than that specified using the om:symbol property.

##### *The om:label property*

The om:label property is REQUIRED and MUST contain a String. This String is a common name for the unit prefix and SHOULD be identical to any String contained by the name property inherited from Identified.

##### *The* ***om:alternativeLabels*** *property*

The om:alternativeLabels property is OPTIONAL and MAY contain a set of Strings. This property can be used to specify alternative common names other than that specified using the om:label property.

##### *The* ***om:comment*** *property*

The om:comment property is OPTIONAL and MAY contain a String. This String is a description of the unit prefix and SHOULD be identical to any String contained by the description property inherited from Identified.

##### *The* ***om:longcomment*** *property*

The om:longcomment property is OPTIONAL and MAY contain a String. This String is a long description of the unit of measure and SHOULD be longer than any String contained by the om:comment property.

##### *The* ***om:hasFactor*** *property*

The om:hasFactor property is REQUIRED and MUST contain an xsd:float.

#### A.2.10 om:SIPrefix

The purpose of the om:SIPrefix class is to describe standard SI prefixes such as “milli,” “centi,” “kilo,” etc.

#### A.2.11 om:BinaryPrefix

The purpose of the om:BinaryPrefix class is to describe standard binary prefixes such as “kibi,” “mebi,” “gibi,” etc. These prefixes commonly precede units of information such as “bit” and “byte.”

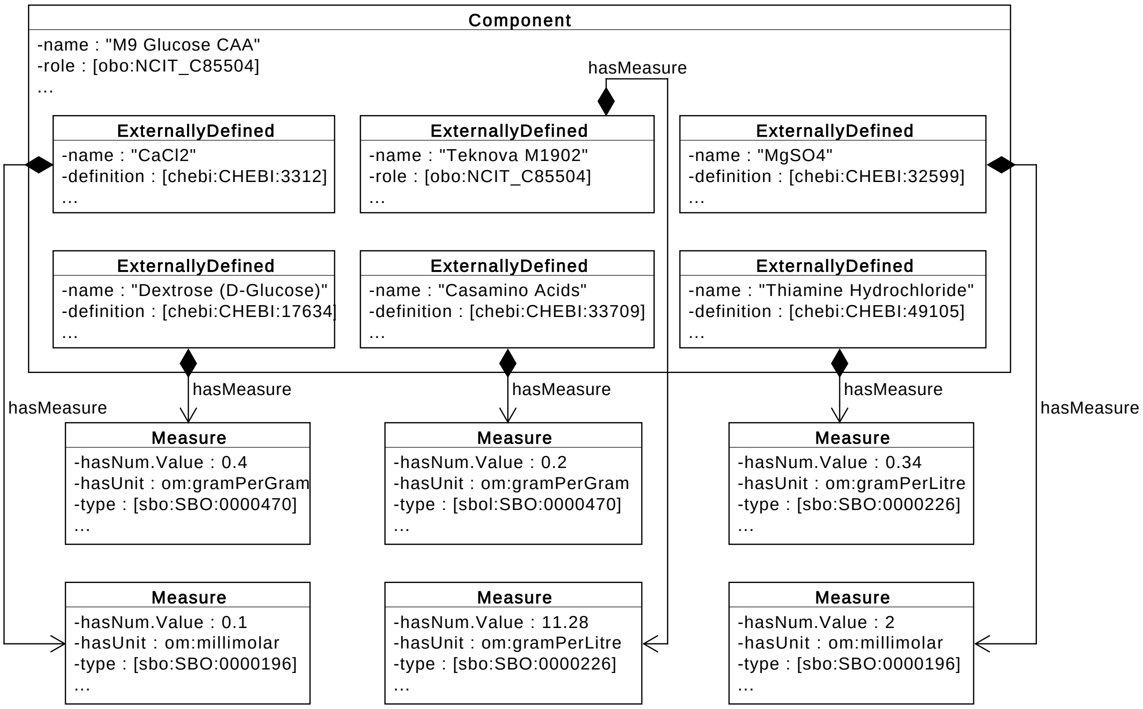

Figure 30: Growth media recipe represented using instances of the om:Measure and om:Unit classes from the OM.

## B Validation Rules

This section summarizes all the conditions that either MUST be or are RECOMMENDED to be true of an SBOL Version 3.0 document. There are different degrees of rule strictness. Rules of the former kind are strict SBOL validation rules—data encoded in SBOL MUST conform to all of them in order to be considered valid. Rules of the latter kind are consistency rules that SBOL data are RECOMMENDED to adhere to as a best practice. To help highlight these differences, we use the following symbols next to the rule numbers:

- ☑ A checked box indicates a strong REQUIRED condition for SBOL conformance. If a SBOL document does not follow this rule, it does not conform to the SBOL specification.
- ● A circle indicates a weak REQUIRED condition for SBOL conformance. While this rule MUST be followed, there are conditions under which it can only be partially checked by a machine (e.g., due to references to data that is not accessible or data with an ambiguous format). Rules of this type SHOULD be checked insofar as is possible given the information available in a SBOL document.
- ★ A star indicates a RECOMMENDED condition for following best practices. This rule is not strictly a matter of SBOL conformance, but its recommendation comes from logical reasoning. If an SBOL document does not follow this rule, it is still valid SBOL, but it might have degraded functionality in some tools.

We also include a fourth type of rule that represents a required condition for SBOL-compliance that cannot be checked by a machine. Therefore, violations of these rules are not expected to be reported as errors by any of the software libraries implementing SBOL 3.0. It is the user's responsibility to make sure that these validation rules are followed.

- ▲ A triangle indicates a weak REQUIRED condition for SBOL conformance. While this rule MUST be followed, it is not possible in practice for a machine to automatically check whether the rule has been followed.

The validation rules listed in the following subsections are all believed to be stated or implied in the rest of this specification document. They are enumerated here for convenience and to provide a "master checklist" for SBOL validation. In case of a conflict between this section and other portions of the specification (though there are believed to be none), this section is considered authoritative for the purpose of determining the validity of an SBOL document.

☞ Not all classes have validation rules specific to that class. For classes whose validation is covered by the rules for all SBOL objects, the type is not explicitly listed below. A range in the validation rules numbers, however, has been reserved in case of future need.

### Rules for SBOL Objects

**sbol3-10101** ● The IRI of an `Identified` object MUST be globally unique.
Reference: Section 5.1 on page 12

**sbol3-10102** ☑ A `TopLevel` URL MUST use the following pattern: `[namespace]/[local]/[displayId]`, where `namespace` and `displayId` are required fragments, and the `local` fragment is an optional relative path.
Reference: Section 5.1 on page 12

**sbol3-10103** ☑ A `TopLevel` object's URL MUST NOT be included as prefix for any other `TopLevel` object.
Reference: Section 5.1 on page 12

**sbol3-10104** ☑ The URL of any child or nested object MUST use the following pattern: `[parent]/[displayId]`, where `parent` is the URL of its parent object. Multiple layers of child objects are allowed, using the same `[parent]/[displayId]` pattern recursively.
Reference: Section 5.1 on page 12

**sbol3-10105** ☑ The SBOL namespace MUST NOT be used for any entities or properties not defined in this specification.
Reference: Section 5.2 on page 12

**sbol3-10106** ☑ An object MUST NOT have `rdfType` properties in the "`http://sbols.org/v3#`" namespace that refer to disjoint classes.
Reference: Section 5.4 on page 13

**sbol3-10107** ★ An object SHOULD have no more than one `rdfType` property in the "`http://sbols.org/v3#`" namespace.
Reference: Section 5.4 on page 13

**sbol3-10108** ★ If an object has a property in the "`http://sbols.org/v3#`" namespace (e.g., `sbol:displayId`, then it SHOULD also have an `rdfType` property in that namespace.
Reference: Section 5.4 on page 13

**sbol3-10109** ☑ An object MUST NOT have properties in the "`http://sbols.org/v3#`" namespace other than those listed for its type or parent types in Table 22.
Reference: Section 5.2 on page 12

**sbol3-10110** ☑ An object MUST have a number of instances of a property that matches the cardinality restrictions listed for that object type and property in Table 23.
Reference: Section 4.2 on page 10

**sbol3-10111** ☑ An object's property values MUST have the type listed for the object type and property in Table 23.
Reference: Section 5.3 on page 12

**sbol3-10112** ☑ Each property of type `IRI` that is listed with a reference type in Table 23 MUST refer to an object of the type listed (child objects).
Reference: Section 5.3 on page 12

**sbol3-10113** ● Each property of type `IRI` that is listed with a reference type in Table 23 MUST refer to an object of the type listed.
Reference: Section 5.3 on page 12

**sbol3-10114** ★ Each property of type `IRI` that is listed with a `TopLevel` reference type in Table 23 SHOULD be able to be dereferenced to obtain an SBOL object.
Reference: Section 5.3 on page 12

Table 22: Allowed object properties in the "`http://sbols.org/v3#`" namespace.

| Class | Parent | SBOL Properties | Reference |
|---|---|---|---|
| `Attachment` | `TopLevel` | `source`, `format`, `size`, `hash`, `hashAlgorithm` | Section 6.10 on page 39 |
| `Collection` | `TopLevel` | `member` | Section 6.9 on page 38 |
| `CombinatorialDerivation` | `TopLevel` | `template`, `strategy`, `hasVariableFeature` | Section 6.5 on page 33 |
| `ComponentReference` | `Feature` | `inChildOf`, `refersTo` | Section 6.4.1.2 on page 24 |
| `Component` | `TopLevel` | `type`, `role`, `hasSequence`, `hasFeature`, `hasInteraction`, `hasConstraint`, `hasModel`, `hasInterface` | Section 6.4 on page 18 |
| `Constraint` | `Identified` | `subject`, `object`, `restriction` | Section 6.4.3 on page 27 |
| `Cut` | `Location` | `at` | Section 6.4.2.2 on page 27 |
| `EntireSequence` | `Location` | | Section 6.4.2.3 on page 27 |
| `ExperimentalData` | `TopLevel` | | Section 6.7 on page 37 |
| `Experiment` | `Collection` | | Section 6.9.1 on page 39 |
| `ExternallyDefined` | `Feature` | `type`, `definition` | Section 6.4.1.4 on page 25 |
| `Feature` | `Identified` | `role`, `orientation` | Section 6.4.1 on page 22 |
| `Identified` | none | `displayId`, `name`, `description`, `hasMeasure` | Section 6.1 on page 15 |
| `Implementation` | `TopLevel` | `built` | Section 6.6 on page 36 |
| `Interaction` | `Identified` | `type`, `hasParticipation` | Section 6.4.4 on page 28 |
| `Interface` | `Identified` | `input`, `output`, `nondirectional` | Section 6.4.5 on page 32 |

*Continued on next page*

Table 22 – *Continued from previous page*

| Class | Parent | SBOL Properties | Reference |
|---|---|---|---|
| LocalSubComponent | Feature | type, hasLocation | Section 6.4.1.3 on page 25 |
| Location | Identified | orientation, order, hasSequence | Section 6.4.2 on page 26 |
| Model | TopLevel | source, language, framework | Section 6.8 on page 37 |
| Participation | Identified | role, participant, higherOrderParticipant | Section 6.4.4.1 on page 31 |
| Range | Location | start, end | Section 6.4.2.1 on page 27 |
| SequenceFeature | Feature | hasLocation | Section 6.4.1.5 on page 25 |
| Sequence | TopLevel | elements, encoding | Section 6.3 on page 16 |
| SubComponent | Feature | roleIntegration, instanceOf, sourceLocation, hasLocation | Section 6.4.1.1 on page 23 |
| TopLevel | Identified | hasNamespace, hasAttachment | Section 6.2 on page 16 |
| VariableFeature | Identified | cardinality, variable, variant, variantCollection, variantDerivation, variantMeasure | Section 6.5.1 on page 34 |
| prov:Activity | TopLevel | type | Section A.1.1 on page 57 |
| prov:Agent | TopLevel | | Section A.1.5 on page 59 |
| prov:Association | Identified | | Section A.1.3 on page 58 |
| prov:Plan | TopLevel | | Section A.1.4 on page 59 |
| prov:Usage | Identified | | Section A.1.2 on page 58 |
| om:BinaryPrefix | om:Prefix | | Section A.2.11 on page 65 |
| om:CompoundUnit | om:Unit | | Section A.2.4 on page 63 |
| om:Measure | Identified | type | Section A.2.1 on page 61 |
| om:PrefixedUnit | om:Unit | | Section A.2.8 on page 64 |
| om:Prefix | TopLevel | | Section A.2.9 on page 64 |
| om:SIPrefix | om:Prefix | | Section A.2.10 on page 65 |
| om:SingularUnit | om:Unit | | Section A.2.3 on page 63 |
| om:UnitDivision | om:CompoundUnit | | Section A.2.6 on page 64 |
| om:UnitExponentiation | om:CompoundUnit | | Section A.2.7 on page 64 |
| om:UnitMultiplication | om:CompoundUnit | | Section A.2.5 on page 63 |
| om:Unit | TopLevel | | Section A.2.2 on page 62 |

Table 23: Cardinality constraints on object properties, their types, and types of referred objects.

| Class | Property | Cardinality | Type | Referred Type | Reference |
|---|---|---|---|---|---|
| Attachment | source | EXACTLY ONE | IRI | — | Section 6.10 on page 39 |
| Attachment | format | ZERO OR ONE | IRI | — | Section 6.10 on page 39 |
| Attachment | hashAlgorithm | ZERO OR ONE | String | — | Section 6.10 on page 39 |
| Attachment | hash | ZERO OR ONE | String | — | Section 6.10 on page 39 |
| Attachment | size | ZERO OR ONE | Long | — | Section 6.10 on page 39 |
| Collection | member | ZERO OR MORE | IRI | TopLevel | Section 6.9 on page 38 |
| CombinatorialDerivation | hasVariableFeature | ZERO OR MORE | IRI | VariableFeature | Section 6.5 on page 33 |
| CombinatorialDerivation | strategy | ZERO OR ONE | IRI | — | Section 6.5 on page 33 |
| CombinatorialDerivation | template | EXACTLY ONE | IRI | Component | Section 6.5 on page 33 |
| ComponentReference | refersTo | EXACTLY ONE | IRI | Feature | Section 6.4.1.2 on page 24 |
| ComponentReference | inChildOf | EXACTLY ONE | IRI | SubComponent | Section 6.4.1.2 on page 24 |
| Component | hasSequence | ZERO OR MORE | IRI | Sequence | Section 6.4 on page 18 |
| Component | role | ZERO OR MORE | IRI | — | Section 6.4 on page 18 |
| Component | type | ONE OR MORE | IRI | — | Section 6.4 on page 18 |
| Component | hasConstraint | ZERO OR MORE | IRI | Constraint | Section 6.4 on page 18 |
| Component | hasFeature | ZERO OR MORE | IRI | Feature | Section 6.4 on page 18 |
| Component | hasInteraction | ZERO OR MORE | IRI | Interaction | Section 6.4 on page 18 |
| Component | hasInterface | ZERO OR MORE | IRI | Interface | Section 6.4 on page 18 |
| Component | hasModel | ZERO OR MORE | IRI | Model | Section 6.4 on page 18 |
| Constraint | object | EXACTLY ONE | IRI | Feature | Section 6.4.3 on page 27 |
| Constraint | restriction | EXACTLY ONE | IRI | — | Section 6.4.3 on page 27 |
| Constraint | subject | EXACTLY ONE | IRI | Feature | Section 6.4.3 on page 27 |
| Cut | at | EXACTLY ONE | Integer | — | Section 6.4.2.2 on page 27 |
| Experiment | member | ZERO OR MORE | IRI | ExperimentalData | Section 6.9 on page 38 |
| ExternallyDefined | definition | EXACTLY ONE | IRI | — | Section 6.4.1.4 on page 25 |
| ExternallyDefined | type | ONE OR MORE | IRI | — | Section 6.4.1.4 on page 25 |
| Feature | orientation | ZERO OR ONE | IRI | — | Section 6.4.1 on page 22 |
| Feature | role | ZERO OR MORE | IRI | — | Section 6.4.1 on page 22 |
| Identified | prov:wasDerivedFrom | ZERO OR MORE | IRI | — | Section 6.1 on page 15 |
| Identified | prov:wasGeneratedBy | ZERO OR MORE | IRI | prov:Activity | Section 6.1 on page 15 |
| Identified | description | ZERO OR ONE | String | — | Section 6.1 on page 15 |
| Identified | displayId | ZERO OR ONE | String | — | Section 6.1 on page 15 |
| Identified | hasMeasure | ZERO OR MORE | IRI | om:Measure | Section 6.1 on page 15 |
| Identified | name | ZERO OR ONE | String | — | Section 6.1 on page 15 |
| Implementation | built | ZERO OR ONE | IRI | Component | Section 6.6 on page 36 |
| Interaction | type | ONE OR MORE | IRI | — | Section 6.4.4 on page 28 |
| Interaction | hasParticipation | ZERO OR MORE | IRI | Participation | Section 6.4.4 on page 28 |
| Interface | input | ZERO OR MORE | IRI | Feature | Section 6.4.5 on page 32 |
| Interface | nondirectional | ZERO OR MORE | IRI | Feature | Section 6.4.5 on page 32 |
| Interface | output | ZERO OR MORE | IRI | Feature | Section 6.4.5 on page 32 |
| LocalSubComponent | hasLocation | ZERO OR MORE | IRI | Location | Section 6.4.1.3 on page 25 |
| LocalSubComponent | type | ONE OR MORE | IRI | — | Section 6.4.1.3 on page 25 |

*Continued on next page*

Table 23 – *Continued from previous page*

| Class | Property | Cardinality | Type | Referred Type | Reference |
|---|---|---|---|---|---|
| Location | orientation | ZERO OR ONE | IRI | — | Section 6.4.2 on page 26 |
| Location | order | ZERO OR ONE | Integer | — | Section 6.4.2 on page 26 |
| Location | hasSequence | EXACTLY ONE | IRI | Sequence | Section 6.4.2 on page 26 |
| Model | source | EXACTLY ONE | IRI | — | Section 6.8 on page 37 |
| Model | framework | EXACTLY ONE | IRI | — | Section 6.8 on page 37 |
| Model | language | EXACTLY ONE | IRI | — | Section 6.8 on page 37 |
| Participation | participant | ZERO OR ONE | IRI | Feature | Section 6.4.4.1 on page 31 |
| Participation | higherOrderParticipant | ZERO OR ONE | IRI | Interaction | Section 6.4.4.1 on page 31 |
| Participation | role | ONE OR MORE | IRI | — | Section 6.4.4.1 on page 31 |
| Range | end | EXACTLY ONE | Integer | — | Section 6.4.2.1 on page 27 |
| Range | start | EXACTLY ONE | Integer | — | Section 6.4.2.1 on page 27 |
| SequenceFeature | hasLocation | ONE OR MORE | IRI | Location | Section 6.4.1.5 on page 25 |
| Sequence | elements | ZERO OR ONE | String | — | Section 6.3 on page 16 |
| Sequence | encoding | ZERO OR ONE | IRI | — | Section 6.3 on page 16 |
| SubComponent | instanceOf | EXACTLY ONE | IRI | Component | Section 6.4.1.1 on page 23 |
| SubComponent | roleIntegration | ZERO OR ONE | IRI | — | Section 6.4.1.1 on page 23 |
| SubComponent | sourceLocation | ZERO OR MORE | IRI | Location | Section 6.4.1.1 on page 23 |
| SubComponent | hasLocation | ZERO OR MORE | IRI | Location | Section 6.4.1.1 on page 23 |
| TopLevel | hasAttachment | ZERO OR MORE | IRI | Attachment | Section 6.2 on page 16 |
| TopLevel | hasNamespace | EXACTLY ONE | URL | — | Section 6.2 on page 16 |
| VariableFeature | cardinality | EXACTLY ONE | IRI | — | Section 6.5.1 on page 34 |
| VariableFeature | variable | EXACTLY ONE | IRI | Feature | Section 6.5.1 on page 34 |
| VariableFeature | variantCollection | ZERO OR MORE | IRI | Collection | Section 6.5.1 on page 34 |
| VariableFeature | variantDerivation | ZERO OR MORE | IRI | CombinatorialDerivation | Section 6.5.1 on page 34 |
| VariableFeature | variantMeasure | ZERO OR MORE | IRI | om:Measure | Section 6.5.1 on page 34 |
| VariableFeature | variant | ZERO OR MORE | IRI | Component | Section 6.5.1 on page 34 |
| prov:Activity | prov:endedAtTime | ZERO OR ONE | DateTime | — | Section A.1.1 on page 57 |
| prov:Activity | prov:qualifiedUsage | ZERO OR MORE | IRI | prov:Usage | Section A.1.1 on page 57 |
| prov:Activity | prov:startedAtTime | ZERO OR ONE | DateTime | — | Section A.1.1 on page 57 |
| prov:Activity | prov:wasInformedBy | ZERO OR MORE | IRI | prov:Activity | Section A.1.1 on page 57 |
| prov:Activity | type | ZERO OR MORE | IRI | — | Section A.1.1 on page 57 |
| prov:Activity | prov:qualifiedAssociation | ZERO OR MORE | IRI | prov:Association | Section A.1.1 on page 57 |
| prov:Association | prov:agent | EXACTLY ONE | IRI | prov:Agent | Section A.1.3 on page 58 |
| prov:Association | prov:hadRole | ZERO OR MORE | IRI | — | Section A.1.3 on page 58 |
| prov:Association | prov:hadPlan | ZERO OR ONE | IRI | prov:Plan | Section A.1.3 on page 58 |
| prov:Usage | prov:entity | EXACTLY ONE | IRI | — | Section A.1.2 on page 58 |
| prov:Usage | prov:hadRole | ZERO OR MORE | IRI | — | Section A.1.2 on page 58 |
| om:Measure | type | ZERO OR MORE | IRI | — | Section A.2.1 on page 61 |
| om:Measure | om:hasUnit | EXACTLY ONE | IRI | om:Unit | Section A.2.1 on page 61 |
| om:Measure | om:hasNumericalValue | EXACTLY ONE | xsd:float | — | Section A.2.1 on page 61 |
| om:PrefixedUnit | om:hasUnit | EXACTLY ONE | IRI | om:Unit | Section A.2.8 on page 64 |
| om:PrefixedUnit | om:hasPrefix | EXACTLY ONE | IRI | om:Prefix | Section A.2.8 on page 64 |
| om:Prefix | om:alternativeLabels | ZERO OR MORE | String | — | Section A.2.9 on page 64 |
| om:Prefix | om:comment | ZERO OR ONE | String | — | Section A.2.9 on page 64 |
| om:Prefix | om:hasFactor | EXACTLY ONE | xsd:float | — | Section A.2.9 on page 64 |
| om:Prefix | om:label | EXACTLY ONE | String | — | Section A.2.9 on page 64 |
| om:Prefix | om:longcomment | ZERO OR ONE | String | — | Section A.2.9 on page 64 |
| om:Prefix | om:alternativeSymbol | ZERO OR MORE | String | — | Section A.2.9 on page 64 |
| om:Prefix | om:symbol | EXACTLY ONE | String | — | Section A.2.9 on page 64 |
| om:SingularUnit | om:hasUnit | ZERO OR ONE | IRI | om:Unit | Section A.2.3 on page 63 |
| om:SingularUnit | om:hasFactor | ZERO OR ONE | xsd:float | — | Section A.2.3 on page 63 |
| om:UnitDivision | om:hasDenominator | EXACTLY ONE | IRI | om:Unit | Section A.2.6 on page 64 |
| om:UnitDivision | om:hasNumerator | EXACTLY ONE | IRI | om:Unit | Section A.2.6 on page 64 |
| om:UnitExponentiation | om:hasBase | EXACTLY ONE | IRI | om:Unit | Section A.2.7 on page 64 |
| om:UnitExponentiation | om:hasExponent | EXACTLY ONE | xsd:integer | — | Section A.2.7 on page 64 |
| om:UnitMultiplication | om:hasTerm1 | EXACTLY ONE | IRI | om:Unit | Section A.2.5 on page 63 |
| om:UnitMultiplication | om:hasTerm2 | EXACTLY ONE | IRI | om:Unit | Section A.2.5 on page 63 |
| om:Unit | om:alternativeLabels | ZERO OR MORE | String | — | Section A.2.2 on page 62 |
| om:Unit | om:label | EXACTLY ONE | String | — | Section A.2.2 on page 62 |
| om:Unit | om:longcomment | ZERO OR ONE | String | — | Section A.2.2 on page 62 |
| om:Unit | om:symbol | EXACTLY ONE | String | — | Section A.2.2 on page 62 |
| om:Unit | om:alternativeSymbols | ZERO OR MORE | String | — | Section A.2.2 on page 62 |
| om:Unit | om:comment | ZERO OR ONE | String | — | Section A.2.2 on page 62 |

### Rules for the Identified class

**sbol3-10201** ☑ The `displayId` property, if specified, MUST be composed of only alphanumeric or underscore characters and MUST NOT begin with a digit.
Reference: Section 6.1 on page 15

**sbol3-10202** ☑ An `Identified` object MUST NOT refer to itself via its own `prov:wasDerivedFrom` property.

Reference: Section 6.1 on page 15

**sbol3-10203** ● An Identified object MUST NOT form a cyclical chain of references via its prov:wasDerivedFrom property and those of other Identified objects.
Reference: Section 6.1 on page 15

**sbol3-10204** ● Provenance history formed by prov:wasGeneratedBy properties of Identified objects and prov:entity references in prov:Usage objects MUST NOT form circular reference chains.
Reference: Section 6.1 on page 15

**sbol3-10205** ★ An Identified object with a prov:wasGeneratedBy property referring to an prov:Activity with a child prov:Association that has a prov:hadRole property with a value from Table 20 should be of the corresponding type in Table 21.
Reference: Section A.1 on page 55

### *Rules for the TopLevel class*

**sbol3-10301** ☑ If the IRI for the TopLevel object is a URL, then the URL of the hasNamespace property MUST prefix match that URL.
Reference: Section 6.2 on page 16

### *Rules for the Sequence class*

**sbol3-10501** ☑ If the elements property is set, then the encoding property of Sequence MUST be provided.
Reference: Section 6.3 on page 16

**sbol3-10502** ▲ The encoding property of a Sequence MUST indicate how the elements property of the Sequence is to be formed and interpreted.
Reference: Section 6.3 on page 16

**sbol3-10503** ● The elements property of a Sequence MUST be consistent with its encoding property.
Reference: Section 6.3 on page 16

**sbol3-10504** ▲ The encoding property of a Sequence MUST contain a URL from Table 1 if it is well-described by this URL.
Reference: Section 6.3 on page 16

**sbol3-10505** ★ The encoding property of a Sequence SHOULD contain a URL from the textual format (https://identifiers.org/edam:format_2330) branch of the EDAM ontology
Reference: Section 6.3 on page 16

### *Rules for the Component class*

**sbol3-10601** ☑ The set of type properties of a Component MUST NOT have more than one URL from Table 2.
Reference: Section 6.4 on page 18

**sbol3-10602** ▲ Each type property of a Component MUST refer to an ontology term that describes the category of biochemical or physical entity that is represented by the Component.
Reference: Section 6.4 on page 18

**sbol3-10603** ▲ A Component MUST have a type property from Table 2 if it is well-described by this URL.
Reference: Section 6.4 on page 18

**sbol3-10604** ★ A Component SHOULD have a type property that uses the physical entity representation branch of the Systems Biology Ontology.
Reference: Section 6.4 on page 18

**sbol3-10605** ▲ All type properties of a Component MUST refer to non-conflicting ontology terms.
Reference: Section 6.4 on page 18

**sbol3-10606** ▲ If the type property of a Component contains the DNA or RNA type URL listed in Table 2, then its type property MUST contain a URL that refers to a term from the topology attribute branch of the SO, if the topology is known.
Reference: Section 6.4 on page 18

**sbol3-10607** ★ If the type property of a Component contains the DNA or RNA type URL listed in Table 2, then its type property SHOULD also contain at most one URL that refers to a term from the topology attribute branch of the SO.
Reference: Section 6.4 on page 18

**sbol3-10608** ★ A Component SHOULD NOT have a type property that refers to a term from the topology attribute or strand attribute branches of the SO unless it also has a type property with the DNA or RNA type URL listed in Table 2. Reference: Section 6.4 on page 18

**sbol3-10609** ▲ Each role property of a Component MUST refer to an ontology term that is consistent with its type property.
Reference: Section 6.4 on page 18

**sbol3-10610** ▲ Each role property of a Component MUST refer to an ontology term that clarifies the potential function of the Component in a biochemical or physical context.
Reference: Section 6.4 on page 18

**sbol3-10611** ▲ A role property of a Component MUST contain a URL from Table 4 if it is well-described by this URL.
Reference: Section 6.4 on page 18

**sbol3-10612** ★ A role property of a Component SHOULD NOT contain a URL that refers to a term from the sequence feature branch of the SO unless its type property contains the DNA or RNA type URL listed in Table 2.
Reference: Section 6.4 on page 18

**sbol3-10613** ★ If a type property of a Component contains the DNA or RNA type URL, then its role property SHOULD contain exactly one URL that refers to a term from the sequence feature branch of the SO.
Reference: Section 6.4 on page 18

**sbol3-10614** ▲ The Sequence objects referred to by the hasSequence properties of a Component MUST be consistent with each other, such that well-defined mappings exist between their elements properties in accordance with their encoding properties.
Reference: Section 6.4 on page 18

**sbol3-10615** ▲ A hasSequence property of a Component MUST NOT refer to Sequence objects with conflicting encoding properties.
Reference: Section 6.4 on page 18

**sbol3-10616** ● If a hasSequence property of a Component refers to a Sequence object, and one of the type properties of this Component comes from Table 2, then one of the Sequence objects MUST have the encoding that is cross-listed with this type in Table 1.
Reference: Section 6.4 on page 18

**sbol3-10617** ★ If a Component has more than one hasSequence property that refer to Sequence objects with the same encoding, then the elements of these Sequence objects SHOULD have equal lengths.
Reference: Section 6.4 on page 18

### Rules for the Feature class

**sbol3-10701** ▲ Each role property of a Feature MUST refer to a resource that clarifies the intended function of the Feature.
Reference: Section 6.4.1 on page 22

**sbol3-10702** ☑ If a Feature has an orientation property, its URL MUST be drawn from Table 5 or Table 6.
Reference: Section 6.4.1 on page 22

### Rules for the SubComponent class

**sbol3-10801** ☑ If a SubComponent has an roleIntegration property, its URL MUST be drawn from Table 7.
Reference: Section 6.4.1.1 on page 23

**sbol3-10802** ☑ The roleIntegration property of a SubComponent is REQUIRED if the SubComponent has one or more role properties.
Reference: Section 6.4.1.1 on page 23

**sbol3-10803** ☑ The instanceOf property of a SubComponent MUST NOT refer to the same Component as the one that contains the SubComponent.
Reference: Section 6.4.1.1 on page 23

**sbol3-10804** ● SubComponent objects MUST NOT form circular reference chains via their instanceOf properties and the Component objects that contain them.
Reference: Section 6.4.1.1 on page 23

**sbol3-10805** ☑ The set of Location objects referred to by the hasLocation properties of a single SubComponent MUST NOT specify overlapping regions.
Reference: Section 6.4.1.1 on page 23

**sbol3-10806** ☑ If a SubComponent object has at least one hasLocation and sourceLocation properties, then the sum of the lengths of the Location objects referred to by the hasLocation properties MUST equal the sum of the lengths of the Location objects referred to by the sourceLocation properties.
Reference: Section 6.4.1.1 on page 23

**sbol3-10807** ● If a SubComponent object has at least one hasLocation and zero sourceLocation properties, and the Component linked by its instanceOf has precisely one hasSequence property whose Sequence has a value for its elements property, then the sum of the lengths of the Location objects referred to by the hasLocation properties MUST equal the length of the elements value of the Sequence.
Reference: Section 6.4.1.1 on page 23

### Rules for the ComponentReference class

**sbol3-10901** ☑ If a ComponentReference object is a child of a Component, then its inChildOf property MUST be a SubComponent of its parent.
Reference: Section 6.4.1.2 on page 24

**sbol3-10902** ☑ If a ComponentReference object is a child of another ComponentReference, via the refersTo property, then its inChildOf property MUST be a SubComponent of the Component referred to by the instanceOf property of the SubComponent referred to by the parent's inChildOf property.
Reference: Section 6.4.1.2 on page 24

**sbol3-10903** ☑ If the refersTo property of a ComponentReference refers to another ComponentReference, then the second ComponentReference MUST be either a child of the first ComponentReference or a child of the Component referred to by the instanceOf property of the SubComponent referred to by the inChildOf property of the first ComponentReference.
Reference: Section 6.4.1.2 on page 24

**sbol3-10904** ☑ If the refersTo property of a ComponentReference refers to a Feature of any other type besides ComponentReference, then that Feature MUST be a child of the Component referred to by the instanceOf property of the SubComponent referred to by the inChildOf property of the first ComponentReference.
Reference: Section 6.4.1.2 on page 24

### *Rules for the LocalSubComponent class*

**sbol3-11001** ☑ A LocalSubComponent MUST NOT have more than one URL from Table 2.
Reference: Section 6.4.1.3 on page 25

**sbol3-11002** ▲ Each type property of a LocalSubComponent MUST refer to an ontology term that describes the category of biochemical or physical entity that is represented by the LocalSubComponent.
Reference: Section 6.4.1.3 on page 25

**sbol3-11003** ▲ A LocalSubComponent MUST have a type property from Table 2 if it is well-described by this URL.
Reference: Section 6.4.1.3 on page 25

**sbol3-11004** ★ A LocalSubComponent SHOULD have a type property from Table 2.
Reference: Section 6.4.1.3 on page 25

**sbol3-11005** ▲ All type properties of a LocalSubComponent MUST refer to non-conflicting ontology terms.
Reference: Section 6.4.1.3 on page 25

**sbol3-11006** ▲ If the type property of a LocalSubComponent contains the DNA or RNA type URL listed in Table 2, then its type property MUST contain a URL that refers to a term from the topology attribute branch of the SO, if the topology is known.
Reference: Section 6.4.1.3 on page 25

**sbol3-11007** ★ If the type property of a LocalSubComponent contains the DNA or RNA type URL listed in Table 2, then its type property SHOULD also contain at most one URL that refers to a term from the topology attribute branch of the SO.
Reference: Section 6.4.1.3 on page 25

**sbol3-11008** ★ A LocalSubComponent SHOULD NOT have a type property that refers to a term from the topology attribute or strand attribute branches of the SO unless it also has a type property with the DNA or RNA type URL listed in Table 2. Reference: Section 6.4.1.3 on page 25

**sbol3-11009** ▲ Each role property of a LocalSubComponent MUST refer to an ontology term that is consistent with its type property.
Reference: Section 6.4 on page 18

**sbol3-11010** ▲ A role property of a LocalSubComponent MUST contain a URL from Table 4 if it is well-described by this URL.
Reference: Section 6.4 on page 18

**sbol3-11011** ★ A role property of a LocalSubComponent SHOULD NOT contain a URL that refers to a term from the sequence feature branch of the SO unless its type property contains the DNA or RNA type URL listed in Table 2.
Reference: Section 6.4 on page 18

**sbol3-11012** ★ If a type property of a LocalSubComponent contains the DNA or RNA type URL, then its role property SHOULD contain exactly one URL that refers to a term from the sequence feature branch of the SO.
Reference: Section 6.4 on page 18

**sbol3-11013** ☑ The set of Location objects referred to by the hasLocation properties of a single LocalSubComponent MUST NOT specify overlapping regions.
Reference: Section 6.4.1.3 on page 25

### Rules for the ExternallyDefined class

**sbol3-11101** ☑ A ExternallyDefined MUST NOT have more than one URL from Table 2.
Reference: Section 6.4.1.4 on page 25

**sbol3-11102** ▲ Each type property of a ExternallyDefined MUST refer to an ontology term that describes the category of biochemical or physical entity that is represented by the Component.
Reference: Section 6.4.1.4 on page 25

**sbol3-11103** ▲ A ExternallyDefined MUST have a type property from Table 2 if it is well-described by this URL.
Reference: Section 6.4.1.4 on page 25

**sbol3-11104** ★ A ExternallyDefined SHOULD have a type property from Table 2.
Reference: Section 6.4.1.4 on page 25

**sbol3-11105** ▲ All type properties of a ExternallyDefined MUST refer to non-conflicting ontology terms.
Reference: Section 6.4.1.4 on page 25

**sbol3-11106** ▲ If the type property of a ExternallyDefined contains the DNA or RNA type URL listed in Table 2, then its type property MUST contain a URL that refers to a term from the topology attribute branch of the SO, if the topology is known.
Reference: Section 6.4.1.4 on page 25

**sbol3-11107** ★ If the type property of a ExternallyDefined contains the DNA or RNA type URL listed in Table 2, then its type property SHOULD also contain at most one URL that refers to a term from the topology attribute branch of the SO.
Reference: Section 6.4.1.4 on page 25

**sbol3-11108** ★ A ExternallyDefined SHOULD NOT have a type property that refers to a term from the topology attribute or strand attribute branches of the SO unless it also has a type property with the DNA or RNA type URL listed in Table 2. Reference: Section 6.4.1.4 on page 25

**sbol3-11109** ▲ The URL contained by the definition property of a ExternallyDefined SHOULD refer to an external resource in Section Section 7.6 when the object is defined in one of these resources.
Reference: Section 6.4.1.4 on page 25

### Rules for the SequenceFeature class

**sbol3-11201** ☑ The set of Location objects referred to by the hasLocation properties of a single SequenceFeature MUST NOT specify overlapping regions.
Reference: Section 6.4.1.5 on page 25

### Rules for the Location class

**sbol3-11301** ☑ If a Location has an orientation property, its URL MUST be drawn from Table 5 or Table 6.
Reference: Section 6.4.2 on page 26

**sbol3-11302** ☑ For every Location that is not an EntireSequence and that is the value of a hasLocation property of a Feature, the value of its hasSequence property MUST also either be a value of the hasSequence property of the parent Component or else be the value of some hasSequence property of an EntireSequence that is also a child of the same Component.
Reference: Section 6.4.2 on page 26

**sbol3-11303** ● For every Location that is not an EntireSequence and that is the value of a sourceLocation property of a SubComponent, the value of its hasSequence property MUST also either be a value of the hasSequence property of the Component linked by its parent's instanceOf property or else be the value of some hasSequence property of an EntireSequence that is also a child of the same Component linked by instanceOf.
Reference: Section 6.4.2 on page 26

### *Rules for the Range class*

**sbol3-11401** ☑ The value of the start property of a Range MUST be greater than zero and less than or equal to the length of the elements value of the Sequence referred to by its hasSequence property.
Reference: Section 6.4.2.1 on page 27

**sbol3-11402** ☑ The value of the end property of a Range MUST be greater than zero and less than or equal to the length of the elements value of theSequence referred to by its hasSequence property.
Reference: Section 6.4.2.1 on page 27

**sbol3-11403** ☑ The value of the end property of a Range MUST be greater than or equal to the value of its start property.
Reference: Section 6.4.2.1 on page 27

### *Rules for the Cut class*

**sbol3-11501** ☑ The value of the at property of a Cut MUST be greater than or equal to zero and less than or equal to the length of the elements value of the Sequence referred to by its hasSequence property.
Reference: Section 6.4.2.2 on page 27

### *Rules for the Constraint class*

**sbol3-11701** ☑ The Feature referenced by the subject property of a Constraint MUST be contained by the Component that contains the Constraint.
Reference: Section 6.4.3 on page 27

**sbol3-11702** ☑ The Feature referenced by the object property of a Constraint MUST be contained by the Component that contains the Constraint.
Reference: Section 6.4.3 on page 27

**sbol3-11703** ☑ The object property of a Constraint MUST NOT refer to the same SubComponent as the subject property of the Constraint.
Reference: Section 6.4.3 on page 27

**sbol3-11704** ★ The value of the restriction property of a Constraint SHOULD be drawn from Table 8, Table 9, or Table 10.
Reference: Section 6.4.3 on page 27

**sbol3-11705** ● If the restriction property of a Constraint is drawn from Table 8, then the Feature objects referred to by the subject and object properties MUST comply with the relation specified in Table 8.

**sbol3-11706** ● If the restriction property of a Constraint is drawn from Table 10 and if the Feature objects referred to by the subject and object properties both have hasLocation properties with Location objects whose hasSequence property refers to the same Sequence, then the positions of the referred Location objects MUST comply with the relation specified in Table 10.

### Rules for the Interaction class

**sbol3-11801** ▲ Each type property of an Interaction MUST refer to an ontology term that describes the behavior represented by the Interaction.
Reference: Section 6.4.4 on page 28

**sbol3-11802** ▲ All type properties of an Interaction MUST refer to non-conflicting ontology terms.
Reference: Section 6.4.4 on page 28

**sbol3-11803** ★ Exactly one type property of an Interaction SHOULD refer to a term from the occurring entity relationship branch of the SBO.
Reference: Section 6.4.4 on page 28

**sbol3-11804** ★ If the hasParticipation properties of an Interaction refer to one or more Participation objects, and one of the type properties of this Interaction comes from Table 11, then the Participation objects SHOULD have a role from the set of role properties that is cross-listed with this type in Table 12.
Reference: Section 6.4.4 on page 28

### Rules for the Participation class

**sbol3-11901** ☑ A Participation MUST contain precisely one participant or higherOrderParticipant property.
Reference: Section 6.4.4.1 on page 31

**sbol3-11902** ☑ The Feature referenced by the participant property of a Participation MUST be contained by the Component that contains the Interaction that contains the Participation.
Reference: Section 6.4.4.1 on page 31

**sbol3-11903** ☑ The Interaction referenced by the higherOrderParticipant property of a Participation MUST be contained by the Component that contains the Interaction that contains the Participation.
Reference: Section 6.4.4.1 on page 31

**sbol3-11904** ▲ Each role property of a Participation MUST refer to an ontology term that describes the behavior represented by the Participation.
Reference: Section 6.4.4.1 on page 31

**sbol3-11905** ▲ All role properties of a Participation MUST refer to non-conflicting ontology terms.
Reference: Section 6.4.4.1 on page 31

**sbol3-11906** ★ Exactly one role in the set of role properties SHOULD be a URL from the participant role branch of the SBO (see Table 12).
Reference: Section 6.4.4.1 on page 31

### Rules for the Interface class

**sbol3-12001** ☑ The Feature referenced by the input property of an Interface MUST be contained by the Component that contains the Interface.
Reference: Section 6.4.5 on page 32

**sbol3-12002** ☑ The Feature referenced by the output property of an Interface MUST be contained by the Component that contains the Interface.
Reference: Section 6.4.5 on page 32

**sbol3-12003** ☑ The Feature referenced by the nondirectional property of an Interface MUST be contained by the Component that contains the Interface.
Reference: Section 6.4.5 on page 32

### *Rules for the CombinatorialDerivation class*

**sbol3-12101** ☑ The strategy property of a CombinatorialDerivation, if specified, MUST contain a URL from Table 13.
Reference: Section 6.5 on page 33

**sbol3-12102** ☑ If the strategy property of a CombinatorialDerivation contains the URL http://sbols.org/v3#enumerate, then its hasVariableFeature property MUST NOT contain a VariableFeature with an cardinality property that contains the URL http://sbols.org/v3#zeroOrMore or the URL http://sbols.org/v3#oneOrMore.
Reference: Section 6.5 on page 33

**sbol3-12103** ☑ A CombinatorialDerivation MUST NOT contain two or more hasVariableFeature properties that refer to VariableFeature objects with a variable property that contain the same IRI.
Reference: Section 6.5 on page 33

**sbol3-12104** ★ A CombinatorialDerivation's template Component SHOULD contain one or more hasFeature properties.
Reference: Section 6.5 on page 33

**sbol3-12105** ★ If the prov:wasDerivedFrom property of a Component refers to a CombinatorialDerivation, then the prov:wasDerivedFrom properties of each child Feature of the Component should refer to a Feature in the template Component of the CombinatorialDerivation
Reference: Section 6.5 on page 33

**sbol3-12106** ★ If the prov:wasDerivedFrom property of a Collection refers to a CombinatorialDerivation, then the prov:wasDerivedFrom properties of the objects that are referred to by its member properties SHOULD also refer to the CombinatorialDerivation.
Reference: Section 6.5 on page 33

**sbol3-12107** ★ If the prov:wasDerivedFrom property of a Component refers to a CombinatorialDerivation, then the type properties of this Component SHOULD contain all IRIs contained by the type properties of the template Component of the CombinatorialDerivation.
Reference: Section 6.5 on page 33

**sbol3-12108** ★ If the prov:wasDerivedFrom property of a Component refers to a CombinatorialDerivation, then the role properties of this Component SHOULD contain all IRIs contained by the role properties of the template Component of the CombinatorialDerivation.
Reference: Section 6.5 on page 33

**sbol3-12109** ● If the prov:wasDerivedFrom property of a Component refers to a CombinatorialDerivation, then for any Feature in the Component with a prov:wasDerivedFrom property referring to a static Feature in the template Component of the CombinatorialDerivation, that derived Feature MUST have properties identical to those of the static Feature.
Reference: Section 6.5 on page 33

**sbol3-12110** ★ If the prov:wasDerivedFrom property of a Component refers to a CombinatorialDerivation, then each static Feature in the template Component SHOULD be referred to by a prov:wasDerivedFrom property from exactly one Feature in the derived Component.
Reference: Section 6.5 on page 33

**sbol3-12111** ★ If the prov:wasDerivedFrom property of a Component refers to a CombinatorialDerivation, then each variable Feature in the template Component SHOULD be referred to by a prov:wasDerivedFrom property from a number of Feature objects in the derived Component that is compatible with the cardinality property of the corresponding VariableFeature.
Reference: Section 6.5 on page 33

**sbol3-12112** ● If the prov:wasDerivedFrom property of a Component refers to a CombinatorialDerivation, then for any SubComponent in the Component with a prov:wasDerivedFrom property referring to a variable Feature in the template Component of the CombinatorialDerivation, that derived SubComponent MUST have an instanceOf property that refers to a Component specified by the corresponding VariableFeature. In particular, that Component must be a value of the variant property, a member or recursive member of a Collection that is a value of the variantCollection property, or a Component with a prov:wasDerivedFrom property that refers to a CombinatorialDerivation specified by a variantDerivation property of the VariableFeature.
Reference: Section 6.5 on page 33

**sbol3-12113** ● If the prov:wasDerivedFrom property of a Component refers to a CombinatorialDerivation and the template Component of the CombinatorialDerivation contains Constraint objects, then for any Feature contained by the Component that has a prov:wasDerivedFrom property that refers to the subject or object Feature of any of the template Constraint objects, that feature MUST adhere to the restriction properties of the template Constraint objects.
Reference: Section 6.5 on page 33

**sbol3-12114** ★ If the prov:wasDerivedFrom property of a Component refers to a CombinatorialDerivation, then for any Feature in the Component with a prov:wasDerivedFrom property referring to a variable Feature in the template Component of the CombinatorialDerivation, then the role properties of that Feature SHOULD contain all IRIs contained by the role properties of the template Feature.
Reference: Section 6.5 on page 33

**sbol3-12115** ★ Let the type-determining referent of a Feature be the Feature itself for a LocalSubComponent or ExternallyDefined, the Component referred by the instanceOf property of a SubComponent and the type-determining referent of the Feature referred to be a ComponentReference.
If the prov:wasDerivedFrom property of a Component refers to a CombinatorialDerivation, then for any Feature in the Component with a prov:wasDerivedFrom property referring to a variable Feature in the template Component of the CombinatorialDerivation, then the type properties of the Feature's type-determining referent SHOULD contain all IRIs contained by the type properties of the template Feature's type-determining referent.
Reference: Section 6.5 on page 33

### *Rules for the VariableFeature class*

**sbol3-12201** ☑ The IRI contained by the cardinality property of a VariableFeature MUST come from Table 14.
Reference: Section 6.5.1 on page 34

**sbol3-12202** ● The Feature referenced by the variable property of a VariableFeature MUST be contained by the template Component of the CombinatorialDerivation that contains the VariableFeature.
Reference: Section 6.5.1 on page 34

**sbol3-12203** ● The `member` properties of a `Collection` that is referred to by the `variantCollection` property of a `VariableFeature` MUST refer only to `Component` objects or to `Collection` objects that themselves contain only `Component` or `Collection` objects, recursively.
Reference: Section 6.5.1 on page 34

**sbol3-12204** ● `VariableFeature` objects MUST NOT form circular reference chains via their `variantDerivation` properties and parent `CombinatorialDerivation` objects.
Reference: Section 6.5.1 on page 34

### *Rules for the Implementation class*

**sbol3-12301** ▲ Each `prov:wasDerivedFrom` property of an `Implementation` MUST refer to a `Component` that contains a description of the intended nature of the actual object indicated by the `Implementation`.
Reference: Section 6.6 on page 36

**sbol3-12302** ▲ All `prov:wasDerivedFrom` properties of an `Implementation` MUST refer to non-conflicting `Component` descriptions.
Reference: Section 6.6 on page 36

**sbol3-12303** ▲ If the `built` property of an `Implementation` is set, then the `Component` it refers to MUST be a faithful description of the actual object indicated by the `Implementation`.
Reference: Section 6.6 on page 36

### *Rules for the Model class*

**sbol3-12501** ▲ The `IRI` contained by the `source` property of a `Model` MUST specify the location of the model's source file.
Reference: Section 6.8 on page 37

**sbol3-12502** ▲ The `IRI` contained by the `language` property of a `Model` MUST specify the language in which the model is encoded.
Reference: Section 6.8 on page 37

**sbol3-12503** ▲ The `language` property of a `Model` MUST contain a `URL` from Table 15 if it is well-described by this `URL`.
Reference: Section 6.8 on page 37

**sbol3-12504** ★ The `language` property of a `Model` SHOULD contain a `URL` that refers to a term from the EDAM ontology.
Reference: Section 6.8 on page 37

**sbol3-12505** ▲ The `IRI` contained by the `framework` property of a `Model` MUST specify the modeling framework of the model.
Reference: Section 6.8 on page 37

**sbol3-12506** ▲ The `framework` property of a `Model` MUST contain a `URL` from Table 16 if it is well-described by this `URL`.
Reference: Section 6.8 on page 37

**sbol3-12507** ★ The `framework` property SHOULD contain a `URL` that refers to a term from the modeling framework branch of the SBO.
Reference: Section 6.8 on page 37

### Rules for the Attachment class

**sbol3-12801** ▲ The `source` property of an `Attachment` MUST specify the location of the model's source file.
Reference: Section 6.10 on page 39

**sbol3-12802** ▲ The `IRI` contained by the `format` property of an `Attachment` MUST specify the file type of the attachment.
Reference: Section 6.10 on page 39

**sbol3-12803** ★ The `format` property of an `Attachment` SHOULD contain a `URL` that refers to a term from the EDAM ontology.
Reference: Section 6.10 on page 39

**sbol3-12804** ▲ The `size` property, if specified, MUST indicate file size in bytes.
Reference: Section 6.10 on page 39

**sbol3-12805** ▲ The `hash` property, if specified, MUST be a hash value for the file contents represented as a hexadecimal digest.
Reference: Section 6.10 on page 39

**sbol3-12806** ▲ The `hashAlgorithm`, if specified, MUST be the name of a hash algorithm used to generate the value of the `hash` property.
Reference: Section 6.10 on page 39

**sbol3-12807** ★ The `hashAlgorithm` property of an `Attachment` SHOULD be a hash name `String` from the IANA Named Information Hash Algorithm Registry, of which `sha3-256` is currently RECOMMENDED.
Reference: Section 6.10 on page 39

**sbol3-12808** ☑ If the `hash` property is set, then the `hashAlgorithm` MUST be set as well.
Reference: Section 6.10 on page 39

### Rules for the prov:Activity class

**sbol3-12901** ★ An `prov:Activity` with a `type` from Table 20 SHOULD NOT have child `prov:Usage` objects that have `prov:hadRole` properties from Table 20 other than the same `URL` or the `URL` of the preceding stage given in Table 21.
Reference: Section A.1.1 on page 57

**sbol3-12902** ★ If an `prov:Activity` has a `type` property with a value from Table 20, then every child `prov:Association` SHOULD have a `prov:hadRole` property with the same value.
Reference: Section A.1.1 on page 57

### Rules for the prov:Usage class

**sbol3-13001** ★ If a `prov:Usage` has a `prov:hadRole` property with a value from Table 20, then its `prov:entity` property SHOULD refer to an object of the corresponding type in Table 21.
Reference: Section A.1.2 on page 58

### Rules for the om:Measure class

**sbol3-13401** ★ If a `om:Measure` includes a `type` property, then exactly one of the `IRI`s that this property contains SHOULD refer to a term from the systems description parameter branch of the SBO.
Reference: Section A.2.1 on page 61

### Rules for the om:Unit class

**sbol3-13501** ★ If both of the `name` property and `om:label` properties of a `om:Unit` are non-empty, then they SHOULD contain identical `String`s.
Reference: Section A.2.2 on page 62

**sbol3-13502** ★ If both of the `description` property and `om:comment` properties of a `om:Unit` are non-empty, then they SHOULD contain identical `String`s.
Reference: Section A.2.2 on page 62

### Rules for the om:Prefix class

**sbol3-14201** ★ If both of the `name` property and `om:label` properties of a `om:Prefix` are non-empty, then they SHOULD contain identical `String`s.
Reference: Section A.2.9 on page 64

**sbol3-14202** ★ If both of the `description` property and `om:comment` properties of a `om:Prefix` are non-empty, then they SHOULD contain identical `String`s.
Reference: Section A.2.9 on page 64
